# Synthesis, Antibacterial Activity, and Mechanism of C-6 Aminated β-Carboline Derivatives Against MRSA

**DOI:** 10.3390/antibiotics15040339

**Published:** 2026-03-26

**Authors:** Qiuran Wei, Weida Liang, Hongda Qiu, Xing Zhao, Yang Li, Han Ouyang, Bowen Han, Lingling Zhao, Xiao Wang, Hongze Liang

**Affiliations:** 1 School of Materials Science and Chemical Engineering, Ningbo University, Ningbo 315211, China; wqr1880898@163.com (Q.W.); liangweida94@gmail.com (W.L.); qiuhongda1998@163.com (H.Q.); 15167214127@163.com (X.Z.); zhaolingling@nbu.edu.cn (L.Z.); 2 Health Science Center, Ningbo University, Ningbo 315211, China; 3 Institute of Drug Discovery Technology, Ningbo University, Ningbo 315211, China; liyang@nbu.edu.cn (Y.L.); ouyanghan@nbu.edu.cn (H.O.); hanbowen@nbu.edu.cn (B.H.)

**Keywords:** β-carboline, antibacterial, MRSA, mechanism, antibiofilm

## Abstract

**Background:** The escalating spread of drug-resistant bacteria is intensifying the antibiotic resistance crisis, necessitating the urgent development of novel antimicrobial agents to address the resulting high global mortality rates and significant socioeconomic burden. **Objectives:** This study aimed to aminate the C-6 position of β-carboline and investigate the antibacterial activity and mechanism of action of the derivatives. **Results:** For the first time, 16 derivatives with various nitrogen-containing moieties, including aliphatic- and phenyl-amino, imidazolium, pyridinium, and quinolinium, were synthesized via amination at the C-6 position of β-carboline. These compounds exhibited moderate to good activity against Gram-positive methicillin-resistant *Staphylococcus aureus* (MRSA) and *Bacillus subtilis*, with minimum inhibitory concentration (MIC) values ranging from 1.56 to 100 μg/mL. The study reveals that elongating an alkyl chain, incorporating a cationic scaffold, and expanding a π-delocalized system can enhance antibacterial activity. The most potent derivative from each series was selected for further mechanistic investigation against MRSA. All studied compounds demonstrated low hemolytic activity and low cytotoxicity. Studies on the antibacterial mechanism indicated that the compounds exert their antibacterial effects by disrupting bacterial cell walls and membranes. Additionally, two of the compounds were found to potentially disrupt the secondary structure of DNA. All tested compounds exhibited antibiofilm activity. **Conclusions:** Our findings demonstrate that amination modification at the C-6 position of β-carboline can enhance antibacterial activity by disrupting the cell wall membranes and interacting with bacterial DNA. These results provide a basis for further optimization of antibacterial agents based on β-carboline.

## 1. Introduction

As antibiotic resistance rates continue to escalate, the increasing prevalence of drug-resistant bacteria poses a significant and mounting threat to human health. These pathogens can be transmitted to humans via the food chain, contaminated water sources, and other pathways [1,2]. The escalating challenge of bacterial drug resistance has rendered existing antibiotics increasingly inadequate in combating the growing number of resistant strains. As a result, numerous infections that were previously treatable now present potentially fatal threats. Key pathogens such as methicillin-resistant *Staphylococcus aureus* (MRSA) have developed high-level resistance to conventional antibiotic therapies through multidrug resistance mechanisms [3,4,5,6]. A 2022 global study published in *The Lancet* estimates that antimicrobial resistance (AMR) is responsible for approximately 1.27 million deaths annually [7]. According to the World Health Organization (WHO), the death toll could reach 10 million annually by 2050 if no action is taken [8]. This would not only represent a massive escalation of the humanitarian crisis but also result in substantial global economic losses. Therefore, accelerating the development of novel antimicrobial drugs is of critical urgency. This is not only key to addressing current clinical treatment challenges but also an essential imperative for safeguarding global public health security.

Alkaloids are a class of naturally occurring nitrogen-containing organic compounds primarily found in plants. They are also present in some microorganisms and animals. They have long attracted significant attention in the pharmaceutical field, as many alkaloids have been confirmed to exhibit remarkable antimicrobial activity. A prominent clinical example is berberine, an alkaloid isolated from herbs such as *Coptis chinensis*. It displays potent inhibitory activity against intestinal pathogens such as *Escherichia coli* and *Shigella flexneri*, and is commonly used in the treatment of intestinal infectious diseases, including diarrhea and dysentery [9,10]. Structural modification and optimization of natural alkaloids enable the development of novel antimicrobial agents with potentiated activity, reduced toxicity, and improved stability, making this strategy a key research focus in recent years [11,12,13].

β-Carboline, a class of indole alkaloids possessing diverse biochemical and pharmacological activities, serves as a potential lead compound for drug development [14]. This compound has been obtained from diverse natural sources such as marine organisms [15,16,17] and plants [18,19,20,21]. Featuring a distinct structural framework, β-carboline displays a range of biological activities, including anticancer [22], antiviral [23], and antifungal [24] effects. Recent years have witnessed a growing body of research focused on modifying β-carbolines to improve their biological activity. Wang synthesized a series of 3-methyl-β-carboline derivatives and observed that introducing a one- or three-carbon chain at the C-1 position enhanced the potency of the compounds [25]. Formagio et al. synthesized β-carboline derivatives bearing an amino group at the C-3 position, thereby enhancing the antibacterial activity [26]. Wang et al. found that N-2 quaternization significantly enhanced antibacterial potency [27], while He et al. modified the N-9 position with cinnamic acid derivatives and observed enhanced activity against Gram-positive bacteria [28]. These studies collectively demonstrate that modifications at various positions of the β-carboline scaffold can modulate biological activity. While modifications at the C-1, C-3, N-2, and N-9 positions have been extensively studied, the C-6 position has received comparatively less attention, with only a few studies exploring the potential of C-6-derived reagents. Lunagariya et al. reported that varying the substituent at the C-6 position influenced the cytotoxic activity of β-carboline derivatives [29]. Previous studies have found that amine-containing molecules most readily penetrate Gram-negative bacteria to exert bactericidal effects [30], while heterocyclic cations are frequently employed as core scaffolds in antibacterial agents due to their ability to interact with bacterial membranes and DNA [31,32,33].

Since investigations into C-6 amination of β-carbolines for antibacterial applications remain limited, and building upon our previous findings [34,35], we attempted to introduce various nitrogen-containing groups (including aliphatic- and phenyl-amino, imidazolium, pyridinium, and quinolinium) at the C-6 position. These β-carboline derivatives were synthesized and evaluated for their antibacterial activity, and the underlying mechanisms were also explored. This work aimed to provide new insights into the antimicrobial potential of β-carbolines and to support further optimization of this scaffold.

## 2. Results and Discussion

### 2.1. Synthesis of 6-Aminated β-Carboline Derivatives

**Scheme 1:**
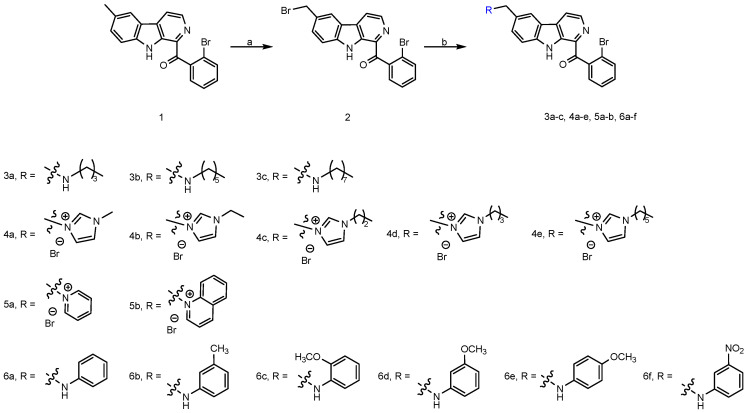
Synthesis of 6-aminated β-carboline derivatives. Reagents and conditions: (a) NBS, CCl_4_, BPO, reflux overnight; (b) **3a–c:** different aliphatic amines, Cs_2_CO_3_, DMF, room temperature; **4a–e:** different imidazoles, CH_3_CN, reflux; **5a–b:** pyridine or quinoline, CH_3_CN, reflux; **6a–f:** different anilines, DMF, K_2_CO_3_, reflux.

The synthetic methods of 6-aminated β-carboline derivatives are described in Scheme 1. Compounds **1** and **2** were synthesized according to reported procedures [36,37,38], with modifications applied to the synthesis of compound **2**. Briefly, compound **1** and N-bromosuccinimide (NBS) were refluxed overnight in the carbon tetrachloride and benzoyl peroxide (BPO) system to afford compound **2**. In practice, we found that compound **2** tended to decompose during purification via silica gel column chromatography. Consequently, it was crudely isolated, confirmed by HRMS (Figure S1), and directly used in subsequent reactions to synthesize target products **3a–c**, **4a–e**, **5a–b**, and **6a–f**. The target products were synthesized and purified following reported procedures [39,40,41,42,43]. All compounds were characterized by ^1^H NMR, ^13^C NMR, HRMS, and HPLC.

### 2.2. Antimicrobial Activity of Compounds

#### 2.2.1. Antimicrobial Activity

The antibacterial activity of 6-aminated β-carboline derivatives (**3a–c**, **4a–e**, **5a–b**, **6a–f**) was evaluated using the broth microdilution method. The minimum inhibitory concentrations (MICs) of these compounds were determined against two representative Gram-positive and two representative Gram-negative bacteria. Vancomycin and colistin were employed as reference antibacterial agents to validate the assay protocols and for comparative analysis.

The results, summarized in Table 1, revealed that none of the synthesized compounds exhibited activity against Gram-negative bacteria, while aniline-modified carbolines (6a–f) similarly showed no inhibitory effects on Gram-positive bacteria (MIC > 200 μg/mL). In contrast, carbolines functionalized with aliphatic amines or nitrogen heterocycles displayed moderate to good antibacterial activity (MIC < 100 μg/mL). Based on previous research findings [34], these modified β-carboline derivatives demonstrated enhanced antibacterial activity compared with the parent compound **1**.

Among all compounds bearing the three types of active substituent, compounds **3c**, **4e**, and **5b** showed better activity. The most potent compound, **3c**, showed MIC values of 1.56 μg/mL and 3.125 μg/mL against MRSA ATCC43300 and *Bacillus subtilis* ATCC336690, respectively. Compound **4e** exhibited MIC values of 6.25 μg/mL and 12.5 μg/mL against the same two strains, while compound **5b** showed favorable activity against MRSA ATCC43300 (12.5 μg/mL) and *Bacillus subtilis* ATCC336690 (25 μg/mL).

Further analysis of the structure–activity relationship (SAR) revealed that among the aliphatic amine-modified derivatives (compounds **3a–c**), the antibacterial activity gradually increased with extension of the alkyl chain length. Similarly, among the imidazolium-modified derivatives, the length of the alkyl chain attached to the nitrogen atom also showed a positive correlation with activity. The N-alkylimidazolium-substituted agents **4** demonstrated that introduction of a cationic moiety further enhances the antibacterial potency [44]. Higher delocalization of the π-electron system may show a significant impact on antimicrobial activity, as revealed by **5b** (MIC, 12.5 μg/mL) vs. **5a** (MIC, 100 μg/mL). These findings suggest that tuning the length of the alkyl chain, the nature of the cationic scaffold, and the system of π delocalization may endow the resulting agents enhanced antibacterial efficacy, thus providing direction for further structural optimization [45,46].

To elucidate the differential activity profiles of our compounds against Gram-positive and Gram-negative bacteria, we measured the zeta potential of *E. coli* following treatment with representative compounds **3c**, **4e**, and **5b**. As shown in Figure S2, the zeta potential of *E. coli* remained almost unchanged upon compound treatment, indicating negligible electrostatic interaction between the derivatives and the bacterial surface. This observation suggests that the outer membrane of Gram-negative bacteria acts as an effective barrier, preventing the compounds from accessing the cell surface and subsequent intracellular targets [47]. In contrast, Gram-positive bacteria lack this outer membrane, allowing cationic compounds to interact with the cytoplasmic membrane and penetrate the cell [48], which is consistent with their antibacterial activity observed against MRSA and *B. subtilis*. These findings are in line with previous reports that the outer-membrane permeability barrier is a major determinant of Gram-negative resistance to many antimicrobial agents [49]. The negligible change in zeta potential further confirms that the compounds are unable to effectively interact with or traverse the outer membrane, explaining their lack of activity against Gram-negative strains.

#### 2.2.2. Assessment of Bactericidal Efficacy

MRSA is a leading cause of both hospital-acquired and community-associated infections worldwide, with limited treatment options due to its resistance to multiple antibiotics [50]. Moreover, based on the initial screening results, our C-6 aminated β-carboline derivatives demonstrated the most potent activity against MRSA compared to other tested strains. Therefore, we chose MRSA as the experimental strain for the subsequent antibacterial experiments.

Based on the MIC results, compounds **3c**, **4e**, and **5b** were selected for the determination of minimum bactericidal concentrations (MBCs) against MRSA ATCC43300, with vancomycin serving as the standard reference [51]. According to the Clinical and Laboratory Standards Institute (CLSI) guidelines, an MBC/MIC ratio ≤ 4 is considered bactericidal, whereas a ratio ≥ 8 is regarded as bacteriostatic. As shown in Table 2, compound **3c** exhibited an MBC/MIC ratio greater than 4, indicating a bacteriostatic effect against MRSA ATCC43300. In contrast, compounds **4e** and **5b** showed MBC/MIC ratios below 4, demonstrating bactericidal activity against this strain.

#### 2.2.3. Kinetics of the Bactericidal Activity

**Figure 1:**
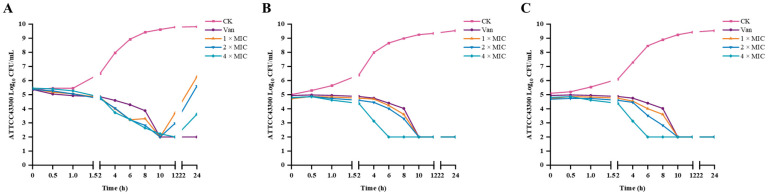
Time–killing curves of **3c** (**A**), **4e** (**B**), and **5b** (**C**) against MRSA ATCC43300. CK: 0.4% DMSO. The results are given as means ± SD (*n* = 3).

As demonstrated in Figure 1, the negative control group of MRSA ATCC43300 exhibited a rapid increase in bacterial load (log_10_ CFU/mL) within 2 h, reaching 9.5 log_10_ CFU/mL by 24 h. All tested carboline derivatives effectively inhibited the growth of MRSA ATCC43300 within the first 10 h at the concentrations examined. However, following treatment with compound **3c**, regrowth of MRSA was observed after 10 h, with bacterial counts returning to levels near the initial inoculum at 24 h. This indicates that **3c** is unable to achieve complete eradication of the bacterial population, especially metabolically inactive persister cells. In contrast, compounds **4e** and **5b** sustained stable antibacterial activity from 10 to 24 h. At a higher concentration (4 × MIC), both **4e** and **5b** induced a sharp decline in bacterial counts between 2 and 6 h. Compared with vancomycin (positive control), all three compounds displayed more rapid antibacterial kinetics.

### 2.3. Evaluation of the Potential of Resistance Development

**Figure 2:**
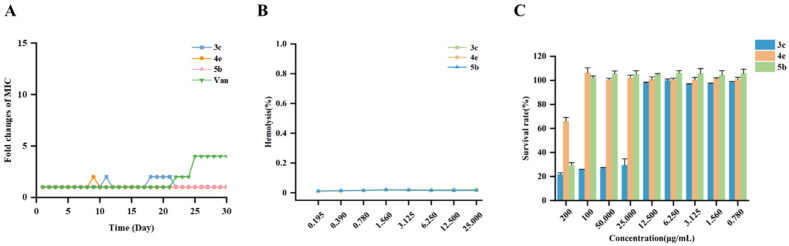
Antimicrobial activity and toxicity of derivatives. (**A**) Bacterial resistance evaluation of **3c**, **4e**, **5b** and vancomycin against MRSA ATCC43300. (**B**) Hemolytic activity of derivatives against fresh human red blood cells. (**C**) Cytotoxicity of **3c**, **4e**, and **5b** against RAW264.7 cells. The results are given as means ± SD (*n* = 3).

The emergence of bacterial resistance poses a serious threat to human health. Therefore, we evaluated the potential of the selected compounds to induce resistance in MRSA ATCC43300. As shown in Figure 2A, after 30 generations of cultivation at subinhibitory concentrations (1/4 × MIC), none of the tested compounds exhibited a significant trend toward resistance development. In contrast, the MIC of vancomycin against MRSA increased by fourfold after 25 days, indicating the development of resistance.

### 2.4. Hemolytic and Cytotoxicity Assay

When a drug possesses antimicrobial potential, its toxicity is a critical consideration. The hemolysis assay serves as a key safety assessment to evaluate whether a compound induces erythrocyte rupture, which may pose fatal risks. None of the tested compounds exhibited significant hemolytic activity over a range of concentrations (Figure 2B). The hemolysis rates for all compounds remained at approximately 2%.

Cytotoxicity assays can help evaluate the safety of prepared samples as biomaterials and their suitability for contact with human tissues. The experimental results are shown in Figure 2C. As observed, the cytotoxicity of the three tested compounds exhibited a concentration-dependent manner. At a concentration of 12.5 μg/mL, compound **3c** did not significantly affect cell viability (98%). However, when the concentration was increased to 25 μg/mL, viability declined sharply to 30%, which is 16 times the MIC. This steep decline between two adjacent concentrations suggests that the toxicity of compound **3c** may exhibit a clear effect threshold. For compound **4e**, at 200 μg/mL, cell viability was 66%, approximately 32 times the MIC, while for compound **5b**, at the same concentration of 200 μg/mL, viability dropped to 30%, corresponding to 16 times the MIC. The experimental results indicate that all three tested compounds possess a favorable safety index (SI > 10). At concentrations equivalent to their respective MICs, they do not induce cytotoxicity in host cells, demonstrating good biosafety profiles.

### 2.5. Antibacterial Mechanism of 3c, 4e, and 5b

#### 2.5.1. Scanning Electron Microscopic Observation of Bacterial Morphology

**Figure 3:**
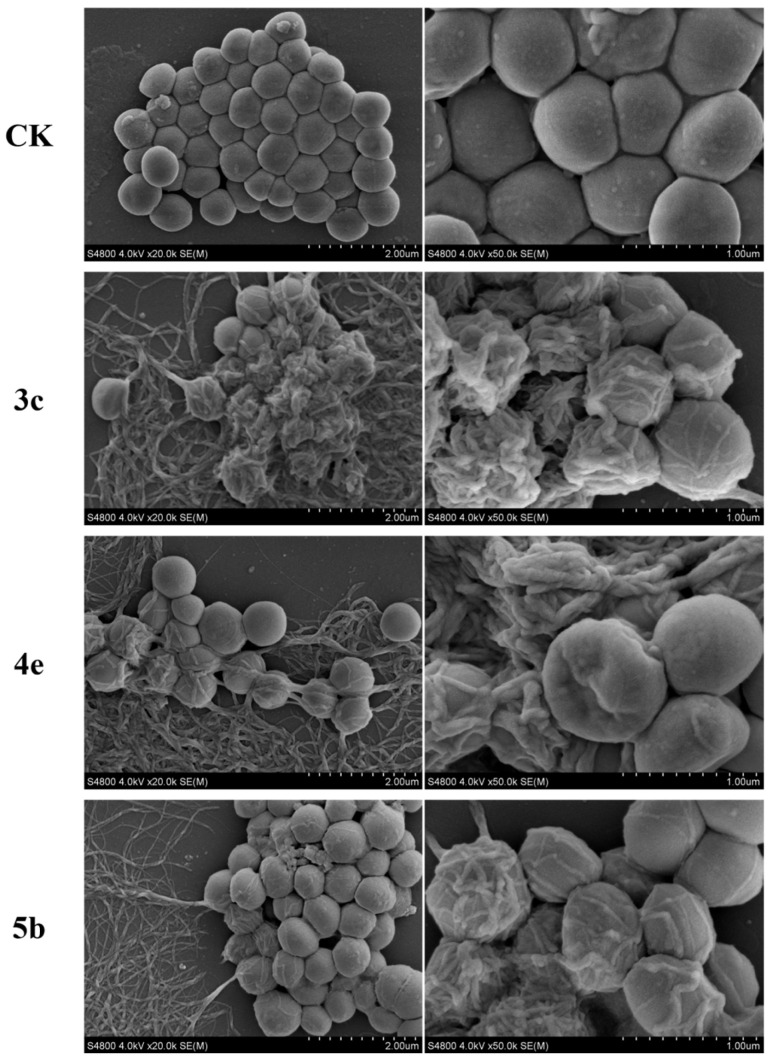
SEM analysis of MRSA ATCC43300 treated with derivatives. Bacteria in mid-logarithmic growth phases were treated with compounds at 4 × MIC for 2 h.

Scanning electron microscopy (SEM) was used to observe morphological differences in the cell walls and membranes of MRSA ATCC43300 treated with the tested compounds. As shown in Figure 3, untreated MRSA exhibited intact cell membranes with a smooth and flat surface (CK). In contrast, when MRSA was treated with compound **3c**, **4e**, or **5b** at 4 × MIC, the cell surfaces appeared wrinkled, uneven, and ruptured. Among these, cells treated with compound **3c** showed the most severe shrinkage and rupture. These results confirm that the tested compounds can disrupt the integrity of cell walls and membranes of MRSA.

#### 2.5.2. Membrane Permeabilization Analysis

**Figure 4:**
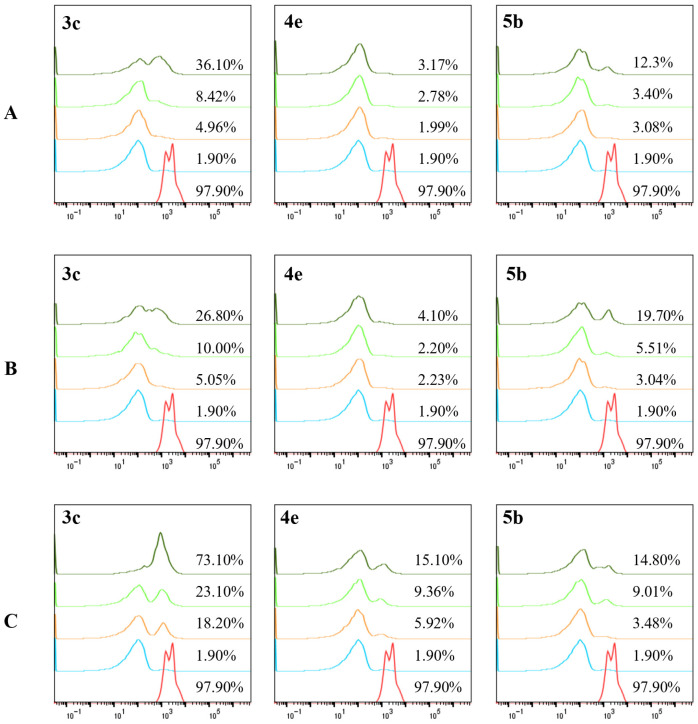
Flow cytometric analysis of PI staining in MRSA ATCC43300 treated with 1 ×, 2 ×, and 4 × MIC derivatives for 5 min (**A**), 30 min (**B**), and 120 min (**C**), respectively. The solid red line represents the positive control, i.e., heat-killed bacterial cells. The solid blue line represents the negative control, i.e., bacterial cells without any drug treatment. The solid orange line represents bacterial cells treated with the drug at 1 × MIC. The solid light-green line represents treatment at 2 × MIC; and the solid dark-green line represents treatment at 4 × MIC.

Propidium iodide (PI) is a membrane-impermeable fluorescent dye that selectively enters bacterial cells with compromised membranes, binds to DNA, and emits fluorescence, thereby serving as an indicator of membrane damage [52,53,54]. Figure 4 shows representative samples analyzed by flow cytometry to measure PI uptake. When MRSA ATCC43300 was exposed to 95 °C for 10 min in the presence of PI, 97.90% of cells were PI-positive (red line). Bacterial cells without any drug treatment showed only 1.90% PI positivity (blue line). As shown in Figure 4, derivative **3c** exhibits significant time and concentration dependence. At the same time point, with the increase in drug concentration, the proportion of PI-positive bacteria doubled. When the incubation time reached 120 min at 4 × MIC, the percentage of PI-positive bacteria was as high as 73.10%, demonstrating that compound **3c** can effectively disrupt the bacterial membrane. Compound **4e** does not exhibit significant membrane penetration ability after a short incubation period. It only demonstrates good membrane penetration when incubated at high concentrations for a long time. As shown in the figure, after 120 min, it exhibits significant membrane penetration compared to the previous time periods. The proportion of PI-positive bacteria at 4 × MIC was 15.10%. Compared with **4e**, **5b** exhibits better membrane permeability, achieving a penetration rate of 12.30% at a high concentration within 5 min. Figure S3 offers a clearer visualization of the bacterial count detected via fluorescence within a given population. The significant reduction in bacteria observed with compound **5b** suggests that it may directly lyse bacterial cells, leading to a decrease in the number of bacteria recorded by the flow cytometer.

#### 2.5.3. Interaction between Compounds and DNA

**Figure 5:**
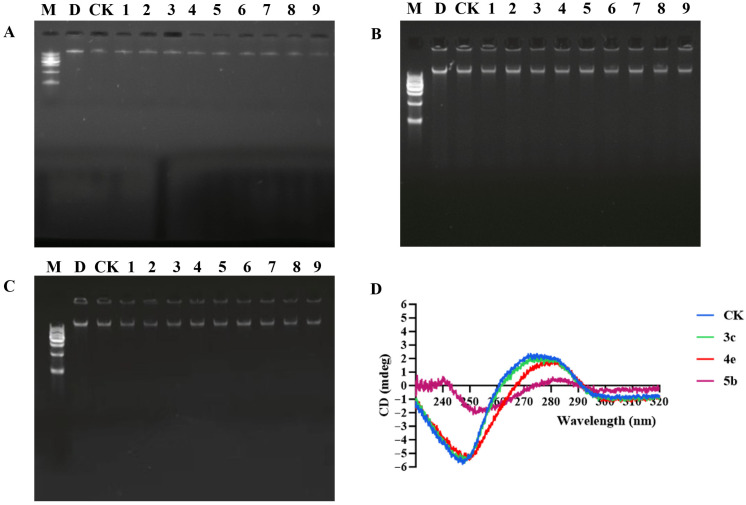
β-Carboline derivatives bind to MRSA ATCC43300 genomic DNA. (**A**) **3c**; (**B**) **4e**; (**C**) **5b**. Gel retardation analysis of derivatives binding to MRSA genomic DNA. M: DNA marker. D: MRSA genomic DNA. CK: 1% DMSO. 1−9: the concentrations of compounds were 200, 100, 50, 25, 12.5, 6.25, 3.125, 1.56, and 0.78 μg/mL, respectively. (**D**) CD spectra of MRSA genomic DNA in the presence of β-carboline derivatives. The concentrations of compounds and DNA were 200 μg/mL and 150 μg/mL, respectively.

To elucidate the mechanism of action, DNA-binding features of the tested compounds were investigated by a gel delay assay. The compounds exhibited no detectable binding to bacterial DNA across the concentration range of 0.78–200 μg/mL (Figure 5A–C). To further clarify the interaction modes between the three β-carboline derivatives and MRSA DNA, we supplemented with circular dichroism (CD) spectroscopy analysis. The untreated control group (CK) displayed a typical B-form DNA conformation, with a positive peak at 274.7 nm and a negative peak at 247.3 nm [55,56,57]. The tested compounds exhibited distinct modes of interaction with DNA (Figure 5D). In the presence of compound **3c**, the positive peak exhibited only a 0.1 nm red shift to 274.8 nm, with high retention of peak intensity (91%). These minimal changes indicate that compound **3c** has no significant specific interaction with MRSA DNA and does not disrupt its secondary structure. For compound **4e**, the positive peak red-shifted by 5.9 nm to 280.6 nm, with moderate retention of peak intensity (80.1%). This spectral shift and moderate intensity loss suggest that **4e** binds moderately to DNA, likely through groove binding or weak intercalation, causing local conformational perturbations without fully compromising double-helix stability [58]. In contrast, **5b** dramatically induced peak intensity loss (74% reduction), although the positive peak red-shifted by an identical distance (5.9 nm). This severe intensity loss, nearly quenching the positive peak, implies that compound **5b** strongly interacts with DNA, possibly via robust intercalation or multi-site binding, which significantly disrupts the base stacking and double-helix structure, leading to extensive DNA strand separation or melting. Collectively, these data reveal a distinct hierarchy in DNA-binding affinity (**5b** > **4e** >> **3c**), which correlates positively with the degree of cationization and π-electron delocalization in these β-carboline derivatives.

### 2.6. Ability of Compounds against MRSA Biofilms 

**Figure 6:**
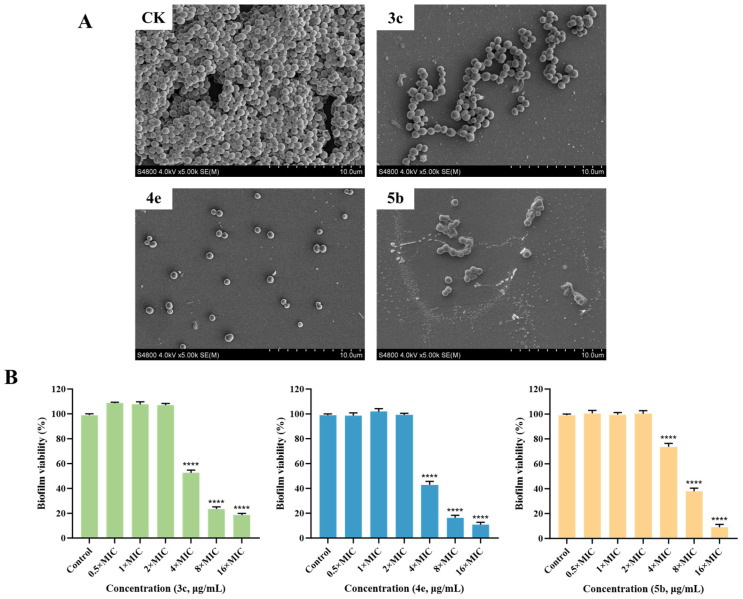
The derivatives destroy bacterial biofilm. (**A**) Scanning electron microscopy observation of MRSA ATCC43300 biofilm. CK: untreated biofilm. (**B**) Inhibitory effects of **3c** (green), **4e** (blue), and **5b** (orange) on biofilm formation. **** Significantly different from control group (*p* < 0.0001). The results are given as means ± SD (*n* = 3).

Bacterial biofilms are a major factor contributing to the persistence of many bacterial infections, conferring increased tolerance to various antimicrobial agents [59]. Thus, it is imperative to develop novel antimicrobial agents capable of inhibiting biofilm formation and killing planktonic bacteria. SEM images revealed distinguishable inhibition effects of the compounds on biofilm formation (Figure 6A). Furthermore, a crystal violet assay was performed to evaluate the potency of compounds **3c**, **4e**, and **5b** at different concentrations in inhibiting the biofilm formation of MRSA ATCC43300. As shown in Figure 6B, the compounds inhibited biofilm formation in a concentration-dependent manner. When MRSA was co-incubated with the compounds at 4 × MIC, the inhibition rates on biofilm formation were approximately 52% for compound **3c**, 73% for compound **4e**, and 75% for compound **5b**. These results are in accordance with those of visual images and further confirm the ability of these compounds to suppress biofilm formation, demonstrating that the compounds exhibit effective activity in inhibiting MRSA biofilm formation.

## 3. Materials and Methods

### 3.1. Chemistry Synthesis—General Methods

All chemicals and solvents were commercially available and used without further purification. Nuclear magnetic resonance spectra of ^1^H NMR (600 MHz) and ^13^C NMR (151 MHz) were recorded on a Bruker Ascend 600 MHz spectrometer in DMSO-*d*_6_ (Billerica, MA, USA). High resolution spectra (HRMS) were measured on a Thermo Scientific Q Exactive Plus spectrometer (Thermo Fisher, Walthman, MA, USA) using electrospray ionization (ESI) in positive mode. The purity of compounds was determined on a reverse-phase high performance liquid chromatograph (RP-HPLC) (P1201, ELITE, Hefei, China) using Ultimate Plus C18 column (5 μm, 4.6 mm × 250 mm, Welch, Jinhua City, China), eluting at a flow rate of 1 mL/min and monitoring at wavelength of 280 nm. All target compounds exhibited purities greater than 93%.

### 3.2. Synthesis of Compounds

#### 3.2.1. General Experimental Procedure for the Synthesis of 5-methyl-β-carboline-1-bromide (**1**)

β-carboline **1** was synthesized as per previous studies [34,37].

#### 3.2.2. (2-Bromophenyl)(6-methyl-9H-pyrido [3,4-b]indol-1-yl)methanone (**2**)

N-bromosuccinimide (NBS, 2.2 mmol) and benzoyl peroxide (BPO, 0.16 mmol) were successively placed in a two-necked round-bottomed flask equipped with a magnetic stir bar. A solution of 5-methyl-β-carboline-1-bromide (**1**, 1.1 mmol) in carbon tetrachloride (40 mL) was transferred into the reaction flask and refluxed at 80 °C. The reaction was monitored by thin-layer chromatography (TLC) until starting material **1** was completely consumed, at which point the reaction was stopped. After cooling to room temperature, the reaction mixture was filtered, and the filter cake was washed with a small amount of dichloromethane. The combined filtrate and washings were concentrated under reduced pressure using a rotary evaporator. The residue was subjected to a preliminary purification by liquid–liquid extraction with dichloromethane and water. The organic layer was separated, dried over anhydrous sodium sulfate, and concentrated to afford the crude product. This crude material was used directly in the subsequent step without further purification. HRMS (ESI) *m*/*z*: calculated for C_19_H_12_Br_2_N_2_O [M+H]^+^ 444.9369; found: 444.9365.

#### 3.2.3. General Procedure for the Synthesis of Compounds **3a–c**

A mixture of cesium carbonate (0.55 mmol) and various aliphatic amines (1.1 mmol) in anhydrous N, N-dimethylformamide (DMF, 2 mL) was placed in a single-necked flask. A solution of compound **2** (0.5 mmol) in anhydrous DMF (2 mL) was added to this stirred mixture. The reaction was allowed to proceed at room temperature and was monitored by TLC until the starting material **2** was completely consumed. Then, the reaction mixture was slowly poured into an ice–water mixture (40 mL), in which a yellow precipitate developed immediately. The resulting yellow precipitate was collected by vacuum filtration, washed thoroughly with water, and dried to afford the crude product. The crude product was chromatographed on silica gel (CH_2_Cl_2_/CH_3_OH, 30:1 *v*/*v*) to give compounds **3a–c**.

##### (2-Bromophenyl)(6-((butylamino)methyl)-9H-pyrido[3,4-b]indol-1-yl)methanone (**3a**)

Pale yellow solid; yield: 40%; m.p.: 234–235 °C; ^1^H NMR (600 MHz, DMSO-*d*_6_) δ 12.2994 (s, 1H), 8.4619 (d, *J* = 4.864 Hz, 1H), 8.4282–8.3945 (m, 2H), 7.8426 (d, *J* = 8.386 Hz, 1H), 7.7629–7.7174 (m, 2H), 7.5803 (d, *J* = 7.449 Hz, 1H), 7.5374 (t, *J* = 7.436 Hz, 1H), 7.4769 (t, *J* = 7.733 Hz, 1H), 4.1735 (s, 2H), 2.8123 (t, *J* = 7.771 Hz, 2H), 1.5802 (p, *J* = 7.619 Hz, 2H), 1.3279 (dq, *J* = 14.875, 7.075 Hz, 3H), 0.8777 (t, *J* = 7.376 Hz, 3H). ^13^C NMR (151 MHz, DMSO-*d*_6_) δ 196.9290, 141.8021, 141.3097, 138.1728, 135.6526, 135.3538, 132.4143, 131.2886, 131.1021, 130.8623, 129.5231, 128.7416, 127.3178, 123.1759, 119.9536, 119.6764, 118.9916, 113.1750, 51.2791, 46.8941, 29.0777, 19.6261, 13.7335. HRMS (ESI) *m*/*z*: calculated for C_23_H_22_BrN_3_O [M+H]^+^ 436.1019; found: 436.1008.

##### (2-Bromophenyl)(6-((hexylamino)methyl)-9H-pyrido[3,4-b]indol-1-yl)methanone (**3b**)

Yellow solid; yield: 36%; m.p.: 233–234 °C; ^1^H NMR (600 MHz, DMSO-*d*_6_) δ 12.3609 (s, 1H), 8.5017–8.4532 (m, 2H), 8.4234 (d, *J* = 4.824 Hz, 1H), 7.8770 (d, *J* = 8.397 Hz, 1H), 7.7974 (d, *J* = 8.455 Hz, 1H), 7.7332 (d, *J* = 8.043 Hz, 1H), 7.5846 (d, *J* = 7.510 Hz, 1H), 7.5402 (t, *J* = 7.464 Hz, 1H), 7.4791 (t, *J* = 7.742 Hz, 1H), 4.3072 (s, 2H), 2.9309 (t, *J* = 7.952 Hz, 2H), 1.6420 (p, *J* = 7.632 Hz, 2H), 1.2844 (dt, *J* = 24.824, 6.342 Hz, 7H), 0.8522 (t, *J* = 6.753 Hz, 3H). ^13^C NMR (151 MHz, DMSO-*d*_6_) δ 196.9205, 142.0861, 141.2451, 138.3198, 135.6710, 135.4582, 132.4285, 131.3313, 131.2269, 131.0136, 129.5447, 127.3370, 124.4694, 123.9916, 119.9935, 119.6894, 118.9935, 113.3327, 50.5597, 46.6294, 30.7913, 25.7673, 25.5837, 21.9520, 13.9257. HRMS (ESI) *m*/*z*: calculated for C_25_H_26_BrN_3_O [M+H]^+^ 464.1333; found: 464.1333.

##### (2-Bromophenyl)(6-((octylamino)methyl)-9H-pyrido[3,4-b]indol-1-yl)methanone (**3c**)

Yellow solid; yield: 30%; m.p.: 226–227 °C; ^1^H NMR (600 MHz, DMSO-*d*_6_) δ 12.2639 (s, 1H), 8.4486 (d, *J* = 4.901 Hz, 1H), 8.4149 (d, *J* = 4.858 Hz, 1H), 8.3601 (s, 1H), 7.8228 (d, *J* = 8.548 Hz, 1H), 7.7184 (dd, *J* = 12.972, 8.337 Hz, 2H), 7.5750 (d, *J* = 7.557 Hz, 1H), 7.5346 (t, *J* = 7.544 Hz, 1H), 7.4732 (t, *J* = 7.822 Hz, 1H), 4.0926 (s, 2H), 2.7291 (t, *J* = 7.712 Hz, 2H), 1.5438 (p, *J* = 7.537 Hz, 2H), 1.2532 (dt, *J* = 33.500, 7.724 Hz, 11H), 0.8276 (t, *J* = 6.958 Hz, 3H). ^13^C NMR (151 MHz, DMSO-*d*_6_) δ 196.9337, 172.3101, 141.6265, 141.3525, 138.0777, 135.6517, 135.2853, 132.4124, 131.2667, 131.1661, 130.6169, 129.5123, 127.3111, 122.6465, 119.9404, 119.6684, 118.9970, 113.0754, 51.7789, 47.5171, 31.2789, 28.7735, 28.6466, 27.3540, 26.4749, 22.1527, 14.0272. HRMS (ESI) *m*/*z*: calculated for C_27_H_30_BrN_3_O [M+H]^+^ 492.1646; found: 492.1646.

#### 3.2.4. General Procedure for the Synthesis of Compounds **4a–e**

Compound **2** (0.55 mmol), different imidazole (0.6 mmol), and acetonitrile (4 mL) were added to a flask. The mixture was refluxed and monitored by TLC until the starting material **2** was completely consumed. Afterwards, the solvent was removed. The residue was then purified by silica gel (CH_2_Cl_2_/CH_3_OH, 10:1 *v*/*v*) to afford compounds **4a–e**.

##### 3-((1-(2-Bromobenzoyl)-9H-pyrido[3,4-b]indol-6-yl)methyl)-1-methyl-1H-imidazol-3-ium bromide (**4a**)

Yellow solid; yield: 36%; m.p.: 192–193 °C; ^1^H NMR (600 MHz, DMSO-*d*_6_) δ 12.3442 (s, 1H), 9.2770 (d, *J* = 7.572 Hz, 1H), 8.4929–8.4491 (m, 2H), 8.4289 (d, *J* = 4.882 Hz, 1H), 7.8715 (d, *J* = 9.422 Hz, 2H), 7.7314 (q, *J* = 4.744 Hz, 3H), 7.5807 (d, *J* = 7.452 Hz, 1H), 7.5378 (t, *J* = 7.433 Hz, 1H), 7.4752 (t, *J* = 7.728 Hz, 1H), 5.6062 (s, 2H), 3.8623 (s, 3H). ^13^C NMR (151 MHz, DMSO-*d*_6_) δ 196.9284, 142.0200, 141.2400, 138.2670, 136.6051, 135.6735, 135.5030, 132.4292, 131.3489, 131.0135, 129.8870, 129.5309, 127.3529, 126.9101, 124.0603, 122.6792, 122.3470, 120.2599, 119.8426, 118.9955, 113.7768, 52.3123, 35.9641. HRMS (ESI) *m*/*z*: calculated for C_23_H_18_Br_2_N_4_O [M-Br]^+^ 445.0659; found: 445.0657.

##### 3-((1-(2-Bromobenzoyl)-9H-pyrido[3,4-b]indol-6-yl)methyl)-1-ethyl-1H-imidazol-3-ium bromide (**4b**)

Yellow solid; yield: 52%; m.p.: 220–221 °C; ^1^H NMR (600 MHz, DMSO-*d*_6_) δ 12.3463 (s, 1H), 9.3835 (d, *J* = 4.541 Hz, 1H), 8.4756 (d, *J* = 3.542 Hz, 2H), 8.4330 (d, *J* = 4.905 Hz, 1H), 7.8958–7.8326 (m, 3H), 7.7348 (t, *J* = 8.265 Hz, 2H), 7.5820 (d, *J* = 7.539 Hz, 1H), 7.5382 (t, *J* = 7.448 Hz, 1H), 7.4756 (t, *J* = 7.720 Hz, 1H), 5.6055 (s, 2H), 4.2129 (q, *J* = 7.312 Hz, 2H), 1.4284 (t, *J* = 7.266 Hz, 3H). ^13^C NMR (151 MHz, DMSO-*d*_6_) δ 196.9209, 142.0228, 141.2368, 138.2584, 135.7876, 135.6667, 135.4969, 132.4192, 131.3388, 131.0099, 129.8951, 129.5233, 127.3433, 126.8591, 122.7271, 122.5599, 122.4875, 120.2510, 119.8451, 118.9896, 113.7917, 52.3782, 44.4193, 15.0919. HRMS (ESI) *m*/*z*: calculated for C_24_H_20_Br_2_N_4_O [M-Br]^+^ 459.0815; found: 459.0810.

##### 3-((1-(2-Bromobenzoyl)-9H-pyrido[3,4-b]indol-6-yl)methyl)-1-propyl-1H-imidazol-3-ium bromide (**4c**)

Yellow solid; yield: 55%; m.p.: 209–210 °C; ^1^H NMR (600 MHz, DMSO-*d*_6_) δ 12.3509 (s, 1H), 9.3836 (d, *J* = 6.612 Hz, 1H), 8.4760 (d, *J* = 4.608 Hz, 2H), 8.4293 (d, *J* = 4.882 Hz, 1H), 7.9196–7.8554 (m, 2H), 7.8341 (s, 1H), 7.7291 (d, *J* = 8.420 Hz, 2H), 7.5820 (d, *J* = 5.771 Hz, 1H), 7.5384 (t, *J* = 7.679 Hz, 1H), 7.5027–7.4499 (m, 1H), 5.6163 (s, 2H), 4.1477 (t, *J* = 7.131 Hz, 2H), 1.8125 (h, *J* = 7.278 Hz, 2H), 0.8447 (t, *J* = 7.331 Hz, 3H). ^13^C NMR (151 MHz, DMSO-*d*_6_) δ 196.9287, 142.0237, 141.2412, 138.2721, 136.0894, 135.6669, 135.5074, 132.4257, 131.3475, 131.0098, 129.8376, 129.5268, 127.3533, 126.8776, 122.8594, 122.6997, 122.5851, 120.2574, 119.8370, 118.9944, 113.8230, 52.4174, 50.5264, 22.8732, 10.5206. HRMS (ESI) *m*/*z*: calculated for C_25_H_22_Br_2_N_4_O [M-Br]^+^ 473.0972; found: 473.0971.

##### 3-((1-(2-Bromobenzoyl)-9H-pyrido[3,4-b]indol-6-yl)methyl)-1-butyl-1H-imidazol-3-ium bromide (**4d**)

Yellow solid; yield: 60%; m.p.: 203–204 °C; ^1^H NMR (600 MHz, DMSO-*d*_6_) δ 12.3499 (s, 1H), 9.4563 (s, 1H), 8.4721 (d, *J* = 5.854 Hz, 2H), 8.4266 (d, *J* = 4.712 Hz, 1H), 7.8997 (s, 1H), 7.8718 (d, *J* = 8.432 Hz, 1H), 7.8372 (s, 1H), 7.7319 (t, *J* = 7.589 Hz, 2H), 7.5780 (d, *J* = 7.437 Hz, 1H), 7.5364 (t, *J* = 7.397 Hz, 1H), 7.4747 (t, *J* = 7.783 Hz, 1H), 5.6149 (s, 2H), 4.1815 (t, *J* = 7.297 Hz, 2H), 1.7800 (q, *J* = 7.410 Hz, 2H), 1.2491 (q, *J* = 7.477 Hz, 2H), 0.8840 (t, *J* = 7.095 Hz, 3H). ^13^C NMR (151 MHz, DMSO-*d*_6_) δ 196.9289, 142.0311, 141.2555, 138.2697, 136.1455, 135.6745, 135.5119, 132.4327, 131.3532, 131.0262, 129.8769, 129.5342, 127.3609, 126.9366, 122.8523, 122.7303, 122.5637, 120.2606, 119.8425, 119.0013, 113.8161, 52.3983, 48.7717, 31.3758, 18.9114, 13.3778. HRMS (ESI) *m*/*z*: calculated for C_26_H_24_Br_2_N_4_O [M-Br]^+^ 487.1128; found: 487.1118.

##### 3-((1-(2-Bromobenzoyl)-9H-pyrido[3,4-b]indol-6-yl)methyl)-1-hexyl-1H-imidazol-3-ium bromide (**4e**)

Yellow oil; yield: 47%; ^1^H NMR (600 MHz, DMSO-*d*_6_) δ 12.3470 (s, 1H), 8.5130 (q, *J* = 4.250, 3.183 Hz, 1H), 8.4759 (d, *J* = 4.942 Hz, 1H), 8.4221 (d, *J* = 4.921 Hz, 1H), 8.0879 (s, 1H), 7.8825 (d, *J* = 4.750 Hz, 1H), 7.8643 (s, 1H), 7.8311 (s, 1H), 7.7330 (dd, *J* = 8.138, 5.038 Hz, 2H), 7.613–7.5640 (m, 1H), 7.5401 (t, *J* = 7.721 Hz, 1H), 7.4845 (dd, *J* = 7.728, 1.806 Hz, 1H), 5.6069 (d, *J* = 3.188 Hz, 2H), 4.1709 (t, *J* = 7.168 Hz, 2H), 1.7879 (q, *J* = 7.124 Hz, 2H), 1.2623 (d, *J* = 4.238 Hz, 4H), 1.2295 (s, 2H), 0.8240 (t, *J* = 13.222 Hz, 3H).^13^C NMR (151 MHz, DMSO-*d*_6_) δ 196.9326, 142.0311, 141.2417, 138.2683, 137.3961, 136.0595, 135.6741, 132.4338, 131.3579, 131.0105, 129.8259, 129.5304, 127.3619, 126.9237, 124.0167, 122.8500, 122.5908, 120.2671, 119.8200, 118.9958, 113.8169, 52.4308, 49.0397, 30.5534, 29.3053, 25.2218, 21.9583, 13.8724. HRMS (ESI) *m*/*z*: calculated for C_28_H_28_Br_2_N_4_O [M-Br]^+^ 515.1441; found: 515,1440.

#### 3.2.5. Synthesis of 1-((1-(2-Bromobenzoyl)-9H-pyrido[3,4-b]indol-6-yl)methyl)pyridinium bromide (**5a**)

Compound **2** (0.55 mmol), pyridine (0.55 mmol) and acetonitrile (4 mL) were added to a flask. The mixture was refluxed and monitored by TLC until the starting material **2** was completely consumed. Afterwards, the solvent was removed. The residue was then purified by silica gel (CH_2_Cl_2_/CH_3_OH, 10:1 *v*/*v*) to afford compound **5a**. Yellow solid; yield: 65%; m.p.: 249–250 °C; ^1^H NMR (600 MHz, DMSO-*d*_6_) δ 12.3773 (s, 1H), 9.3023 (d, *J* = 6.045 Hz, 2H), 8.6171 (d, *J* = 9.444 Hz, 2H), 8.4802 (d, *J* = 4.867 Hz, 1H), 8.4229 (d, *J* = 4.848 Hz, 1H), 8.1875 (t, *J* = 7.005 Hz, 2H), 7.8857 (d, *J* = 8.396 Hz, 1H), 7.8435 (d, *J* = 8.556 Hz, 1H), 7.7257 (d, *J* = 8.036 Hz, 1H), 7.5779 (d, *J* = 7.419 Hz, 1H), 7.5365 (t, *J* = 7.400 Hz, 1H), 7.4758 (t, *J* = 7.710 Hz, 1H), 6.0679 (s, 2H). ^13^C NMR (151 MHz, DMSO-*d*_6_) δ 196.9281, 145.9941, 144.8149, 142.3095, 141.2145, 138.3960, 135.6924, 135.6053, 132.4466, 131.3921, 130.9923, 130.1833, 129.5457, 128.5478, 127.3797, 126.3285, 123.4490, 120.3931, 119.8965, 119.0041, 114.0238, 63.7875. HRMS (ESI) *m*/*z*: calculated for C_24_H_17_Br_2_N_3_O [M-Br]^+^ 442.0550; found: 442.0542.

#### 3.2.6. Synthesis of 1-((1-(2-Bromobenzoyl)-9H-pyrido[3,4-b]indol-6-yl)methyl)quinolinium bromide (**5b**)

Following the procedure of compound **5a**. Yellow solid; yield: 59%; m.p.: 192–193 °C; ^1^H NMR (600 MHz, DMSO-*d*_6_) δ 12.3346 (s, 1H), 9.8158 (d, *J* = 5.183 Hz, 1H), 9.3958 (d, *J* = 8.296 Hz, 1H), 8.6691 (d, *J* = 8.958 Hz, 1H), 8.5183 (d, *J* = 8.229 Hz, 1H), 8.4762 (s, 1H), 8.4503–8.3937 (m, 2H), 8.3245 (d, *J* = 7.237 Hz, 1H), 8.2139 (t, *J* = 8.055 Hz, 1H), 8.0149 (t, *J* = 7.679 Hz, 1H), 7.8612 (d, *J* = 8.527 Hz, 1H), 7.7676 (d, *J* = 8.599 Hz, 1H), 7.7130 (d, *J* = 7.944 Hz, 1H), 7.5645 (d, *J* = 7.443 Hz, 1H), 7.5256 (t, *J* = 7.381 Hz, 1H), 7.4646 (t, *J* = 7.784 Hz, 1H), 6.5525 (s, 2H). ^13^C NMR (151 MHz, DMSO-*d*_6_) δ 196.8574, 150.2578, 148.0919, 141.9540, 141.1831, 138.2126, 137.6585, 135.7544, 135.6165, 135.4857, 132.4012, 131.3387, 130.9268, 130.8840, 130.0221, 129.5036, 129.0062, 127.3354, 125.8527, 122.6283, 122.0145, 121.9250, 120.3569, 120.0108, 119.4338, 118.9645, 113.8812, 60.4143. HRMS (ESI) *m*/*z*: calculated for C_28_H_19_Br_2_N_3_O [M-Br]^+^ 492.0706; found: 492.0688.

#### 3.2.7. General Procedure for the Synthesis of Compounds **6a–f**

Potassium carbonate (1.1 mmol) and different anilines (0.55 mmol) were added to a solution of compound **2** (0.55 mmol) in DMF (4 mL). The mixture was refluxed at 120 °C and monitored by TLC until the starting material was completely consumed. After completion, the reaction was stopped and allowed to cool to room temperature. The reaction mixture was then poured into approximately 50 mL of an ice–water mixture, upon which a precipitate formed immediately. The solid was collected by suction filtration, washed with water, and dried to afford the crude product. The crude material was dissolved in ethyl acetate and purified by silica gel (PE/EA, 6:1 *v*/*v*) to obtain the product **6a–f**.

##### (2-Bromophenyl)(6-((phenylamino)methyl)-9H-pyrido[3,4-b]indol-1-yl)methanone (**6a**)

Yellow solid; yield: 60%; m.p.: 185–187 °C; ^1^H NMR (600 MHz, DMSO-*d*_6_) δ 12.1735 (s, 1H), 8.4106 (s, 2H), 8.3080 (d, *J* = 1.952 Hz, 1H), 7.7903 (d, *J* = 8.398 Hz, 1H), 7.7212 (d, *J* = 8.272 Hz, 1H), 7.6569 (d, *J* = 8.484 Hz, 1H), 7.5705 (d, *J* = 7.449 Hz, 1H), 7.5264 (t, *J* = 7.426 Hz, 1H), 7.4637 (t, *J* = 7.653 Hz, 1H), 7.0656–7.0015 (m, 2H), 6.6382 (d, *J* = 7.542 Hz, 2H), 6.5042 (t, *J* = 7.296 Hz, 1H), 6.2779 (t, *J* = 5.897 Hz, 1H), 4.4247 (d, *J* = 5.812 Hz, 2H). ^13^C NMR (151 MHz, DMSO-*d*_6_) δ 196.9505, 148.8062, 141.4437, 141.1591, 137.8594, 135.6220, 135.1431, 132.6311, 132.3824, 131.2034, 129.4870, 129.1299, 128.8999, 127.2887, 120.4475, 120.0151, 119.6978, 119.0041, 115.8520, 113.0531, 112.4521, 46.9560. HRMS (ESI) *m*/*z*: calculated for C_25_H_18_BrN_3_O [M+H]^+^ 456.0706; found: 456.0703.

##### (2-Bromophenyl)(6-((m-tolylamino)methyl)-9H-pyrido[3,4-b]indol-1-yl)methanone (**6b**)

Yellow solid; yield: 65%; m.p.: 169–170 °C; ^1^H NMR (600 MHz, DMSO-*d*_6_) δ 12.1746 (s, 1H), 8.4057 (s, 2H), 8.2939 (s, 1H), 7.7899 (d, *J* = 8.314 Hz, 1H), 7.7189 (d, *J* = 8.055 Hz, 1H), 7.6480 (d, *J* = 8.320 Hz, 1H), 7.5673 (d, *J* = 7.521 Hz, 1H), 7.5245 (t, *J* = 7.445 Hz, 1H), 7.4678 (d, *J* = 7.977 Hz, 1H), 6.9131 (t, *J* = 7.753 Hz, 1H), 6.4727 (s, 1H), 6.4312 (d, *J* = 10.028 Hz, 1H), 6.3298 (d, *J* = 7.404 Hz, 1H), 6.1822 (t, *J* = 5.977 Hz, 1H), 4.4135 (d, *J* = 5.836 Hz, 2H), 2.1398 (s, 3H).^13^C NMR (151 MHz, DMSO-*d*_6_) δ 196.9697, 148.8280, 141.4576, 141.1547, 137.8728, 137.8364, 135.6409, 135.1501, 132.7651, 132.3992, 131.2241, 129.4977, 129.1189, 128.8011, 127.3061, 120.3732, 120.0286, 119.7065, 119.0190, 116.8532, 113.1895, 113.0455, 109.6904, 46.9495, 21.4710. HRMS (ESI) *m*/*z*: calculated for C_26_H_20_BrN_3_O [M+H]^+^ 470.0863; found: 470.0841.

##### (2-Bromophenyl)(6-(((2-methoxyphenyl)amino)methyl)-9H-pyrido[3,4-b]indol-1-yl)methanone (**6c**)

Yellow solid; yield: 58%; m.p.: 175–176 °C; ^1^H NMR (600 MHz, DMSO-*d*_6_) δ 12.1678 (s, 1H), 8.4047 (t, *J* = 1.693 Hz, 2H), 8.2952 (s, 1H), 7.7803 (d, *J* = 8.380 Hz, 1H), 7.7182 (d, *J* = 8.016 Hz, 1H), 7.6529 (dd, *J* = 8.501, 1.695 Hz, 1H), 7.5936–7.5462 (m, 1H), 7.5539–7.4945 (m, 1H), 7.4924–7.4312 (m, 1H), 6.8269–6.7700 (m, 1H), 6.6632 (t, *J* = 7.645 Hz, 1H), 6.5392–6.4815 (m, 2H), 5.5641 (td, *J* = 6.141, 2.413 Hz, 1H), 4.4809 (d, *J* = 5.976 Hz, 2H), 3.8050 (d, *J* = 1.144 Hz, 3H). ^13^C NMR (151 MHz, DMSO-*d*_6_) δ 196.9501, 146.5356, 141.4567, 141.1571, 137.9449, 137.8432, 135.6129, 135.1395, 132.7787, 132.3747, 131.1922, 129.4758, 128.9562, 127.2882, 120.9855, 120.3002, 120.0270, 119.7327, 119.0009, 115.7583, 113.0980, 110.7792, 109.7843, 109.7271, 55.3849, 46.8474. HRMS (ESI) *m*/*z*: calculated for C_26_H_20_BrN_3_O_2_ [M+H]^+^ 486.0812; found: 486.0810.

##### (2-Bromophenyl)(6-(((3-methoxyphenyl)amino)methyl)-9H-pyrido[3,4-b]indol-1-yl)methanone (**6d**)

Yellow solid; yield: 63%; m.p.: 120–121 °C; ^1^H NMR (600 MHz, DMSO-*d*_6_) δ 12.1768 (s, 1H), 8.4109 (s, 2H), 8.3016 (d, *J* = 3.679 Hz, 1H), 7.7906 (d, *J* = 8.376 Hz, 1H), 7.7357–7.7061 (m, 1H), 7.6495 (dd, *J* = 8.482, 1.616 Hz, 1H), 7.5717 (dd, *J* = 7.525, 1.743 Hz, 1H), 7.5513–7.5071 (m, 1H), 7.4875–7.4464 (m, 1H), 6.9339 (t, *J* = 8.084 Hz, 1H), 6.3147 (t, *J* = 5.868 Hz, 1H), 6.2522 (dd, *J* = 8.107, 2.180 Hz, 1H), 6.1849 (t, *J* = 2.297 Hz, 1H), 6.0974 (dd, *J* = 8.000, 2.366 Hz, 1H), 4.4122 (d, *J* = 5.583 Hz, 2H), 3.6198 (s, 3H). ^13^C NMR (151 MHz, DMSO-*d*_6_) δ 196.9540, 160.3104, 150.1463, 141.4470, 141.1564, 137.8668, 135.6273, 135.1507, 132.6205, 132.3857, 131.2050, 129.5998, 129.4871, 129.1088, 127.2919, 120.4182, 120.0138, 119.6904, 119.0092, 113.0525, 105.6516, 101.2663, 98.2768, 54.6308, 46.9845. HRMS (ESI) *m*/*z*: calculated for C_26_H_20_BrN_3_O_2_ [M+H]^+^ 486.0812; found: 486.0812.

##### (2-Bromophenyl)(6-(((4-methoxyphenyl)amino)methyl)-9H-pyrido[3,4-b]indol-1-yl)methanone (**6e**)

Yellow solid; yield: 66%; m.p.: 181–182 °C; ^1^H NMR (600 MHz, DMSO-*d*_6_) δ 12.1660 (s, 1H), 8.4058 (d, *J* = 2.066 Hz, 2H), 8.3034 (d, *J* = 2.431 Hz, 1H), 7.7804 (dd, *J* = 8.358, 2.176 Hz, 1H), 7.7213 (d, *J* = 8.140 Hz, 1H), 7.6523 (d, *J* = 8.391 Hz, 1H), 7.5707 (d, *J* = 7.486 Hz, 1H), 7.5269 (t, *J* = 7.567 Hz, 1H), 7.4838–7.4394 (m, 1H), 6.6809 (d, *J* = 8.911 Hz, 2H), 6.5963 (d, *J* = 8.931 Hz, 2H), 5.8649 (s, 1H), 4.3763 (s, 2H), 3.5950 (s, 3H). ^13^C NMR (151 MHz, DMSO-*d*_6_) δ 196.9621, 150.7962, 143.0635, 141.4544, 141.1366, 137.8552, 135.6237, 135.1361, 132.8960, 132.3894, 131.2298, 131.2120, 129.4895, 129.2596, 129.1917, 127.2962, 120.4749, 120.0040, 119.6865, 119.0109, 114.5908, 114.5391, 113.5352, 112.9975, 55.3139, 47.8060. HRMS (ESI) *m*/*z*: calculated for C_26_H_20_BrN_3_O_2_ [M+H]^+^ 486.0812; found: 486.0812.

##### (2-Bromophenyl)(6-(((3-nitrophenyl)amino)methyl)-9H-pyrido[3,4-b]indol-1-yl)methanone (**6f**)

Yellow solid; yield: 66%; m.p.: 190–191 °C; ^1^H NMR (600 MHz, DMSO-*d*_6_) δ 12.2067 (s, 1H), 8.4434–8.4009 (m, 2H), 8.3305 (s, 1H), 7.8146 (d, *J* = 8.359 Hz, 1H), 7.7211 (d, *J* = 8.031 Hz, 1H), 7.6687 (dd, *J* = 8.480, 1.810 Hz, 1H), 7.5728 (dd, *J* = 7.497, 1.878 Hz, 1H), 7.5274 (t, *J* = 7.446 Hz, 1H), 7.4649 (td, *J* = 7.783, 1.807 Hz, 1H), 7.4271 (t, *J* = 2.288 Hz, 1H), 7.3564–7.2836 (m, 2H), 7.1107–7.0399 (m, 2H), 4.5241 (d, *J* = 5.685 Hz, 2H). ^13^C NMR (151 MHz, DMSO-*d*_6_) δ 196.9470, 149.7680, 148.8363, 141.4138, 141.2809, 137.9270, 135.6304, 135.2082, 132.3904, 131.3834, 131.2246, 131.1441, 130.0486, 129.4945, 129.0808, 127.2964, 120.5750, 120.1125, 119.7420, 119.0072, 118.6377, 113.2472, 110.0734, 105.7539, 46.6927. HRMS (ESI) *m*/*z*: calculated for C_25_H_17_BrN_4_O_3_ [M+H]^+^ 501.0557; found: 501.0556.

### 3.3. Minimum Inhibitory Concentration (MIC) Assay

The MIC was determined according to Clinical and Laboratory Standards Institute (CLSI) guidelines using a microbroth dilution method in 96-well microtiter plates. MRSA ATCC43300 and *Bacillus subtilis* ATCC336690 were cultured in Mueller–Hinton broth (MHB), whereas *Escherichia coli* ATCC25922 and *Pseudomonas aeruginosa* ATCC337940 were grown in Luria–Bertani broth (LB). All bacterial strains were incubated at 37 °C to mid-logarithmic phase. The bacterial suspensions were then adjusted to approximately 1 × 10^5^ CFU/mL in the corresponding broth (MHB or LB) and dispensed into the microtiter plates. Test compounds were serially diluted twofold using DMSO and added to the plates. Vancomycin and colistin served as positive controls for Gram-positive and Gram-negative bacteria, respectively, and 1% DMSO was used as the negative control. Following 18 h of incubation at 37 °C, the MIC was defined as the lowest concentration that completely inhibited visible bacterial growth. All experiments were performed in triplicate.

### 3.4. Zeta-Potential Analysis

The surface charge of bacterial cells after compound treatment was determined by zeta-potential analysis using a Zetasizer Nano ZS90 (Malvern, UK) [60]. Overnight cultures of *E. coli* were harvested by centrifugation, washed twice with PBS, and resuspended in the same buffer (OD_600_ = 0.5). The bacterial suspension was incubated with representative compounds at 200 μg/mL for 15 min at 37 °C. Untreated cells served as control. The zeta potential was measured at 25 °C in triplicate, and the results are expressed as means ± SD.

### 3.5. Minimum Bactericidal Concentration (MBC) Assay

Based on the MIC results, the MBC was determined using the agar plate method. From the MIC assay plates incubated the previous day, bacterial suspensions were taken from the well with the lowest concentration showing no visible growth and all wells with higher drug concentrations. The suspensions were mixed thoroughly, and 10 μL from the mixture was evenly spread onto LB agar plates. Each sample was tested in technical triplicates. Vancomycin served as positive control. The plates were incubated at 37 °C for 24 h. The lowest drug concentration at which no single colony was observed by the naked eye was defined as the MBC of the compound.

### 3.6. Time–Killing Curve Assay

MRSA ATCC43300 was cultured in MH broth at 37 °C until the mid-logarithmic phase was reached. The bacterial suspension was then diluted with MH broth to a concentration of 1 × 10^5^ CFU/mL. Several culture tubes were prepared with 5 mL of MH broth, the diluted bacterial suspension, and compounds at different concentrations (1 × MIC, 2 × MIC, 4 × MIC). The tubes were subsequently incubated at 37 °C and 250 rpm. At specified time points from 0 to 24 h, 100 μL samples were withdrawn, serially diluted, and spread onto MH agar plates. After 18 h of incubation, the bactericidal efficiency of the compounds was determined by plate colony counting, with 0.4% DMSO used as the negative control (CK) and vancomycin used as the positive control.

### 3.7. Resistance Development Assay

To evaluate the potential development of resistance in MRSA ATCC43300 to the tested carbolines, a 30-day serial passage experiment was conducted following a previously described method [61]. Briefly, bacterial cultures were grown to mid-log phase and adjusted to approximately 10^5^ CFU/mL. The MIC was determined initially as reported earlier. Each day, an inoculum equivalent to 1/4 × MIC of the bacteria was transferred into fresh MH broth and incubated to mid-log phase for use in the subsequent day’s MIC determination. This process was repeated daily for 30 days, and the MIC values of the carbolines were recorded throughout the period. Vancomycin was included as a positive control in the experiment.

### 3.8. Hemolytic Assay

Hemolytic activity of the compounds was evaluated. Human blood was collected and centrifuged at 1500 rpm for 10 min at 25 °C. The supernatant was washed with phosphate-buffered saline (PBS, pH = 7.4) until colorless. Then, 100 μL of an 8% red blood cell suspension diluted in PBS was mixed with 100 μL of different drug concentrations in a 96-well plate and incubated at 37 °C for 1 h. PBS and 0.1% Triton X-100 were used as negative and positive controls, respectively, with three replicates per group. After incubation, the plate was centrifuged at 1500 rpm for 5 min, and 100 μL of the supernatant was transferred to a new 96-well plate. The absorbance was measured at 540 nm using a full-wavelength microplate reader. Hemolysis (%) = [(OD_sample_− OD_PBS_)/(OD_Triton_ – OD_PBS_)] × 100% [35].

### 3.9. Cytotoxicity Assay

To evaluate the effects of compounds on the viability of murine peritoneal macrophage RAW264.7 cells, a Cell Counting Kit-8 (CCK-8) assay was employed for cytotoxicity detection [61]. The experimental procedure was conducted as follows: RAW264.7 cells in the logarithmic growth phase were seeded into 96-well plates at a density of 2.5 × 10^4^ cells per well and incubated overnight in a humidified atmosphere containing 5% (*v*/*v*) CO_2_ at 37 °C. After the cells were fully attached, they were subjected to experimental treatments. Different concentrations of compounds **3c**, **4e**, and **5b** (with concentration gradients set at 0.78, 1.56, 3.12, 6.25, 12.5, 25, 50, 100, and 200 μg/mL) were added to the corresponding wells, while a solvent control group treated with 1% DMSO was included. All groups were further incubated for 24 h. Following drug treatment, 10 μL of CCK-8 solution, which primarily contains the water-soluble tetrazolium salt WST-8, was added to each well. The plate was then shielded from light and incubated at 37 °C for an additional 4 h. Absorbance was measured at a wavelength of 460 nm using a microplate reader. The relative cell viability at each drug concentration was calculated by comparing the absorbance values between the experimental and control groups, thereby assessing the impact of the compounds on RAW264.7 cell viability.

### 3.10. Scanning Electron Microscope (SEM) Observation of Bacterial Morphology

Bacterial cultures in the mid-log phase (10^8^ CFU/mL) were treated with compounds **3c**, **4e**, and **5b** at a concentration of 4 × MIC for 2 h at 37 °C. Untreated bacteria were used as the negative control. Following treatment, the bacterial cells were harvested by centrifugation and fixed for 15 min at 4 °C using 2.5% (*v*/*v*) glutaraldehyde. The fixed samples were then dehydrated through a graded ethanol series (20%, 50%, 70%, 85%, 95% × 2, and 100% × 2) and subsequently subjected to critical point drying. Prior to imaging, the samples were sputter-coated with a gold–palladium alloy. Morphological alterations of the bacterial cells were examined using a S-4800 (Hitachi, Tokyo, Japan) scanning electron microscope.

### 3.11. Flow Cytometry Assay

PI fluorescent dyes can cross broken cell membranes, so they can be applied to examine the disruptive effects of compounds on bacterial cell membranes. Bacterial cultures at mid-log phase (1 × 10^8^ CFU/mL) were treated with compounds **3c**, **4e**, and **5b** (1 ×, 2 × and 4 × MIC). Bacterial cells without any drug treatment as the negative control. The mixtures were incubated at 37 °C for 5, 30, and 120 min, respectively. After incubation, the bacterial suspensions were stained with PI at a final concentration of 50 µg/mL and further incubated in the dark for 15 min. Finally, flow cytometry (FACS Calibur) was applied to perform fluorescence detection on the samples [62].

### 3.12. Gel Retardation Assay

The genomic DNA of MRSA ATCC43300 was incubated for 10 min at room temperature with different concentrations of tested compounds (0.78, 1.56, 3.125, 6.25, 12.5, 25, 50, 100, and 200 μg/mL), with 1% DMSO as the negative control (CK). The migration of DNA was assessed by electrophoresis on a 1% agarose gel [63].

### 3.13. Circular Dichroism (CD) Spectroscopy

Genome extraction kits were obtained for the extraction of bacterial DNA [64]. Genomic DNA (150 μg/mL) was incubated with the compounds at a final concentration of 200 μg/mL for 10 min. An untreated DNA sample served as the negative control. Measurements were performed using a J-1700 CD spectrometer (JASCO, Tokyo, Japan) with a 2.0 nm path length cuvette. The CD spectrum of each sample was recorded from 220 to 320 nm at a scanning rate of 10 nm/min.

### 3.14. Biofilm Observed by SEM

SEM was used to further explore the role of the tested compounds on biofilm morphology [65]. The MRSA ATCC43300 (10^8^ CFU/mL) was placed in tryptic soy broth (TSB) medium and cultured for 24 h in the presence (4 × MIC) of tested compounds (**3c**, **4e**, and **5b**). Untreated biological membranes were used as the negative control. After incubation, the biofilm was washed twice with PBS. Subsequently, the biofilm was fixed for 15 min with 2.5% glutaraldehyde and then subjected to gradient dehydration with ethanol (20%, 50%, 70%, 85%, 95% × 2, and 100% × 2). Finally, SEM was used to visualize the morphology of sputtered gold–palladium biofilm.

### 3.15. Biofilm Inhibition by Compounds

A crystal violet staining assay was conducted to evaluate the inhibitory effect of the compounds on biofilm formation [66]. MRSA ATCC43300 (10^8^ CFU/mL) was inoculated into a 96-well plate and co-incubated with the test compound solutions at concentrations ranging from 0.5 × to 16 × MIC for 24 h at 37 °C, with 1% DMSO as the negative control. Following incubation, the biofilms were gently washed twice with PBS and fixed with 2.5% glutaraldehyde for 15 min. The fixed biofilms were then stained with 0.1% crystal violet for 30 min, after which the plates were dried overnight at room temperature. After drying, the stained biofilm was solubilized in 95% ethanol for 30 min with gentle shaking. The absorbance of the resulting solution was measured at 570 nm using a microplate reader to quantify the biofilm biomass.

### 3.16. Statistical Analysis

Data were analyzed using one-way ANOVA followed by Dunnett’s multiple-comparison test for statistical significance (*p*-value) in GraphPad Prism 7.0, and plotted as means ± SD. Significance is indicated by **** *p* < 0.0001. All experiments were performed with three independent biological replicates.

## 4. Conclusions

In this study, four categories of neutral or ionic β-carboline derivatives were synthesized via amination with different nitrogen-containing agents. Three of the most active compounds were selected to evaluate their antibacterial activity and explore the underlying mechanisms. Among these, compounds **4e** and **5b** demonstrated bactericidal effects, while **3c** displayed bacteriostatic activity. All three tested compounds showed low cytotoxicity, low hemolytic potential, and favorable resistance profiles. Further investigation into their antibacterial mechanisms suggested that all three compounds possess the ability to disrupt bacterial cell walls and membranes. **4e** binds moderately to DNA and induces local conformational perturbations without fully compromising double-helix stability, while **5b** strongly interacts with DNA and significantly disrupts base stacking and the double-helix structure, leading to extensive DNA strand separation or melting. Enhancing antimicrobial potency through balancing lipophilicity, cationization, and π-electron delocalization may provide valuable insights for the future design of antimicrobial agents. While the present study demonstrates the promising antibacterial activity of C-6 aminated β-carboline derivatives, their pharmacokinetic properties remain to be investigated. Future studies should include in vivo pharmacokinetic profiling and efficacy evaluation to fully assess the therapeutic potential of this class of compounds.

## Figures and Tables

**Table 1:** The minimum inhibitory concentration (MIC) of derivatives (μg/mL)

	MIC (μg/mL)			
	MRSA ATCC43300 ^a^	*B. subtilis* BNCC336690 ^b^	*E. coli* ATCC25922 ^c^	*P. aeruginosa* BNCC337940 ^d^
**3a**	25	50	>200	>200
**3b**	6.25	6.25	>200	>200
**3c**	1.56	3.125	>200	>200
**4a**	50	100	>200	>200
**4b**	50	100	>200	>200
**4c**	25	50	>200	>200
**4d**	12.5	25	>200	>200
**4e**	6.25	12.5	>200	>200
**5a**	100	200	>200	>200
**5b**	12.5	25	>200	>200
**6a**	>200	>200	>200	>200
**6b**	>200	>200	>200	>200
**6c**	>200	>200	>200	>200
**6d**	>200	>200	>200	>200
**6e**	>200	>200	>200	>200
**6f**	>200	>200	>200	>200
Van ^e^	1	0.39	NT	NT
Col ^f^	NT ^g^	NT	1.56	1.56

^a^ MRSA ATCC43300: methicillin-resistant *Staphylococcus aureus* ATCC43300. ^b^ *B. subtilis* BNCC336690: *Bacillus subtilis* BNCC336690. ^c^ *E. coli* ATCC25922: *Escherichia coli* ATCC25922, penicillin-susceptible strain. ^d^ *P. aeruginosa* BNCC337940: *Pseudomonas aeruginosa* BNCC337940. ^e^ Van: vancomycin. ^f^ Col: colistin. ^g^ NT: not tested.

**Table 2:** Minimum bactericidal concentration (MBC) of **3c**, **4e**, and **5b**

Compound	MIC (μg/mL)	MBC (μg/mL)	MBC/MIC
**3c**	1.56	25	16
**4e**	6.25	12.5	2
**5b**	12.5	25	2
Van	0.78	3.125	4

## Data Availability

The original contributions presented in this study are included in the article/Supplementary Materials. Further inquiries can be directed to the corresponding authors.

## Supplementary Materials

The following supporting information can be downloaded at https://www.mdpi.com/article/10.3390/antibiotics15040339/s1. Figure S1: HRMS of compound **2**; Figure S2: Zeta potential of E. coli after treatment with **3c**, **4e**, and **5b**; Figure S3: Flow cytometry pseudo-color image of PI staining in MRSA; Figures S4–S19: ^1^H NMR, ^13^C NMR and HRMS data of compounds **3a–c**, **4a–e**, **5a–b**, and **6a–f;** HPLC analysis of compounds **3a–c**, **4a–e**, **5a–b**, and **6a–f.**
