# Supplementary material for: Synthesis, Antibacterial Activity, and Mechanism of C-6 Aminated β-Carboline Derivatives Against MRSA
Source: Antibiotics (Basel). 2026 Mar 26;15(4):339. doi: 10.3390/antibiotics15040339 (PMC13114192; doi:10.3390/antibiotics15040339)
Supplement: Supplementary file 1 [file antibiotics-15-00339-s001.zip › antibiotics-4183993-supplementary.pdf]

Supplementary Materials

Synthesis, Antibacterial Activity and Mechanism of Action of C-6 Aminated β-Carboline Derivatives against Methicillin-Resistant *Staphylococcus aureus* (MRSA)

Qiuran Wei [1,2], Weida Liang [1], Hongda Qiu [1], Xing Zhao [1], Yang Li [3], Han Ouyang [3], Bowen Han [3], Lingling Zhao [1], Xiao Wang [2,*], Hongze Liang [1,*]

[1] School of Materials Science and Chemical Engineering, Ningbo University, Ningbo 315211, China; wqr1880898@163.com (Q.W.); liangweida94@gmail.com (W.L.); qiuhongda1998@163.com (H.Q); 15167214127@163.com (X.Z.); zhaolingling@nbu.edu.cn (L.Z.)

[2] Health Science Center, Ningbo University, Ningbo 315211, China

[3] Institute of Drug Discovery Technology, Ningbo University, Ningbo 315211, China; liyang@nbu.edu.cn (Y.L.); ouyanghan@nbu.edu.cn (H.O.); hanbowen@nbu.edu.cn (B.H.)

* Correspondence: wangxiao@nbu.edu.cn (X.W.); lianghongze@nbu.edu.cn (H.L.)

Contents

1. HRMS of Compound **2** (Figure S1)

2. Zeta potential of E. coli after treatment with **3c**, **4e**, and **5b** (Figure S2)

3. Flow Cytometry Pseudocolor Image of PI Staining in MRSA (Figure S3)

4. ^{1}H NMR, ^{13}C NMR and HRMS Data of Compounds **3a–c**, **4a–e**, **5a–b**, and **6a–f** (Figure S4-S19)

5. HPLC Analysis of Compounds **3a–c**, **4a–e**, **5a–b**, and **6a–f**

1. HRMS of Compound **2**

Figure S1. HRMS data of **2**

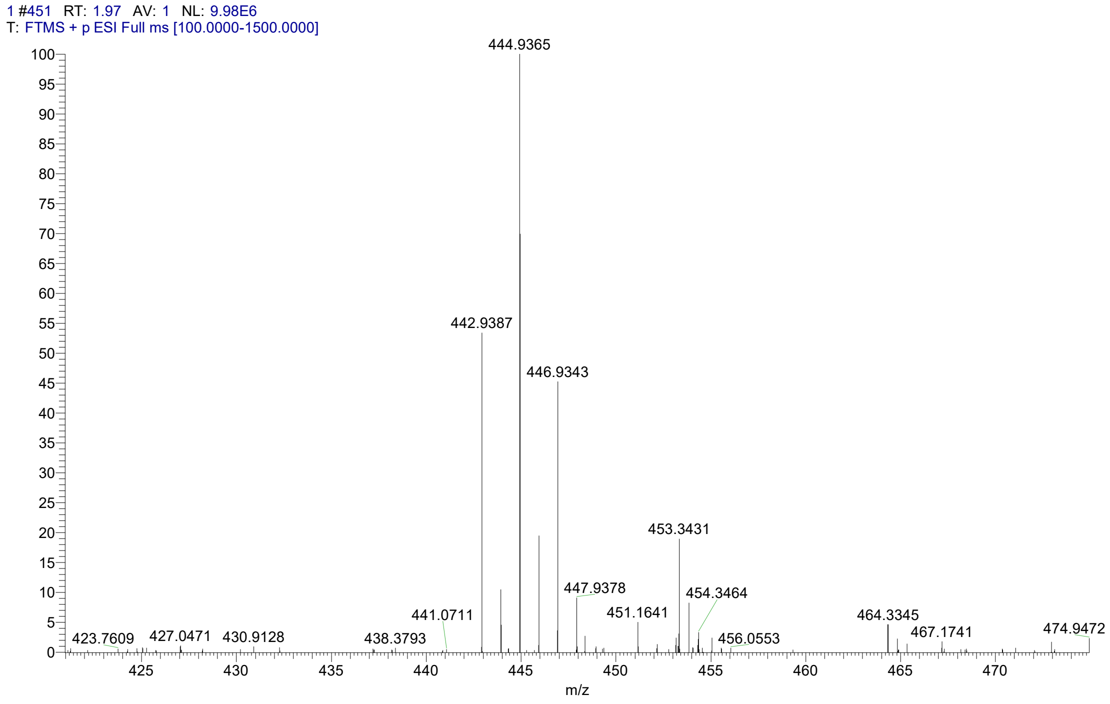

2. Zeta Potential Analysis

Figure S2. Zeta potential of E. coli after treatment with **3c**, **4e**, and **5b**. The results are given as the mean ± SD (n = 3).

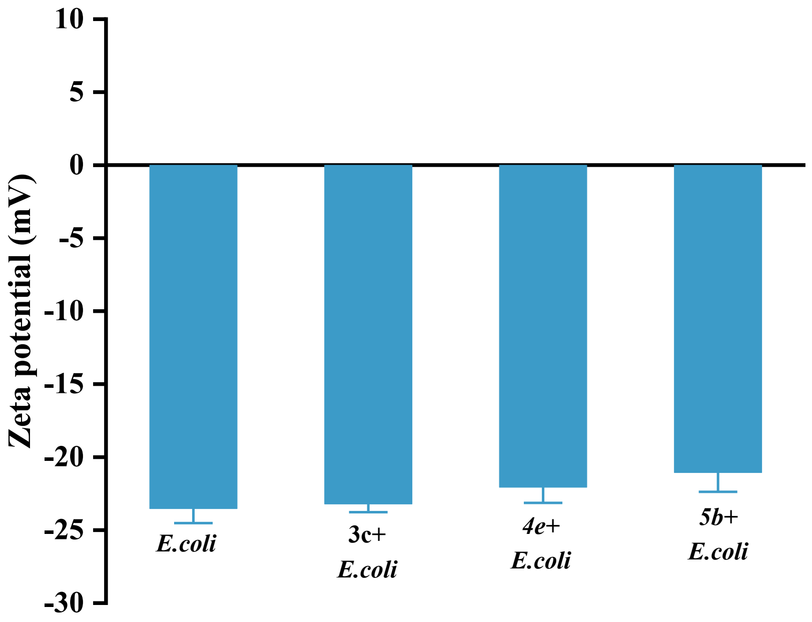

3. Flow Cytometry Pseudocolor Image of PI Staining in MRSA

Figure S3. Flow cytometry pseudocolor image of PI staining in MRSA treated with 1 ×, 2 ×, and 4 × MIC derivatives for 5 min, 30 min, and 120 min, respectively

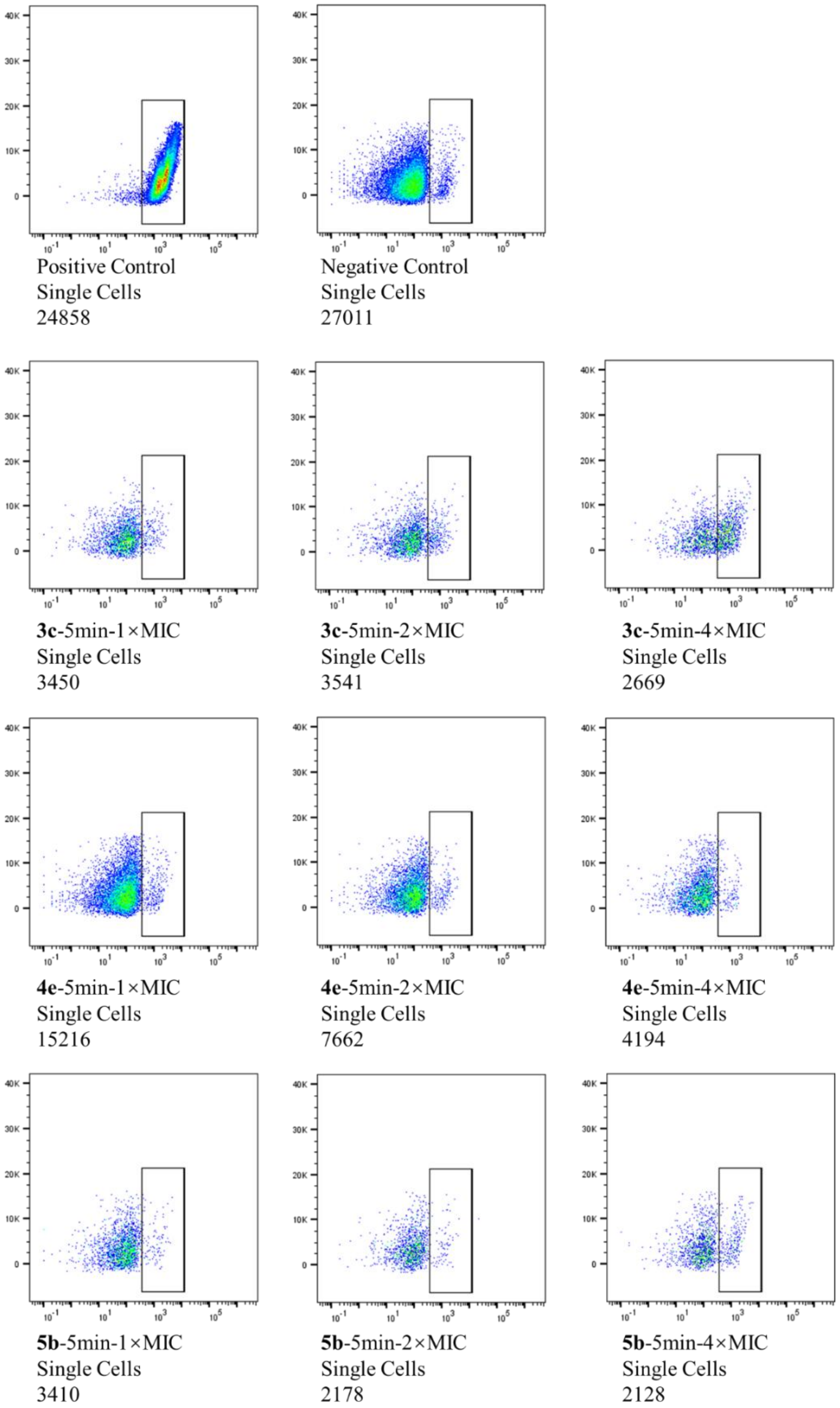

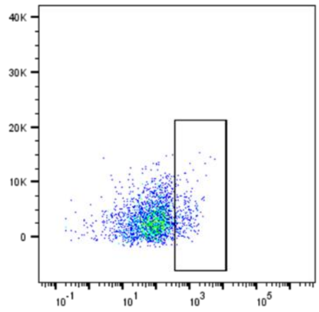

3c-30min-1×MIC
Single Cells
4001

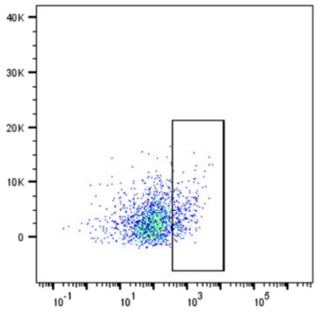

3c-30min-2×MIC
Single Cells
2574

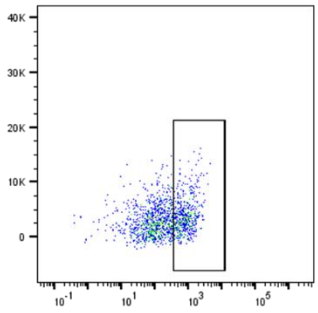

5b-30min-4×MIC
Single Cells
1971

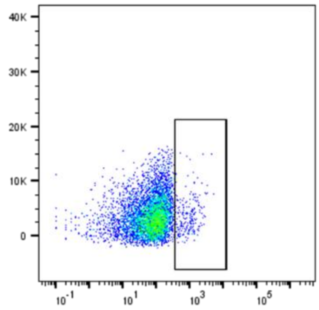

4e-30min-1×MIC
Single Cells
9962

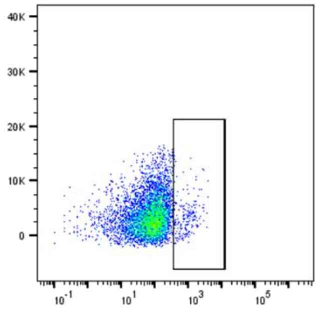

4e-30min-2×MIC
Single Cells
9934

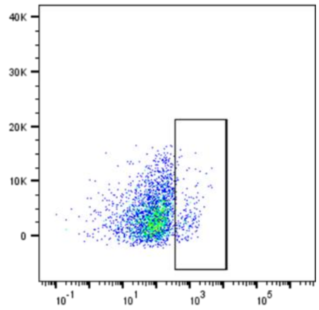

4e-30min-4×MIC
Single Cells
4337

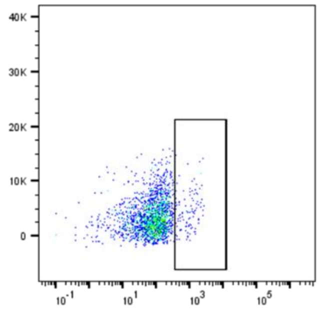

5b-30min-1×MIC
Single Cells
3417

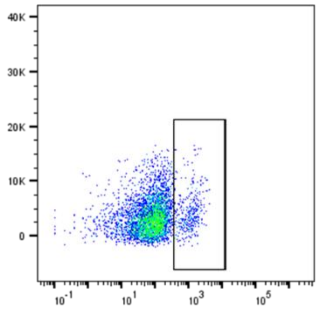

5b-30min-2×MIC
Single Cells
6992

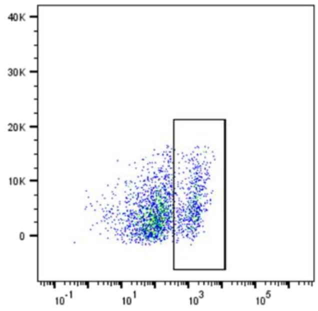

5b-30min-4×MIC
Single Cells
2960

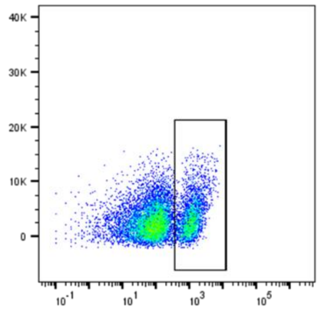

3c-120min-1×MIC
Single Cells
17759

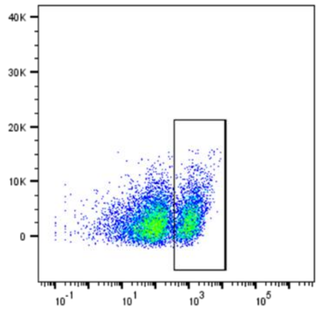

3c-120min-2×MIC
Single Cells
13632

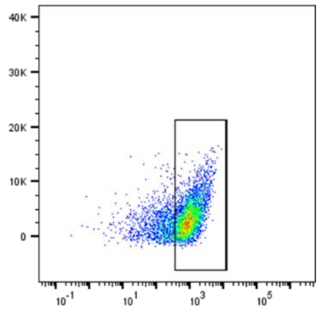

3c-120min-4×MIC
Single Cells
6482

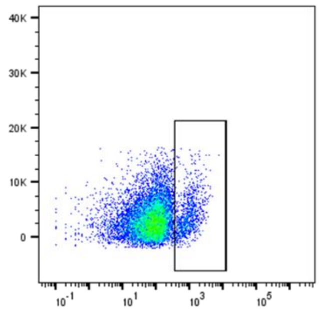

4e-120min-1×MIC
Single Cells
17190

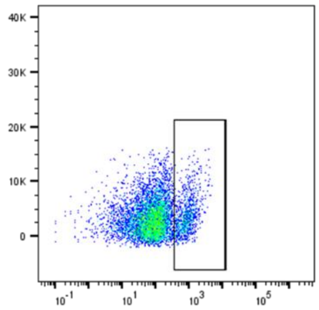

4e-120min-2×MIC
Single Cells
10837

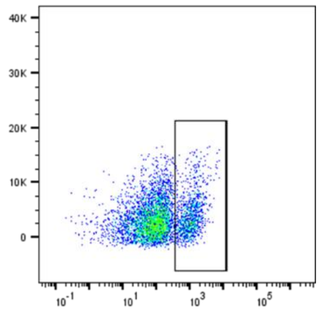

4e-120min-4×MIC
Single Cells
7765

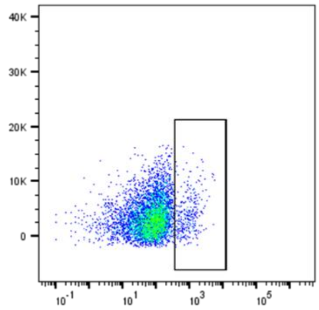

5b-120min-1×MIC
Single Cells
9463

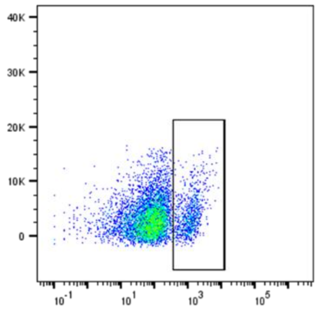

5b-120min-2×MIC
Single Cells
10237

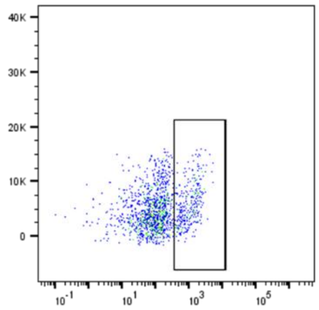

5b-120min-4×MIC
Single Cells
2101

4. ^{1}H NMR, ^{13}C NMR and HRMS Data of Compounds **3a–c**, **4a–e**, **5a–b**, and **6a–f**

Figure S4. ^{1}H NMR, ^{13}C NMR and HRMS data of **3a**

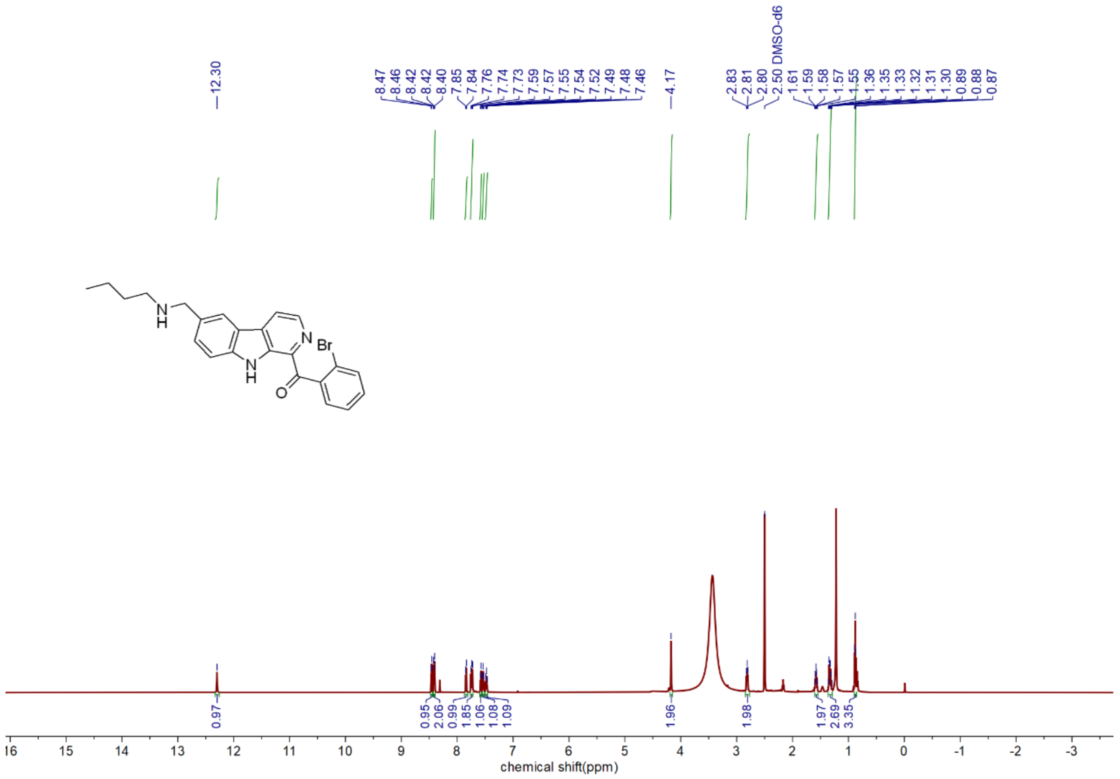
12.30
8.47
8.46
8.42
8.42
8.40
7.85
7.84
7.76
7.74
7.73
7.59
7.57
7.55
7.54
7.52
7.49
7.48
7.46
4.17
2.83
2.81
2.80
2.50 DMSO-d6
1.61
1.59
1.58
1.57
1.55
1.36
1.35
1.33
1.32
1.31
1.30
0.89
0.88
0.87
N
H
N
Br
H
O
0.97
0.95
2.06
0.99
1.85
1.06
1.08
1.09
1.96
1.98
1.97
2.69
3.35
chemical shift(ppm)

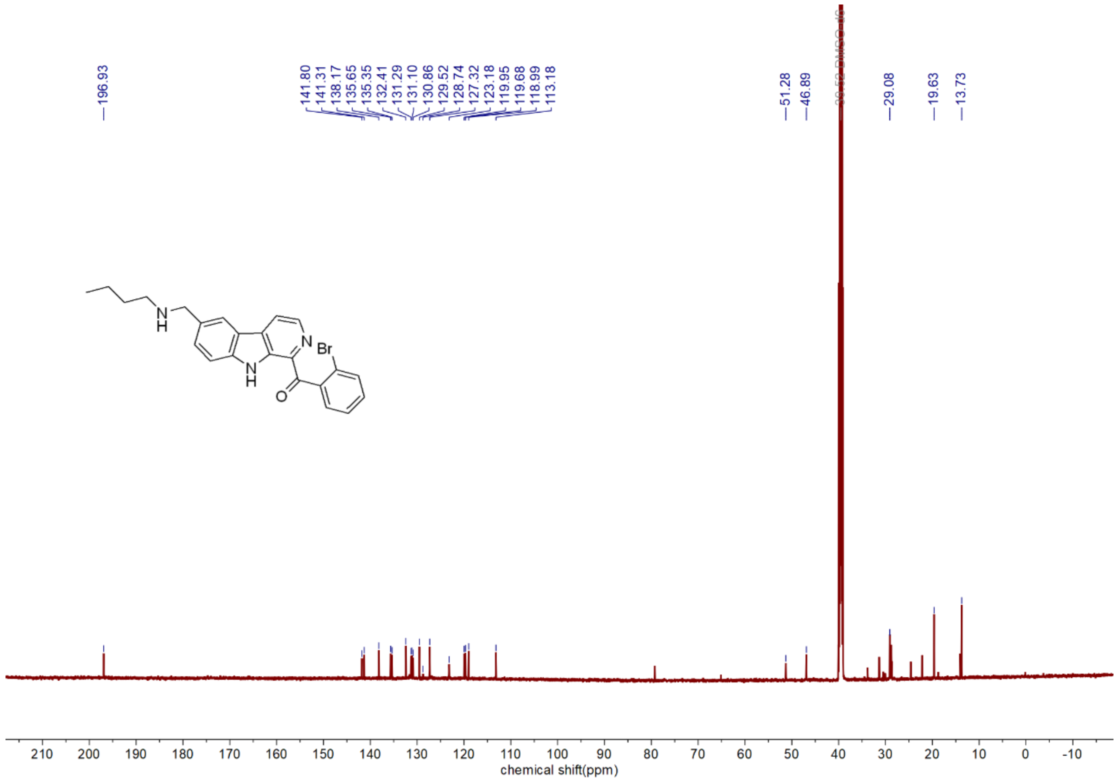
196.93
141.80
141.31
138.17
135.65
135.35
132.41
131.29
131.10
130.86
129.52
128.74
127.32
123.18
119.95
119.68
118.99
113.18
51.28
46.89
29.08
19.63
13.73
N
H
N
Br
H
O
chemical shift(ppm)

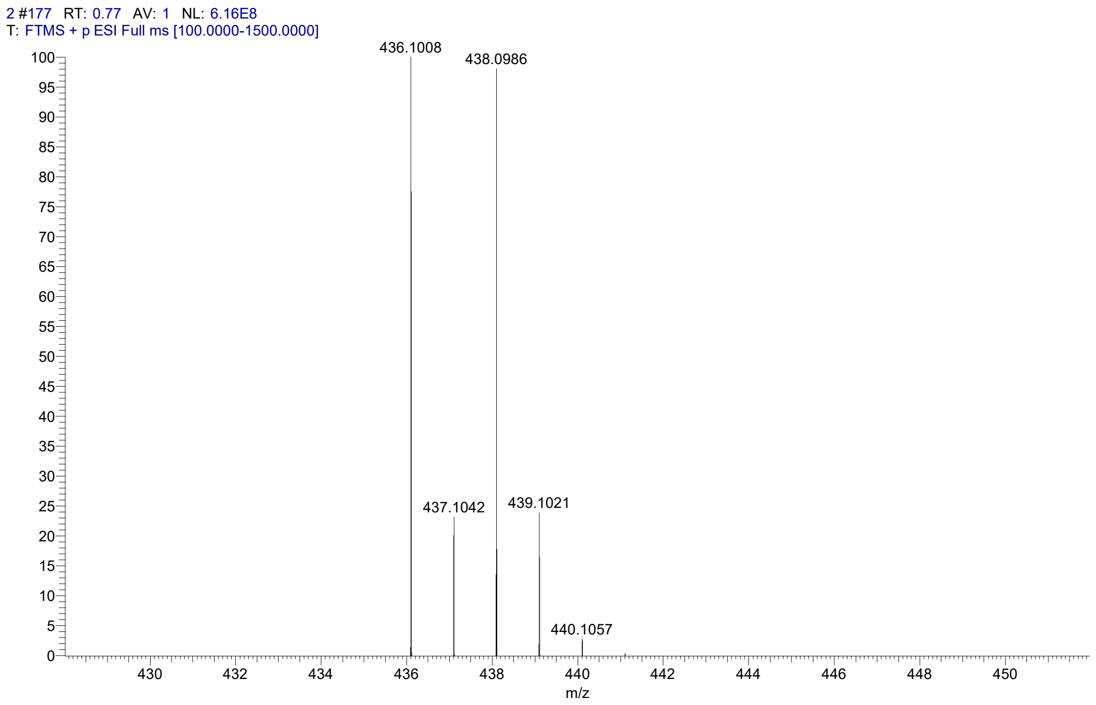

Figure S5. ^{1}H NMR, ^{13}C NMR and HRMS data of **3b**

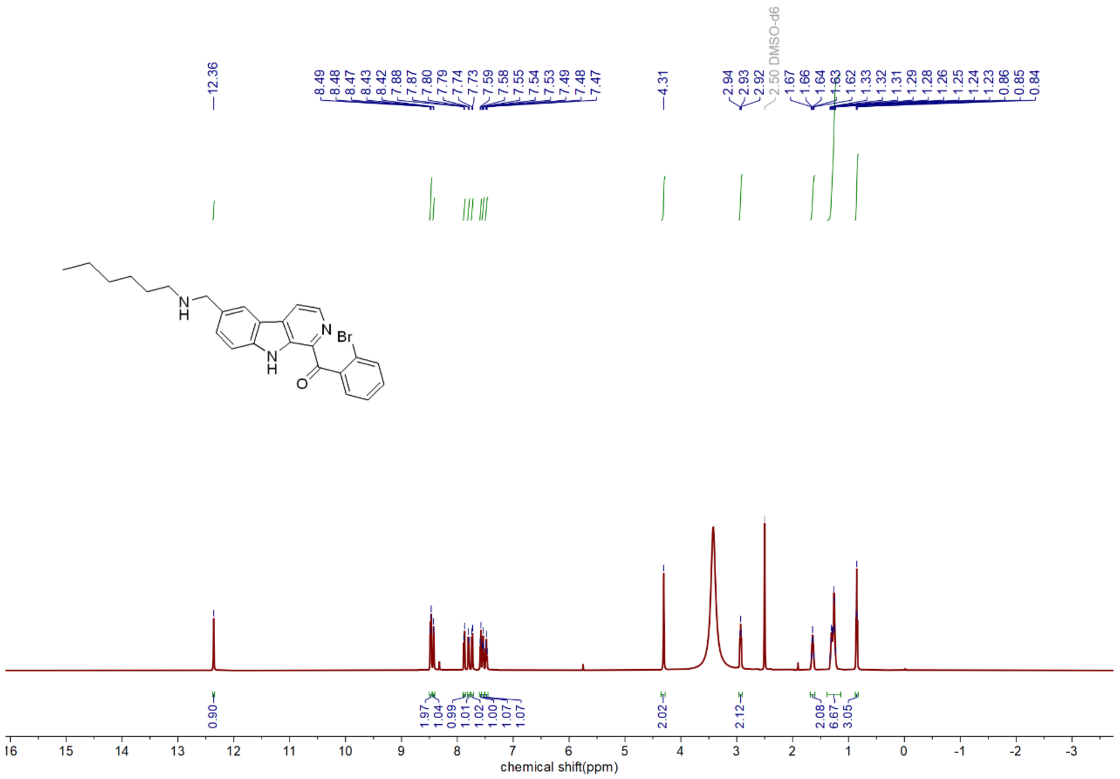

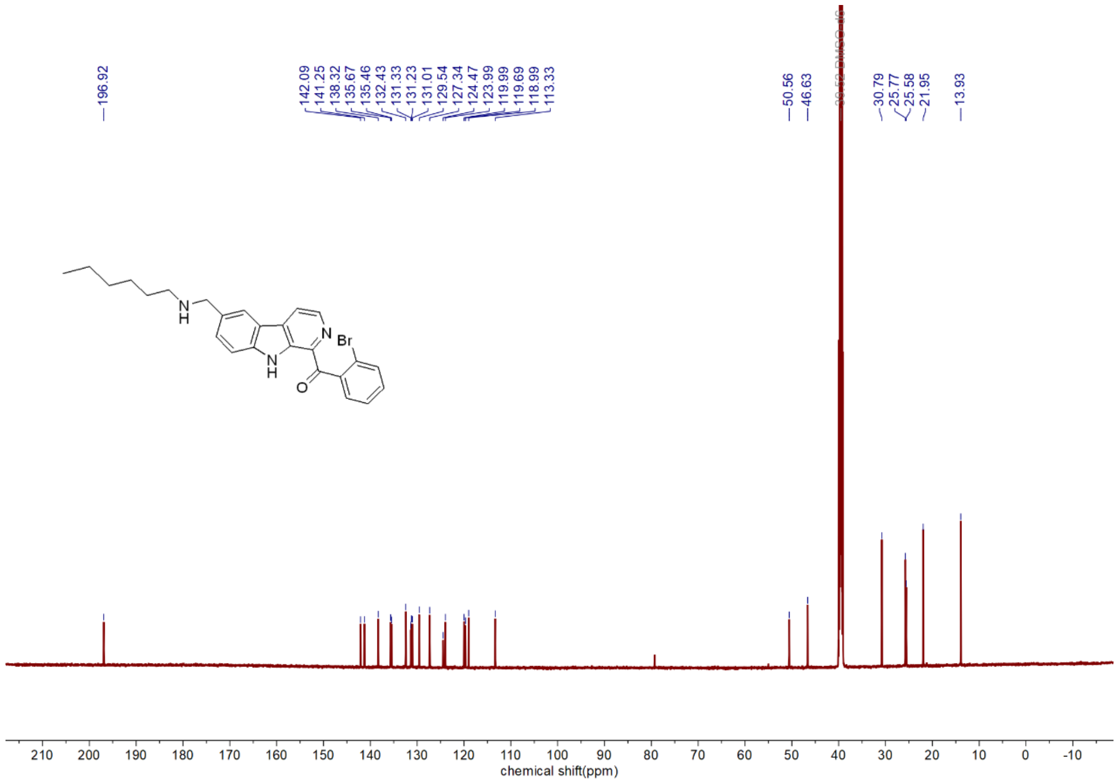

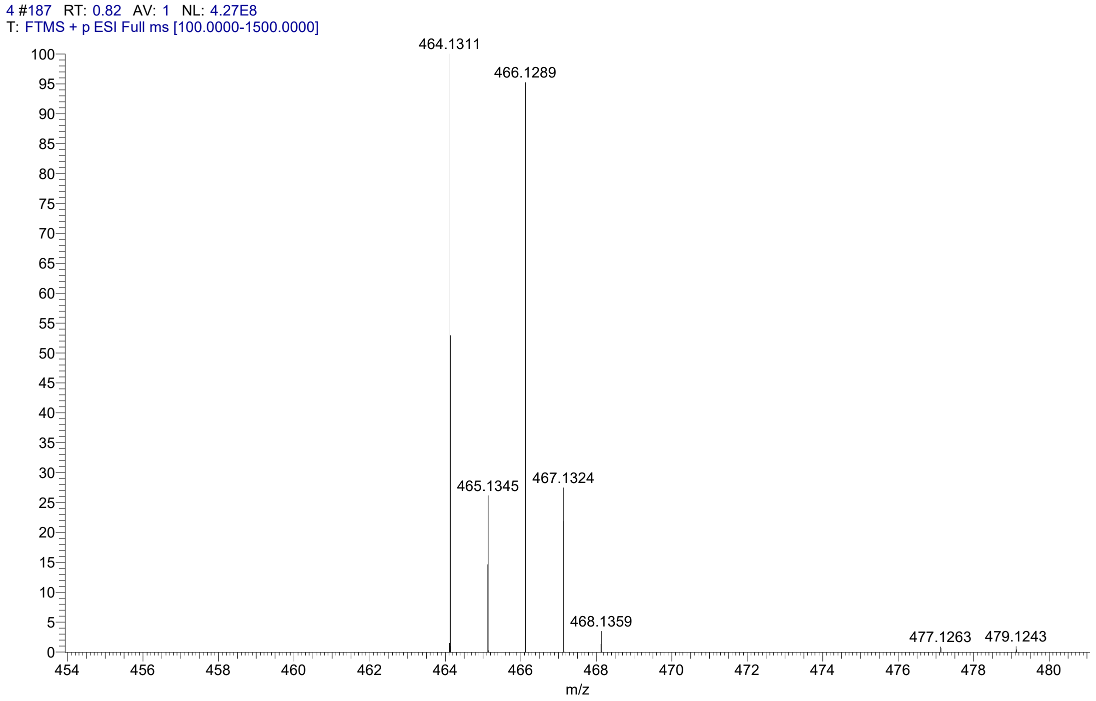

Figure S6. ^{1}H NMR, ^{13}C NMR and HRMS data of **3c**

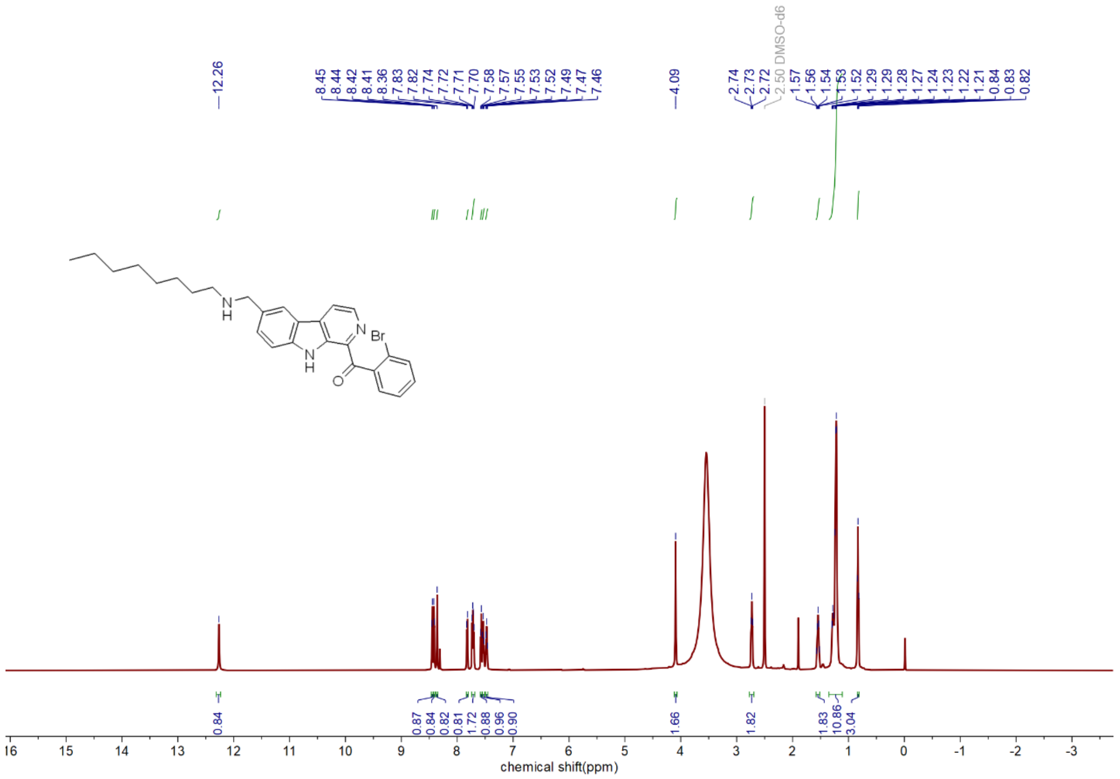
12.26
8.45 8.44 8.42 8.41 8.36 7.83 7.82 7.74 7.72 7.71 7.70 7.58 7.57 7.55 7.53 7.52 7.49 7.47 7.46
4.09
2.74 2.73 2.72 2.50 DMSO-d6 1.57 1.56 1.54 1.53 1.52 1.29 1.29 1.28 1.27 1.24 1.23 1.22 1.21 0.84 0.83 0.82
N
H
N
Br
N
H
O
0.84 0.87 0.84 0.82 0.81 1.72 0.88 0.96 0.90 1.66 1.82 1.83 10.86 3.04
chemical shift(ppm)

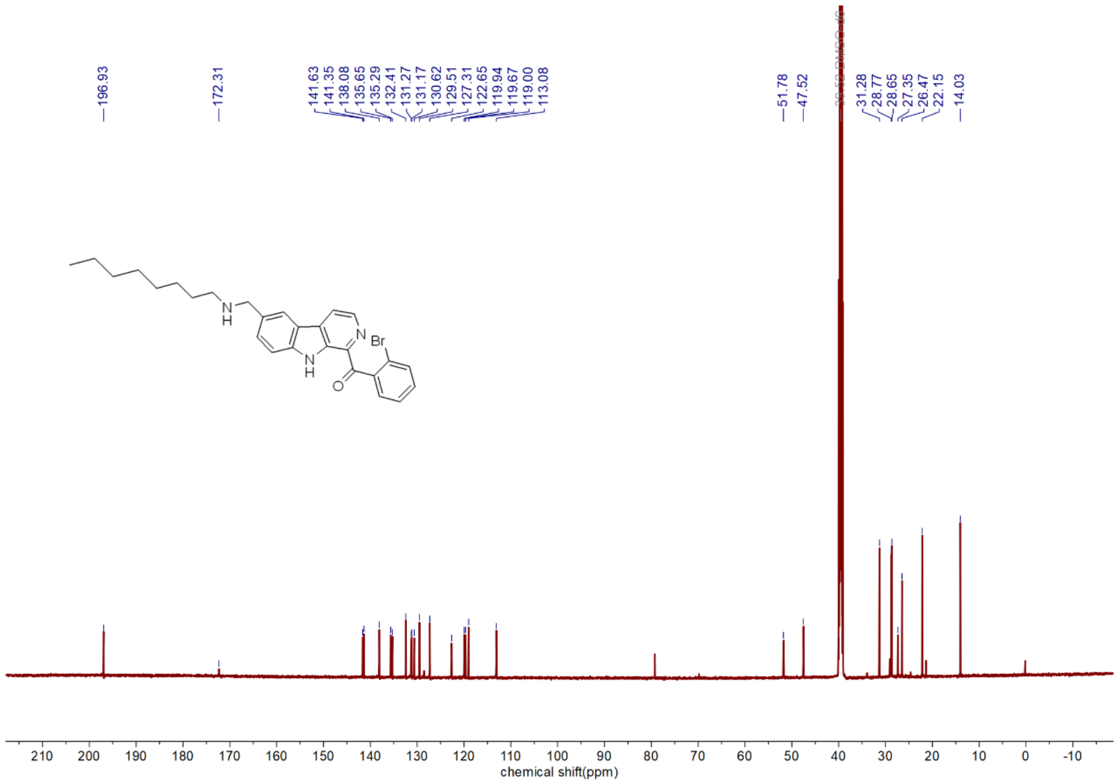
196.93
172.31
141.63 141.35 138.08 135.65 135.29 132.41 131.27 131.17 130.62 129.51 127.31 122.65 119.94 119.67 119.00 113.08
51.78
47.52
31.28 28.77 28.65 27.35 26.47 22.15
14.03
N
H
N
Br
N
H
O
chemical shift(ppm)

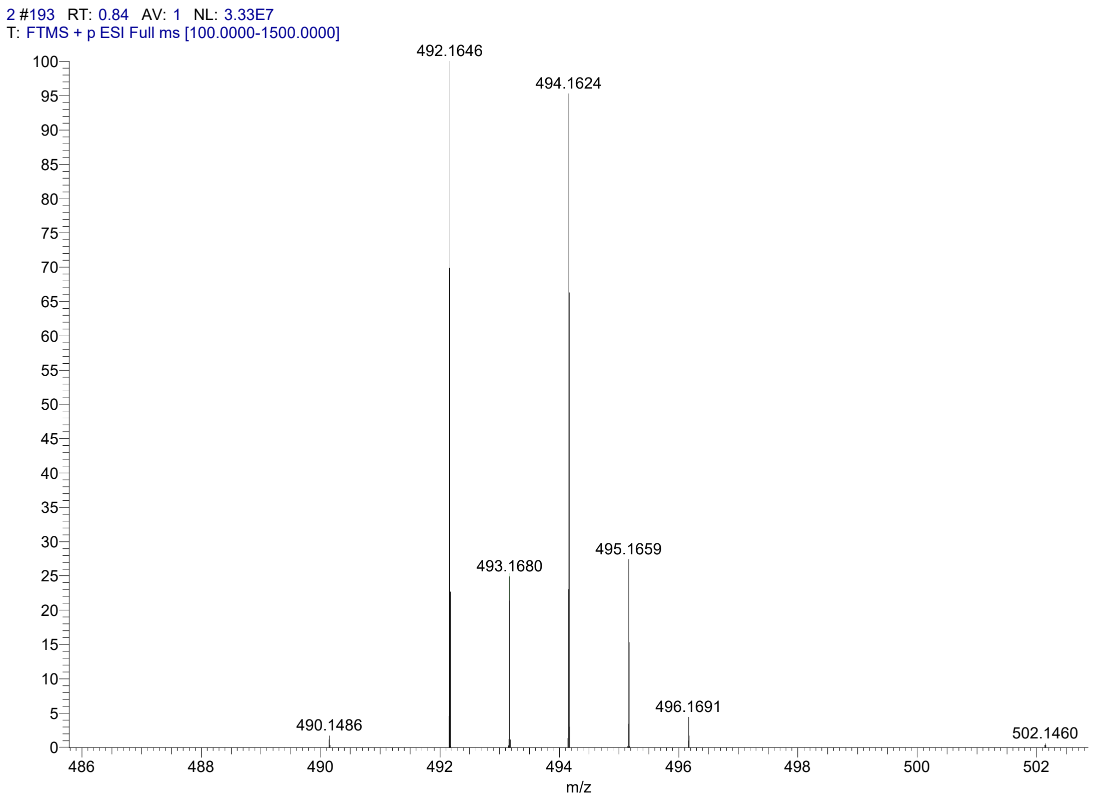

Figure S7. ^{1}H NMR, ^{13}C NMR and HRMS data of **4a**

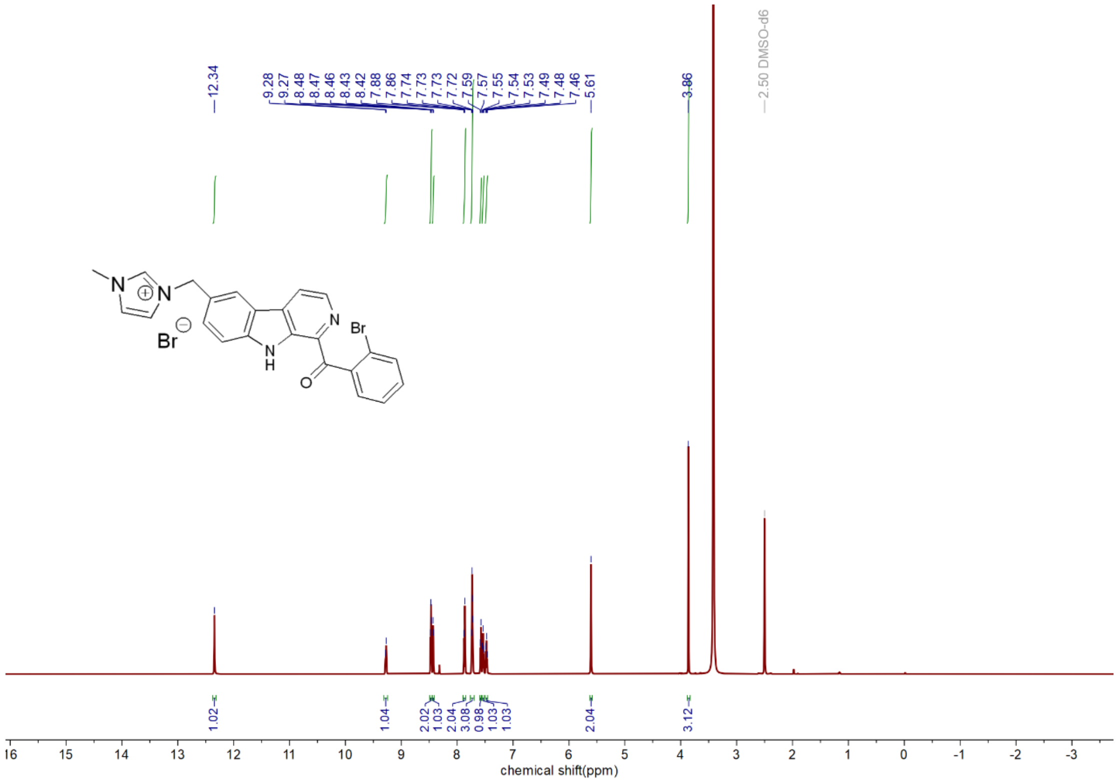

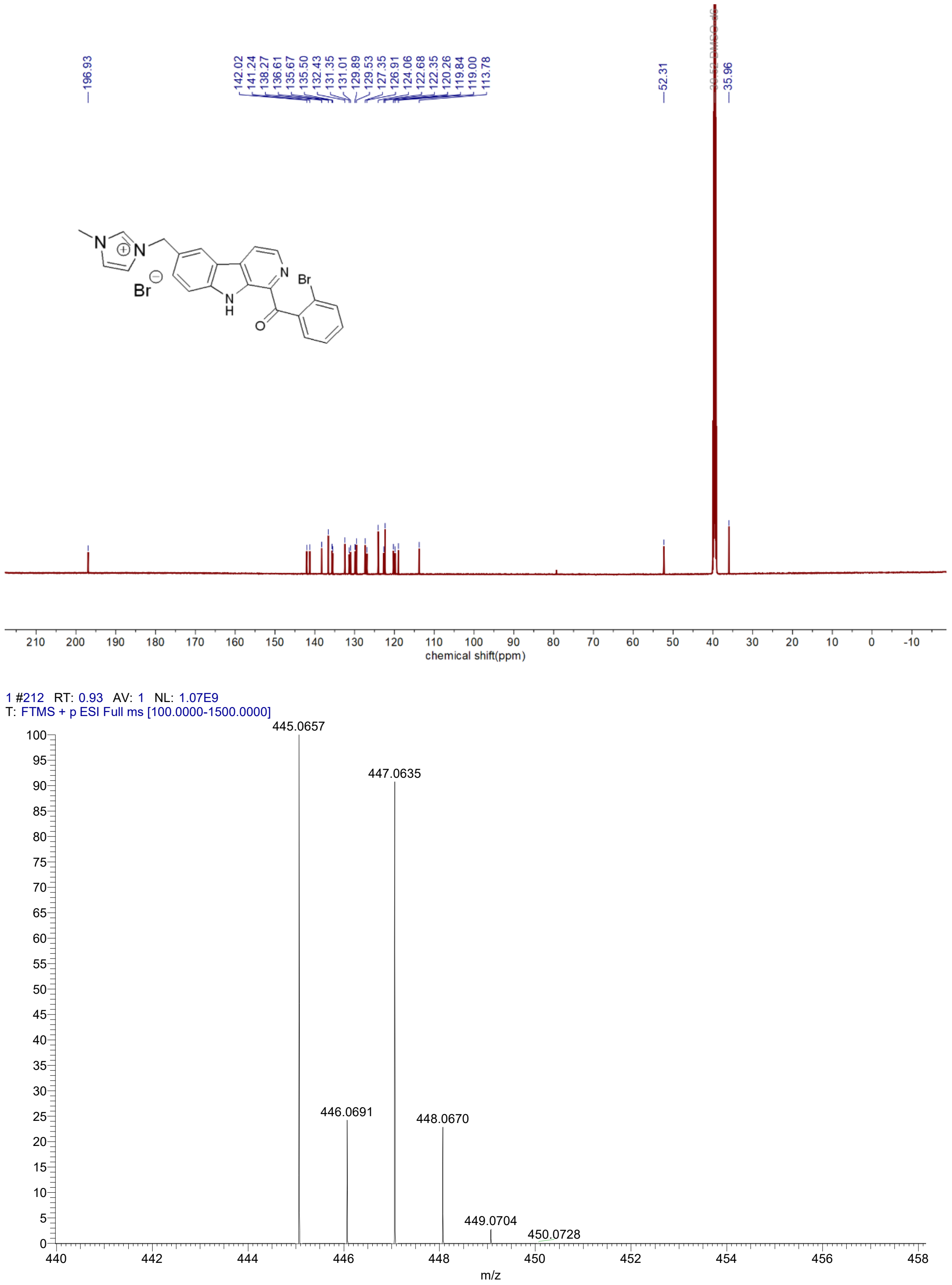

Figure S8. ^{1}H NMR, ^{13}C NMR and HRMS data of **4b**

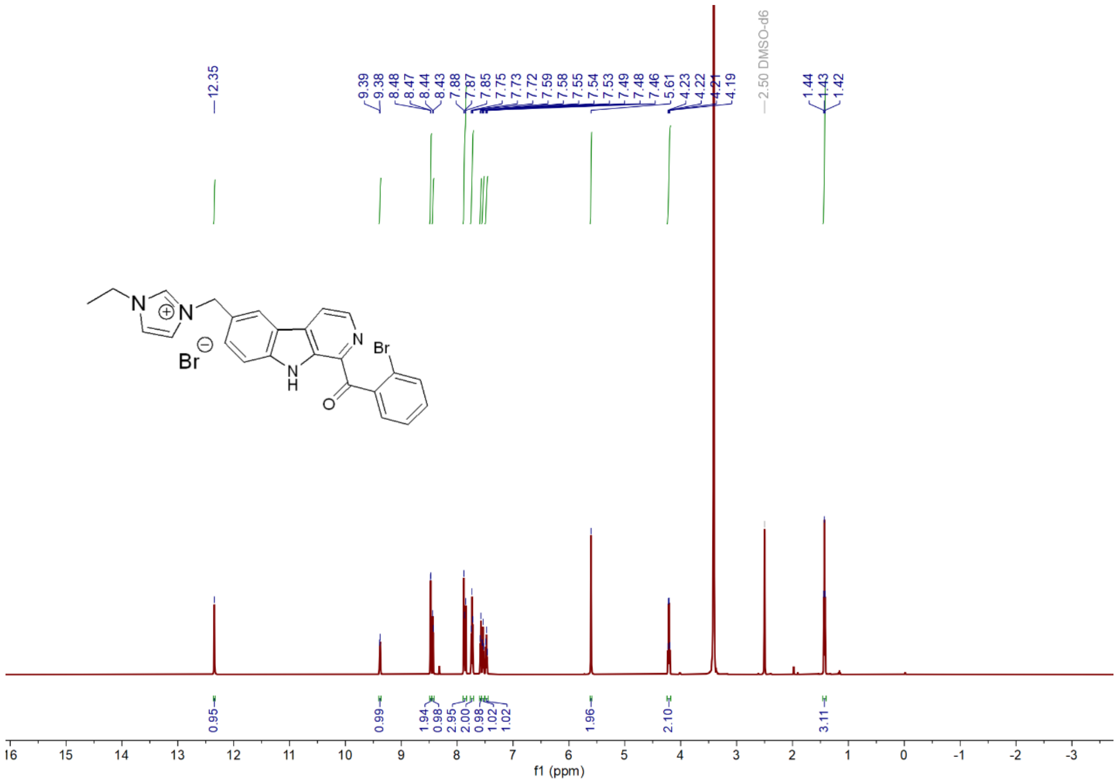

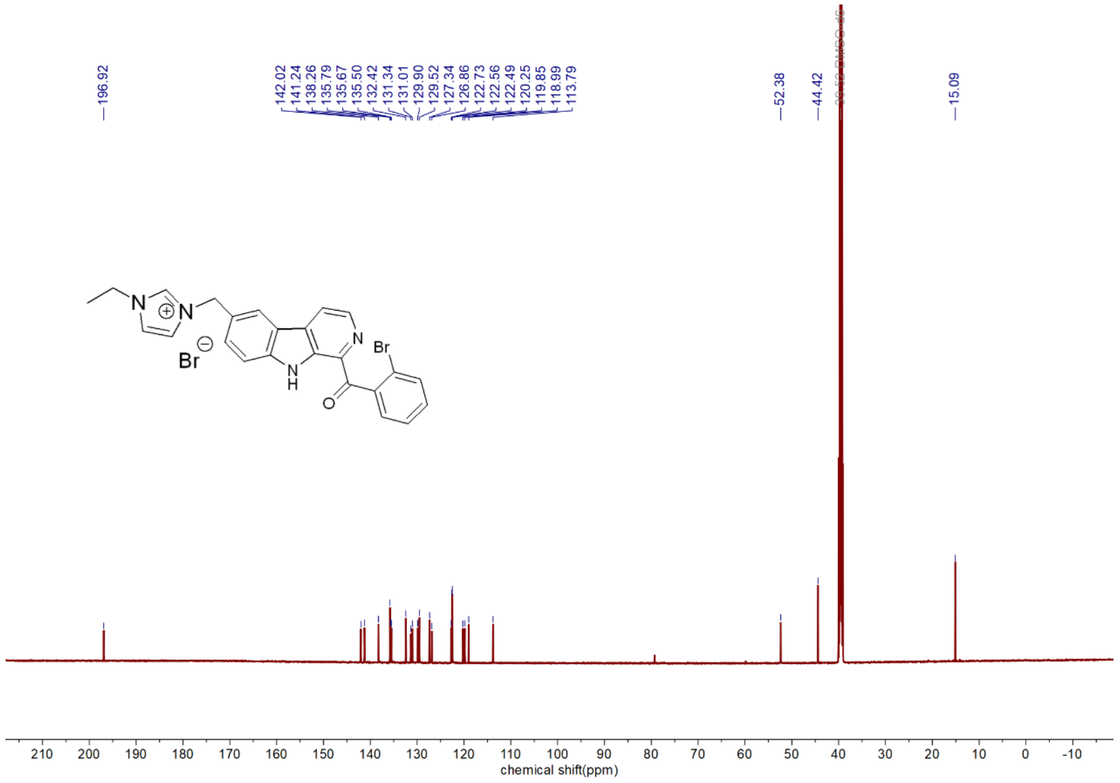

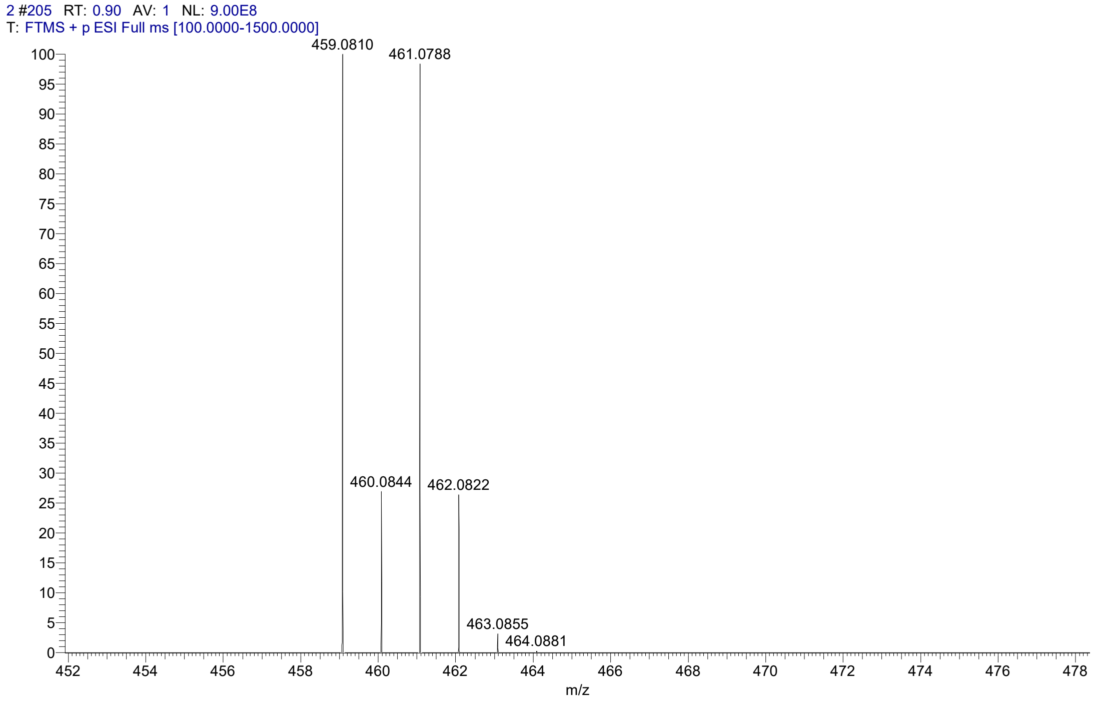

Figure S9. ^{1}H NMR, ^{13}C NMR and HRMS data of **4c**

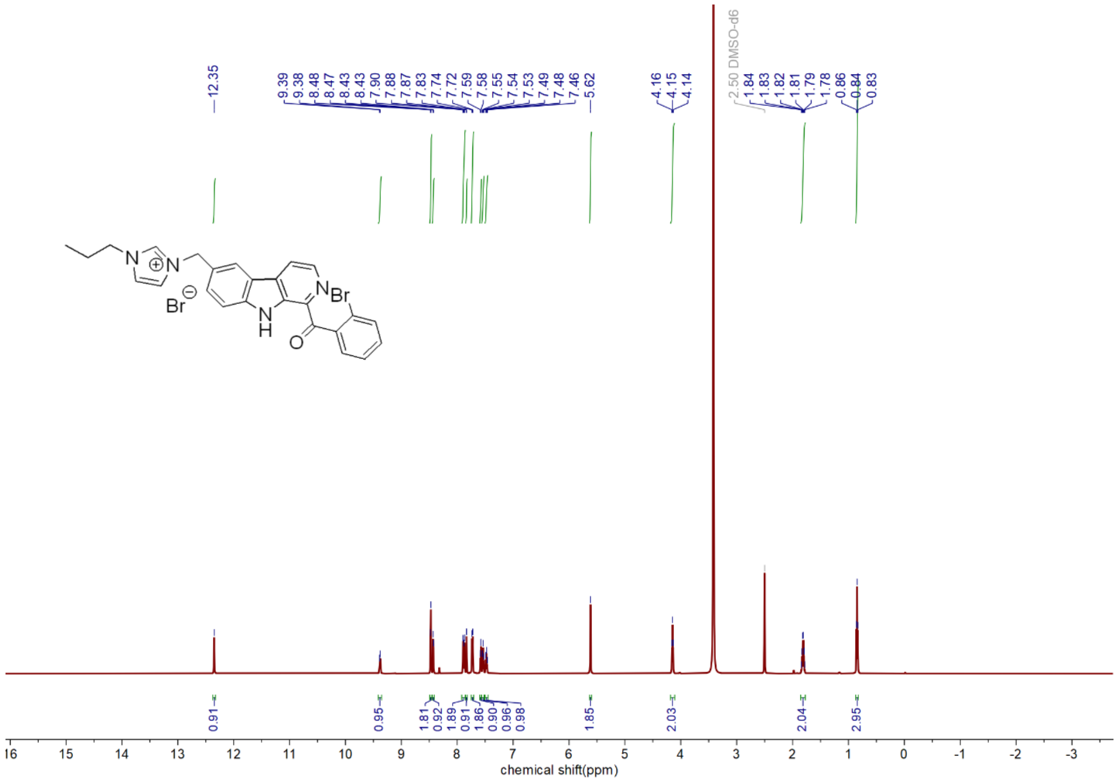

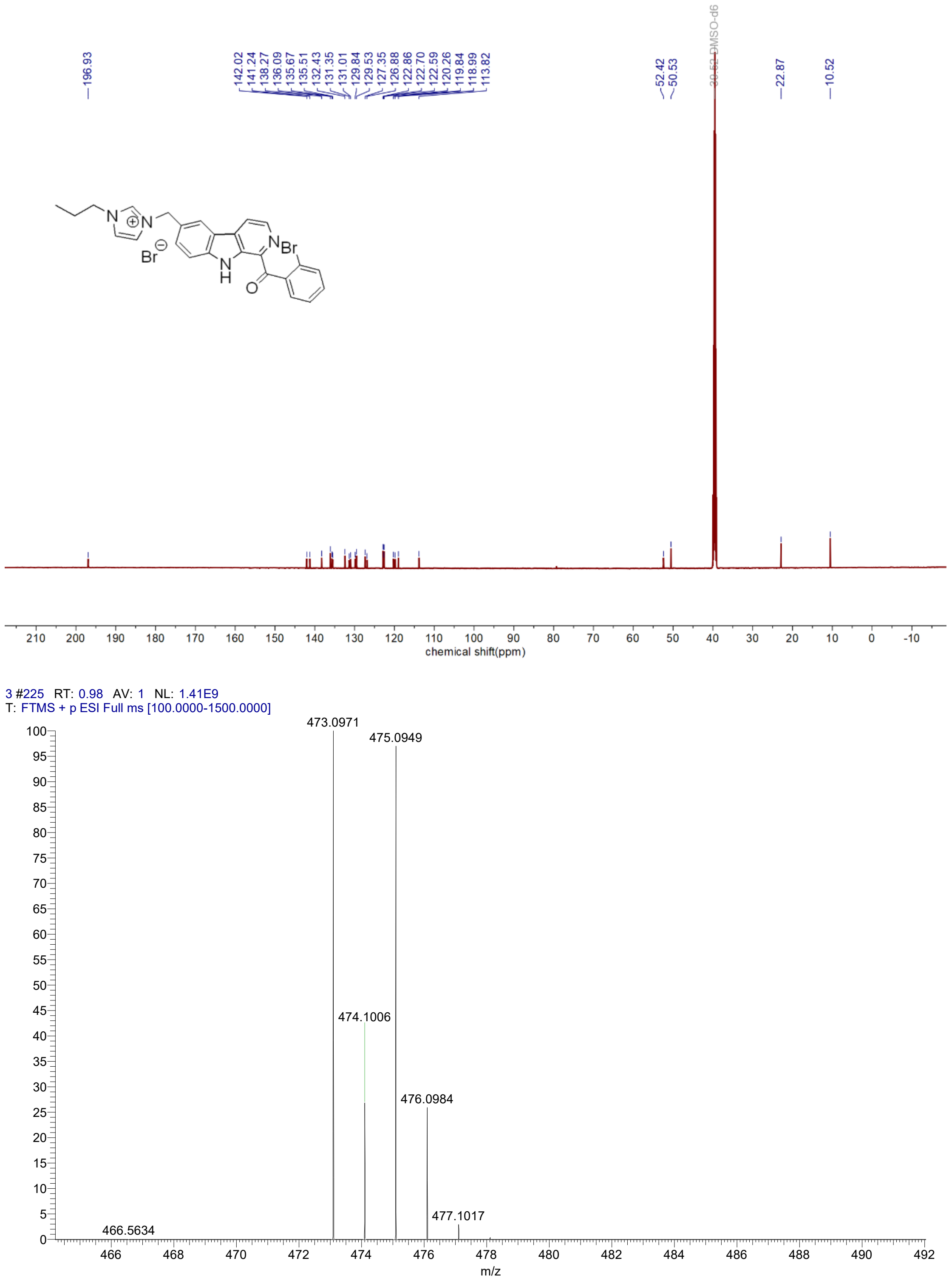

Figure S10. ^{1}H NMR, ^{13}C NMR and HRMS data of **4d**

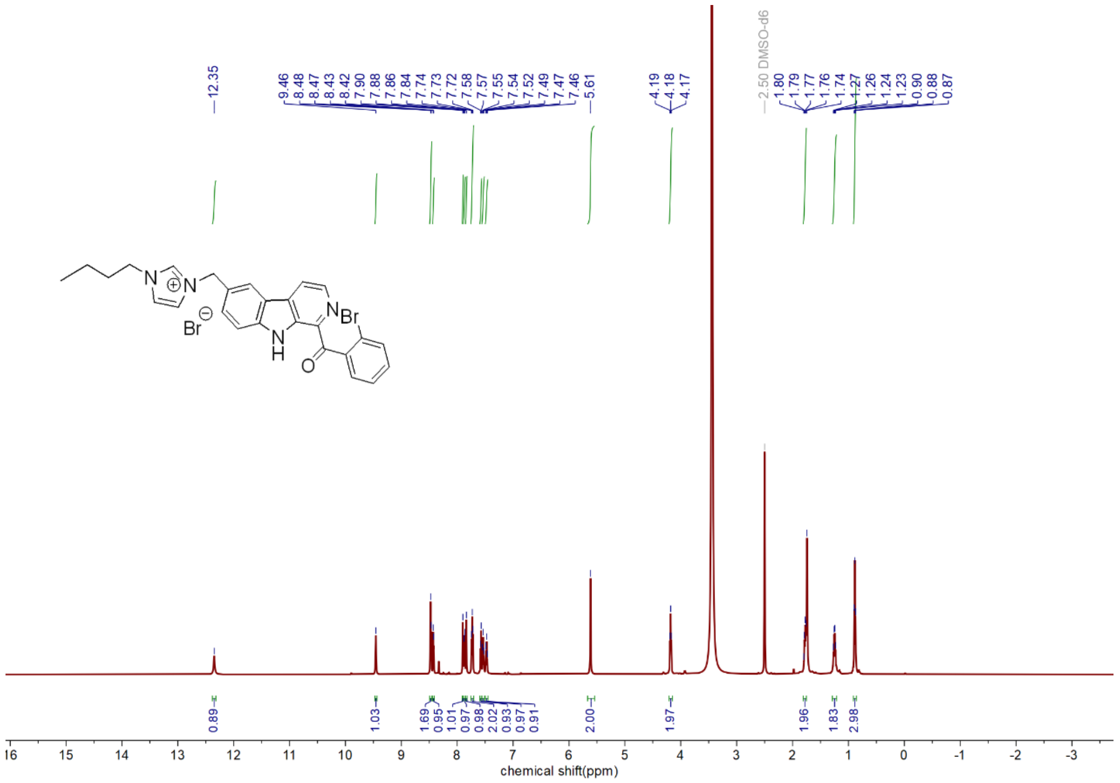
12.35
9.46
8.48
8.47
8.43
8.42
7.90
7.88
7.86
7.84
7.74
7.73
7.72
7.58
7.57
7.55
7.54
7.52
7.49
7.47
7.46
5.61
4.19
4.18
4.17
2.50 DMSO-d6
1.80
1.79
1.77
1.76
1.74
1.27
1.26
1.24
1.23
0.90
0.88
0.87
0.89
1.03
1.69
0.95
1.01
0.97
0.98
2.02
0.93
0.97
0.91
2.00
1.97
1.96
1.83
2.98
Br
N
N
N
Br
N
H
O
chemical shift(ppm)

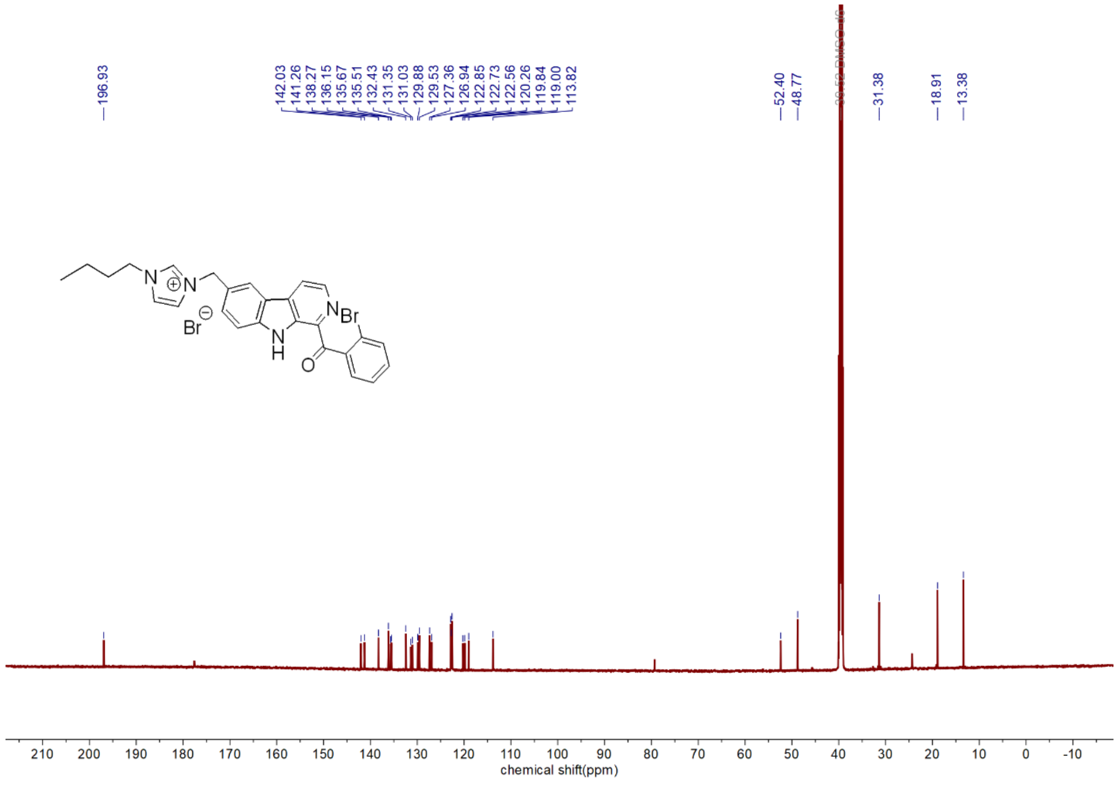
196.93
142.03
141.26
138.27
136.15
135.67
135.51
132.43
131.35
131.03
129.88
129.53
127.36
126.94
122.85
122.73
122.56
120.26
119.84
119.00
113.82
52.40
48.77
31.38
18.91
13.38
Br
N
N
N
Br
N
H
O
chemical shift(ppm)

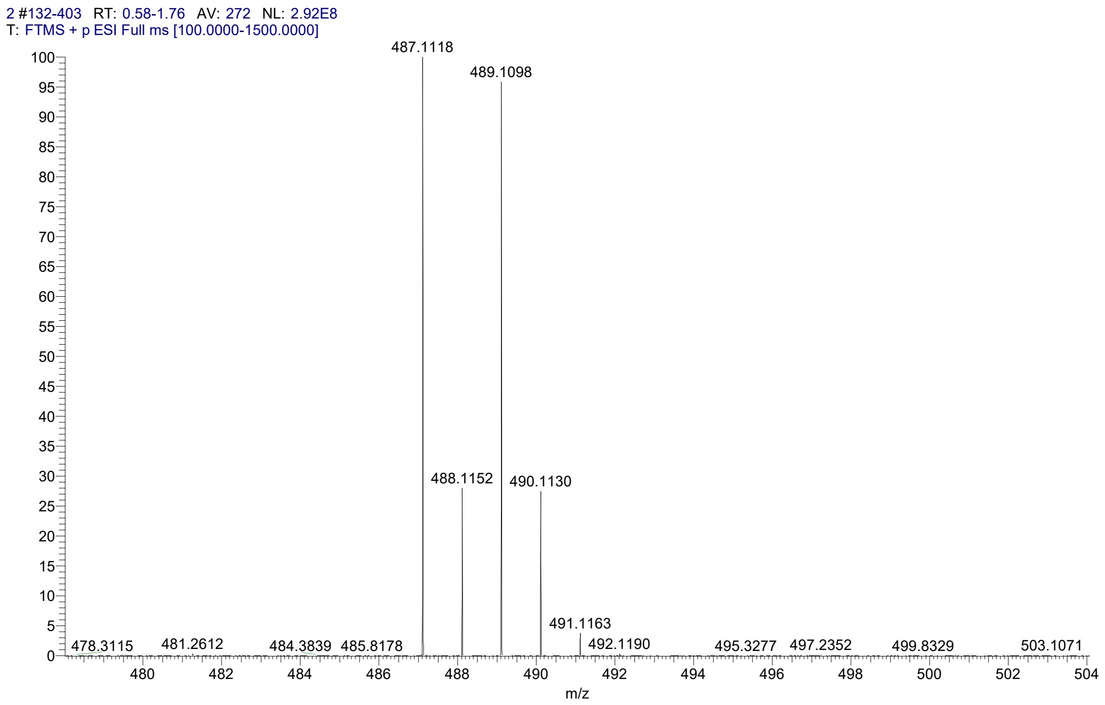

Figure S11. ^{1}H NMR, ^{13}C NMR and HRMS data of **4e**

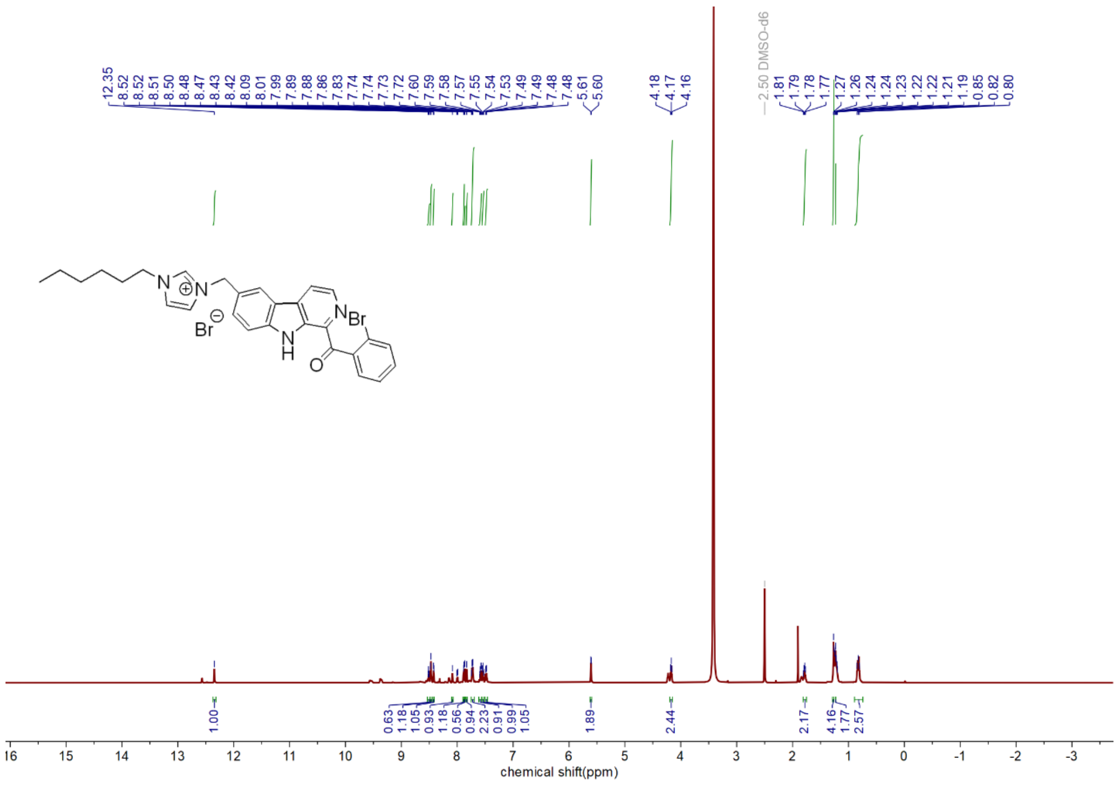

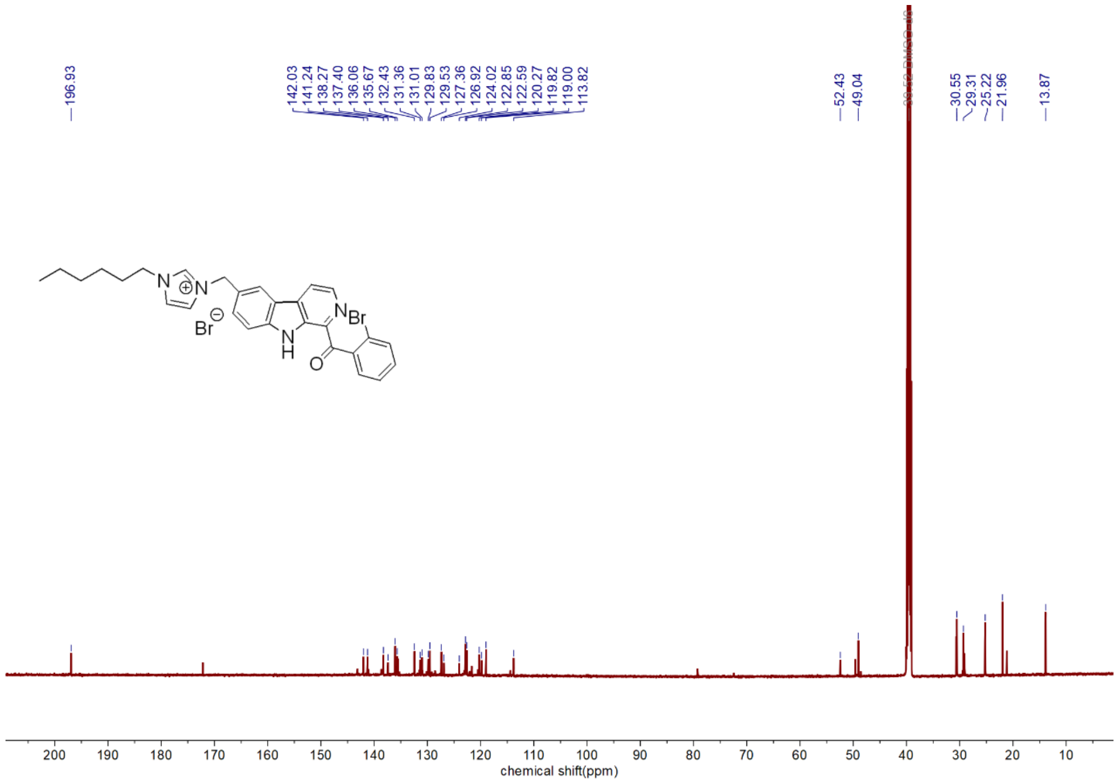

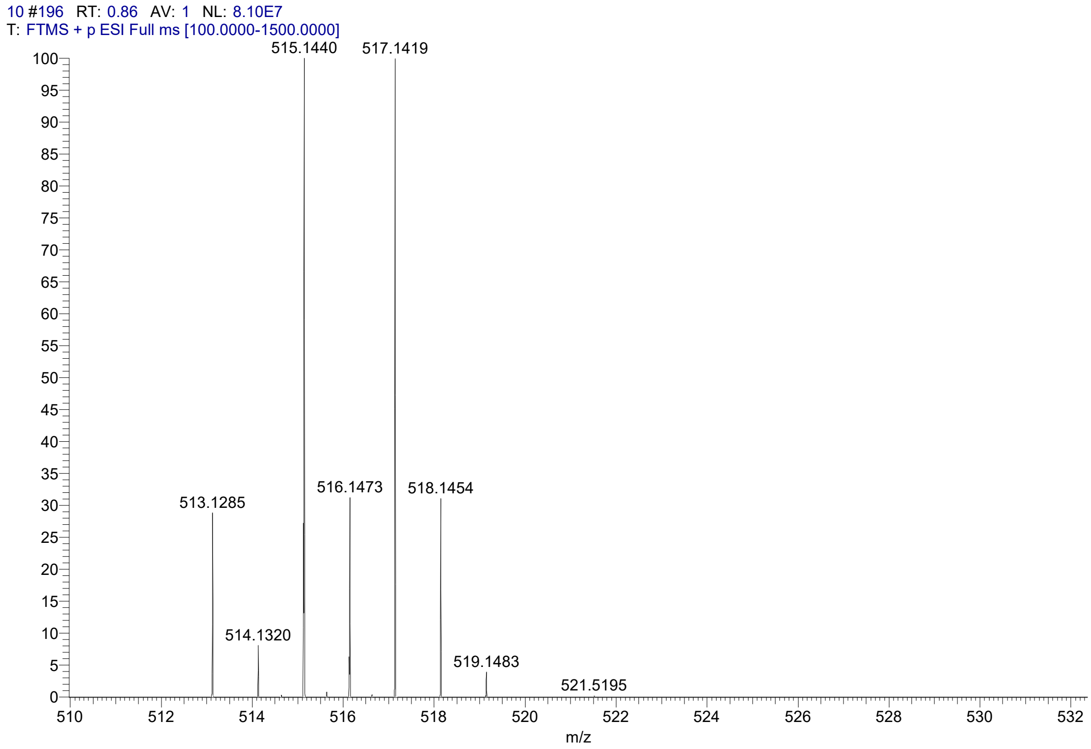

Figure S12. ^{1}H NMR, ^{13}C NMR and HRMS data of **5a**

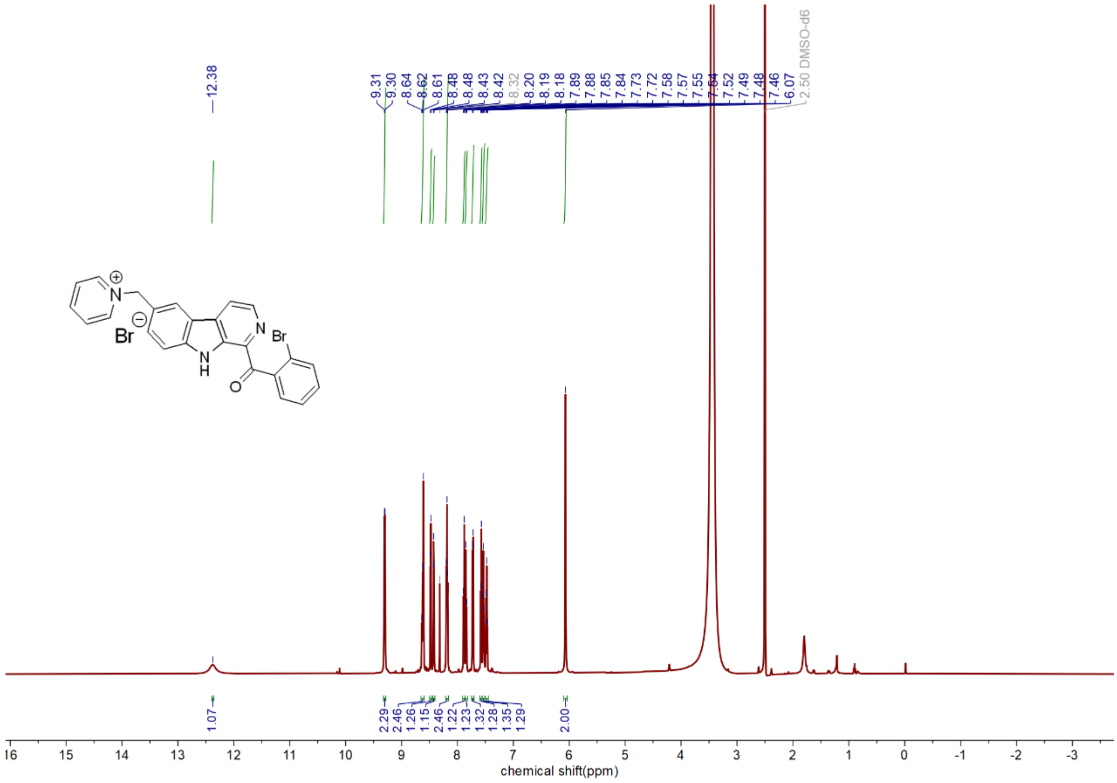
12.38
9.31
9.30
8.64
8.62
8.61
8.48
8.48
8.43
8.42
8.32
8.20
8.19
8.18
7.89
7.88
7.85
7.84
7.73
7.72
7.58
7.57
7.55
7.54
7.52
7.49
7.48
7.46
6.07
2.50 DMSO-d6
Br
N
N
H
O
Br
1.07
2.29
2.46
1.26
1.15
2.46
1.22
1.23
1.32
1.28
1.35
1.29
2.00
chemical shift(ppm)

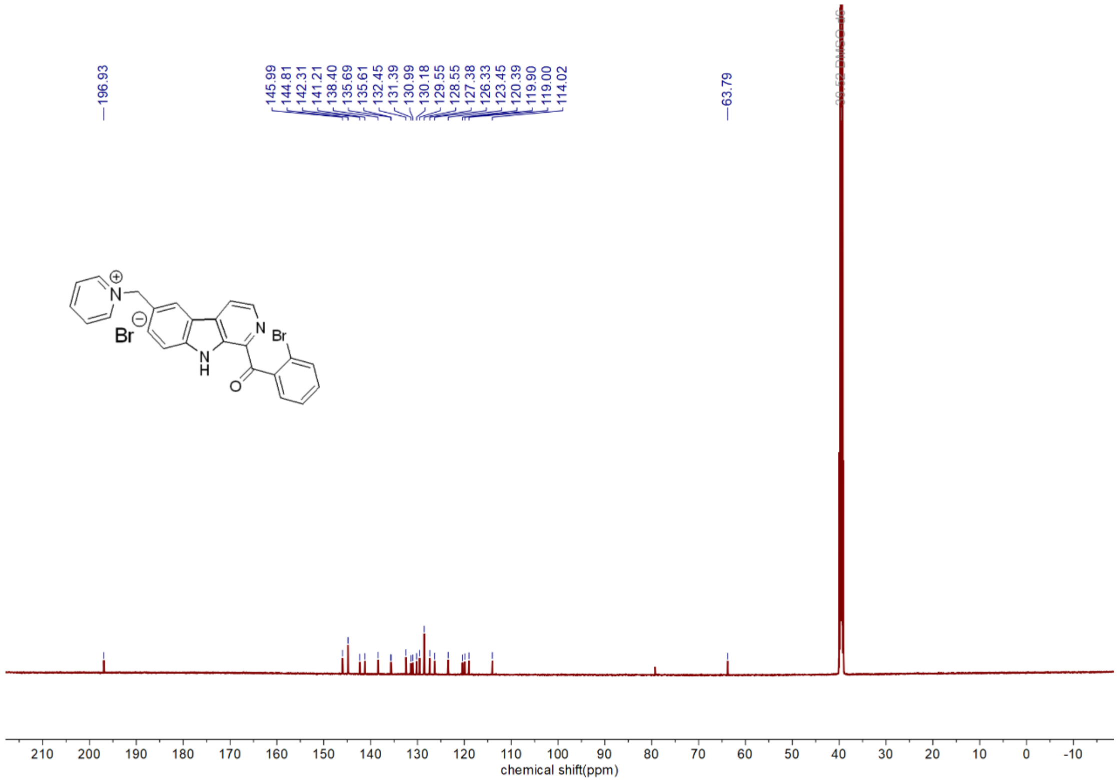
196.93
145.99
144.81
142.31
141.21
138.40
135.69
135.61
132.45
131.39
130.99
130.18
129.55
128.55
127.38
126.33
123.45
120.39
119.90
119.00
114.02
63.79
Br
N
N
H
O
Br
chemical shift(ppm)

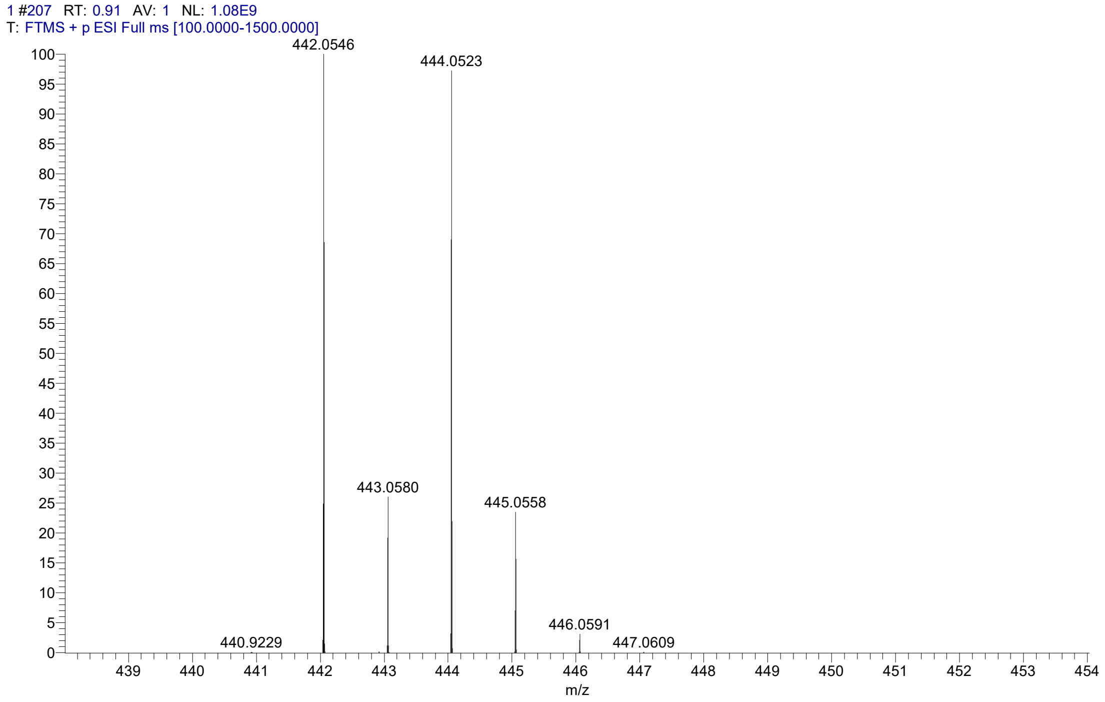

Figure S13. ^{1}H NMR, ^{13}C NMR and HRMS data of **5b**

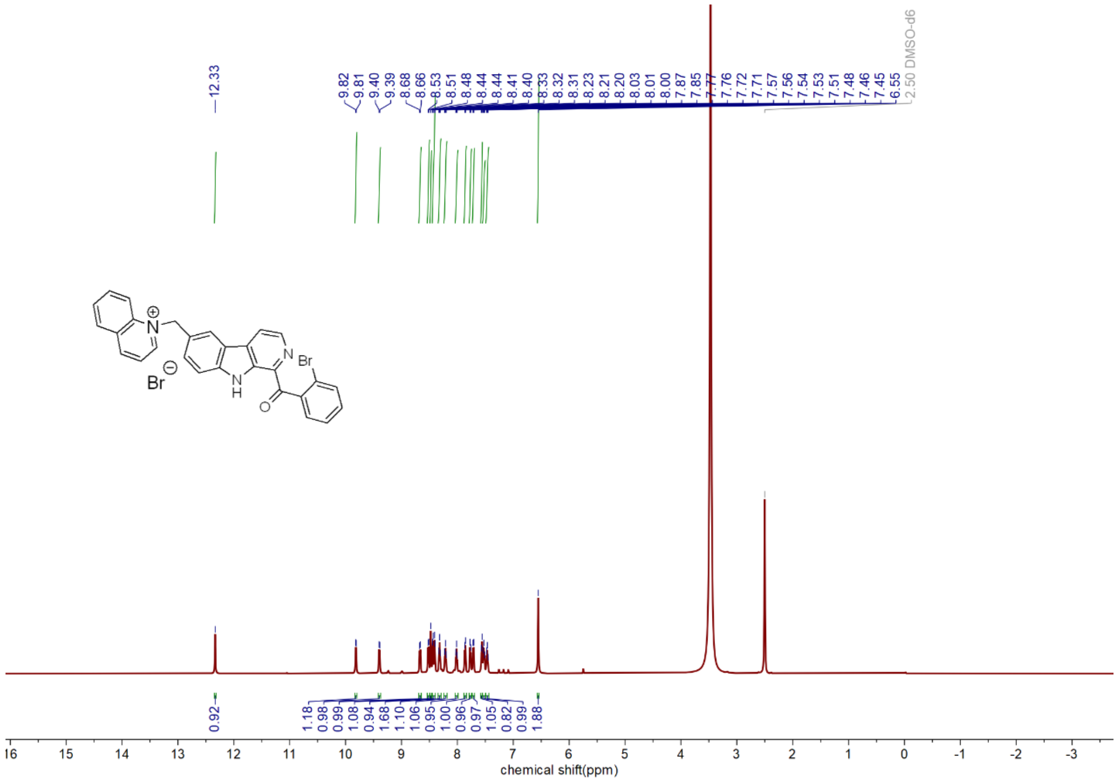

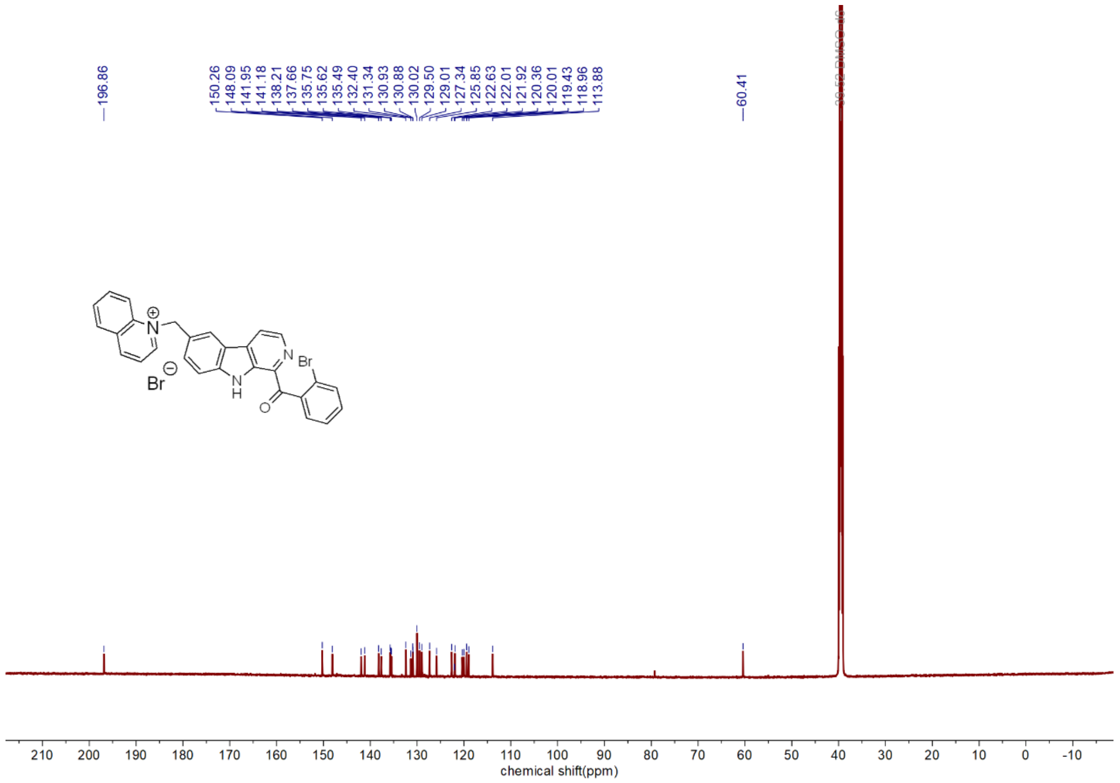

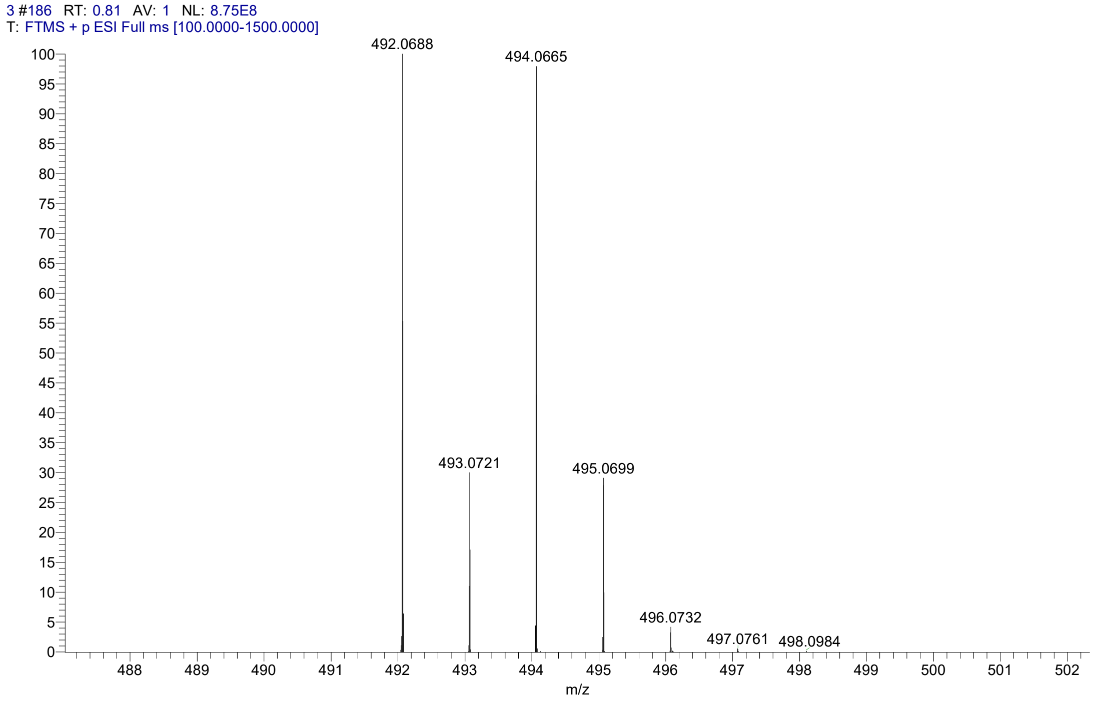

Figure S14. ^{1}H NMR, ^{13}C NMR and HRMS data of **6a**

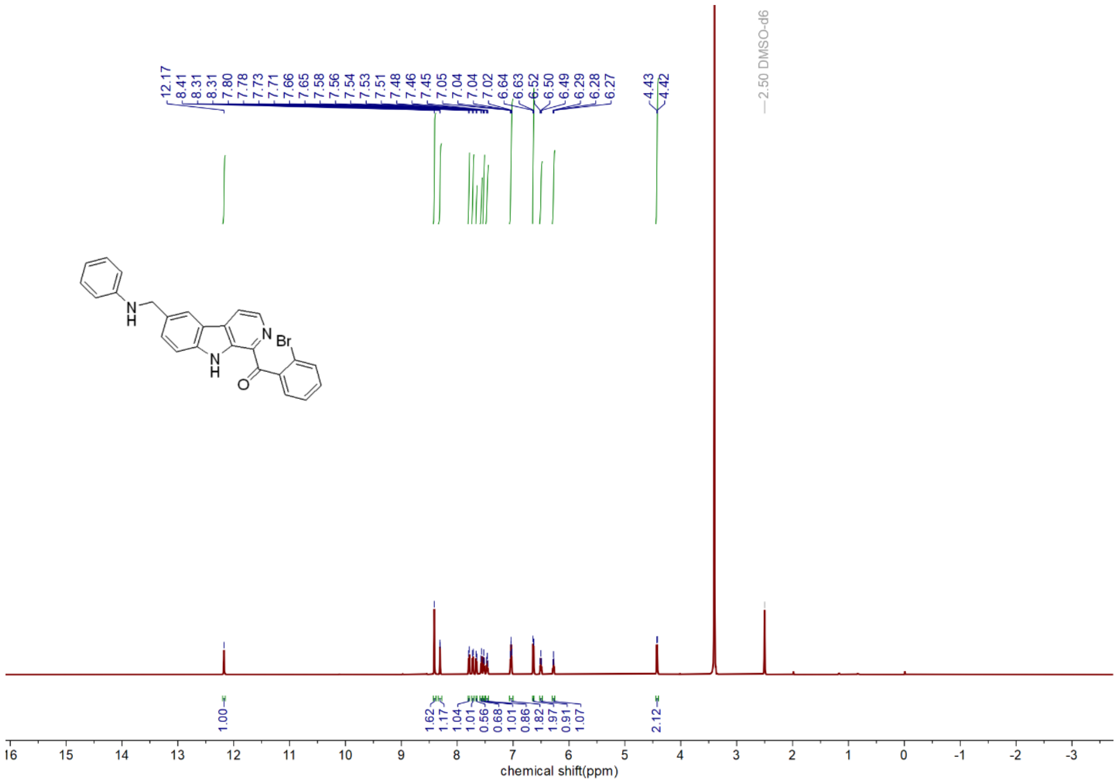
12.17
8.41
8.31
8.31
7.80
7.78
7.73
7.71
7.66
7.65
7.58
7.56
7.54
7.53
7.51
7.48
7.46
7.45
7.05
7.04
7.04
7.02
6.64
6.63
6.52
6.50
6.49
6.29
6.28
6.27
4.43
4.42
2.50 DMSO-d6
N
H
N
Br
N
H
O
1.00
1.62
1.17
1.04
1.01
0.56
0.68
1.01
0.86
1.82
1.97
0.91
1.07
2.12
chemical shift(ppm)

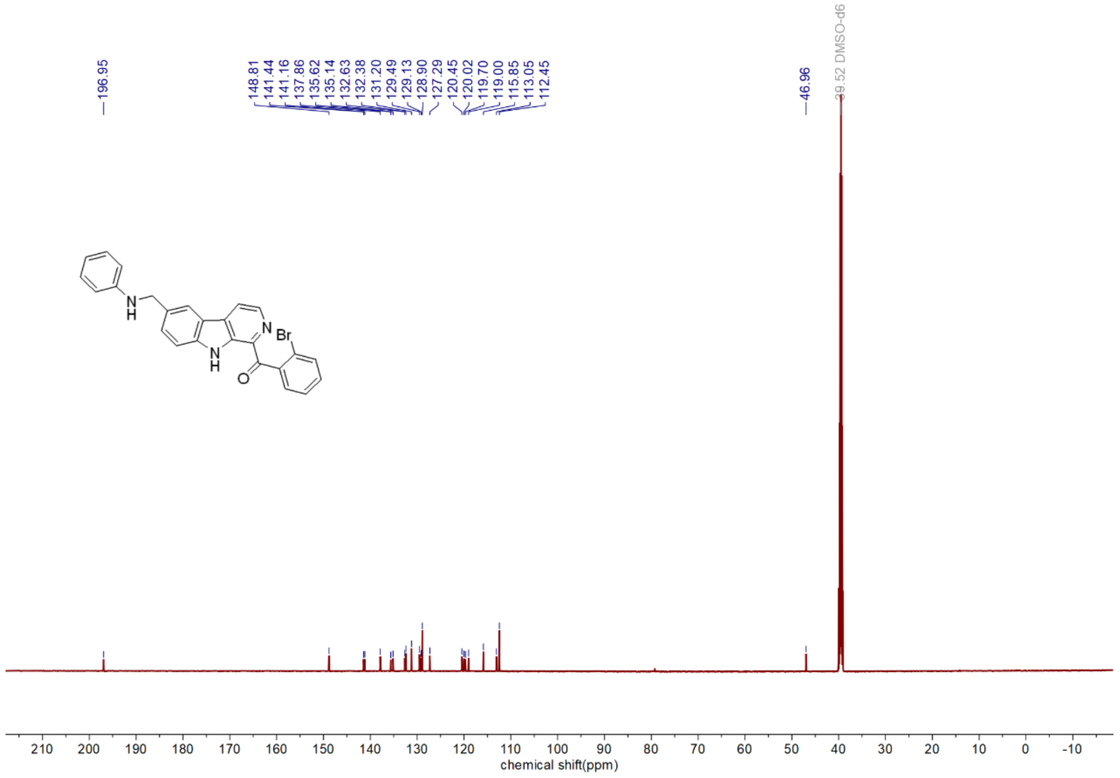
196.95
148.81
141.44
141.16
137.86
135.62
135.14
132.63
132.38
131.20
129.49
129.13
128.90
127.29
120.45
120.02
119.70
119.00
115.85
113.05
112.45
46.96
39.52 DMSO-d6
N
H
N
Br
N
H
O
chemical shift(ppm)

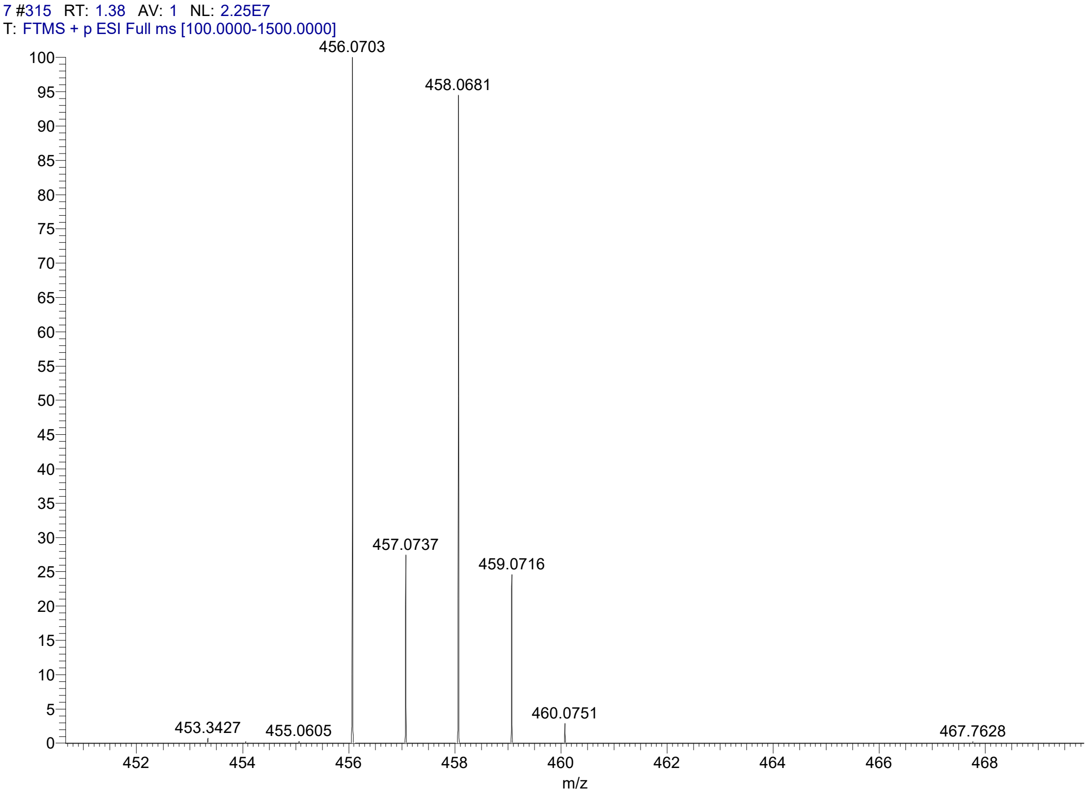

Figure S15. ^{1}H NMR, ^{13}C NMR and HRMS data of **6b**

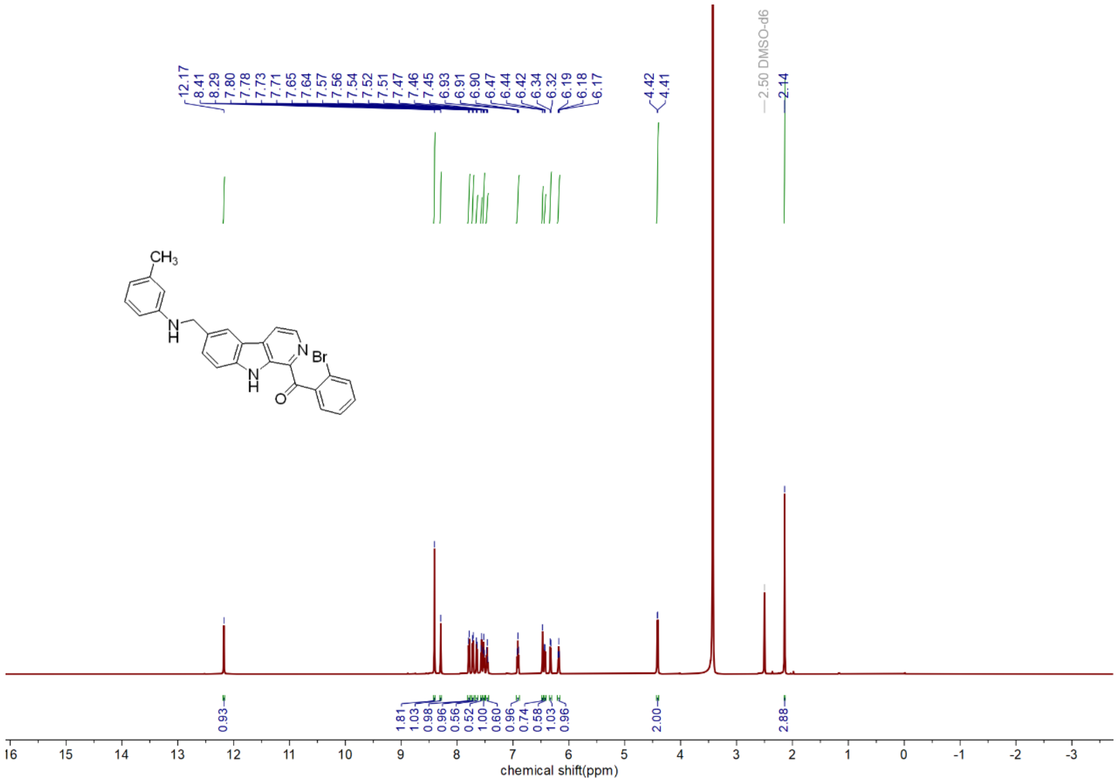

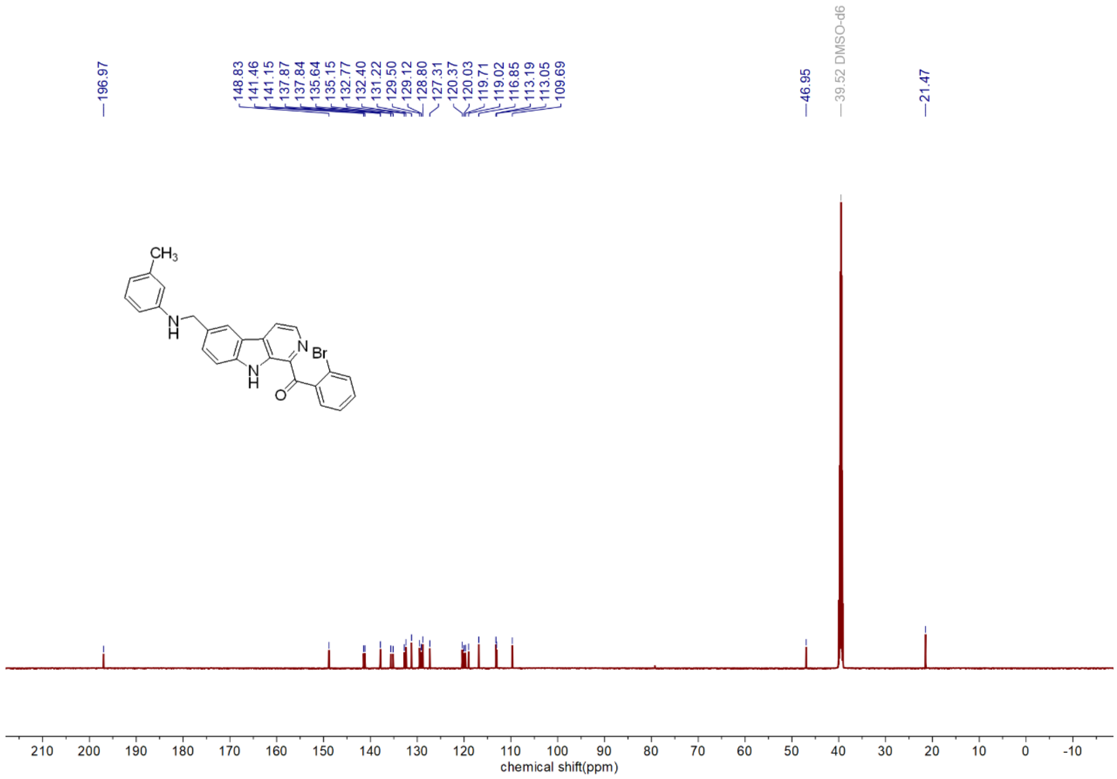

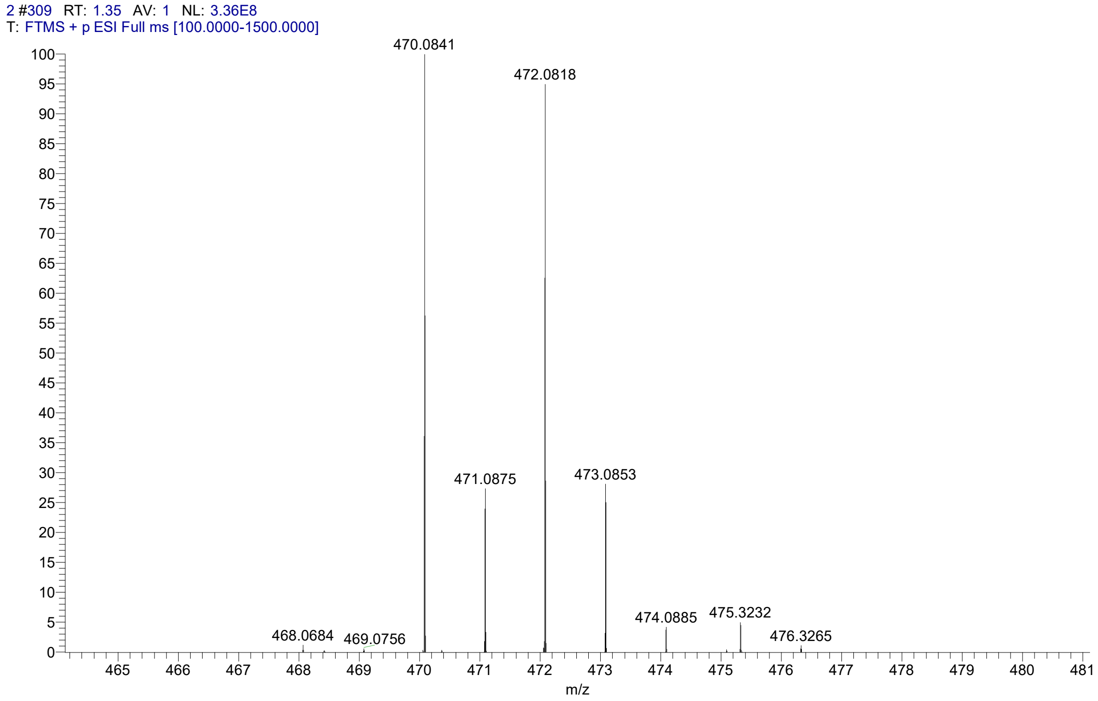

Figure S16. ^{1}H NMR, ^{13}C NMR and HRMS data of **6c**

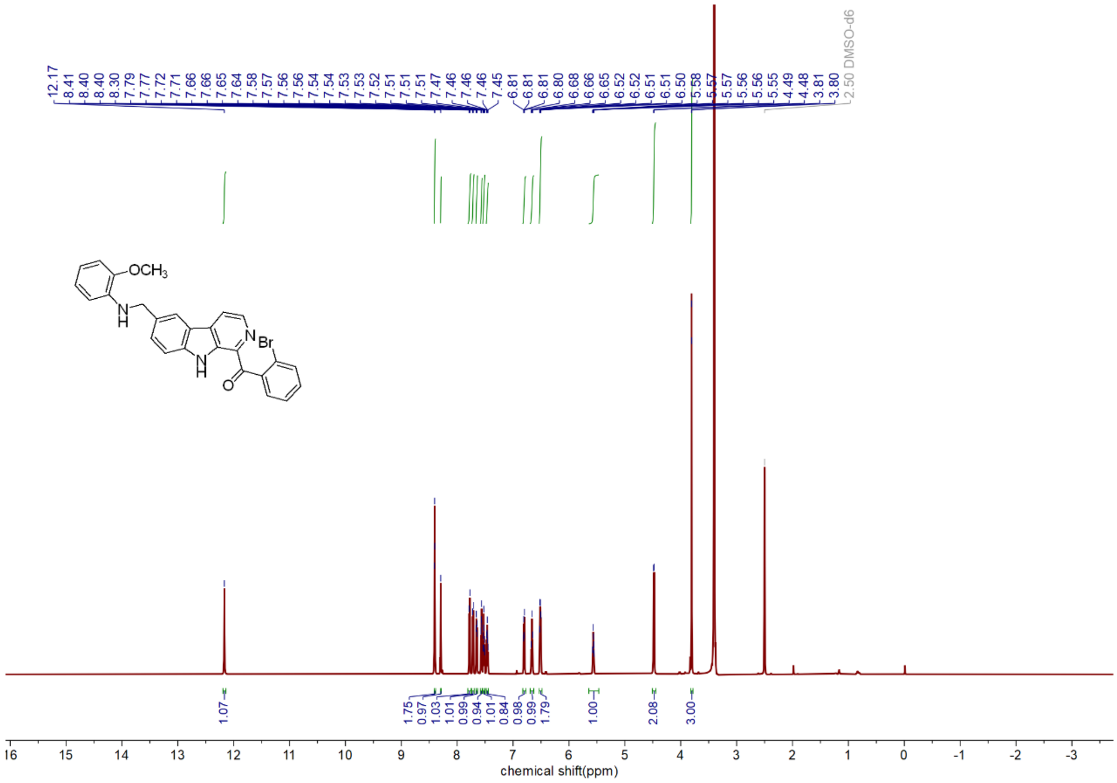
12.17
8.41
8.40
8.40
8.30
7.79
7.77
7.72
7.71
7.66
7.66
7.65
7.64
7.58
7.57
7.56
7.56
7.54
7.54
7.53
7.53
7.52
7.51
7.51
7.51
7.47
7.46
7.46
7.46
7.45
6.81
6.81
6.81
6.80
6.68
6.66
6.65
6.52
6.52
6.51
6.51
6.50
5.58
5.57
5.57
5.56
5.56
5.55
4.49
4.48
3.81
3.80
2.50 DMSO-d6
OCH3
N
H
N
Br
N
H
O
1.07
1.75
0.97
1.03
1.01
0.99
0.94
1.01
0.84
0.98
0.99
1.79
1.00
2.08
3.00
16
15
14
13
12
11
10
9
8
7
6
5
4
3
2
1
0
-1
-2
-3
chemical shift(ppm)

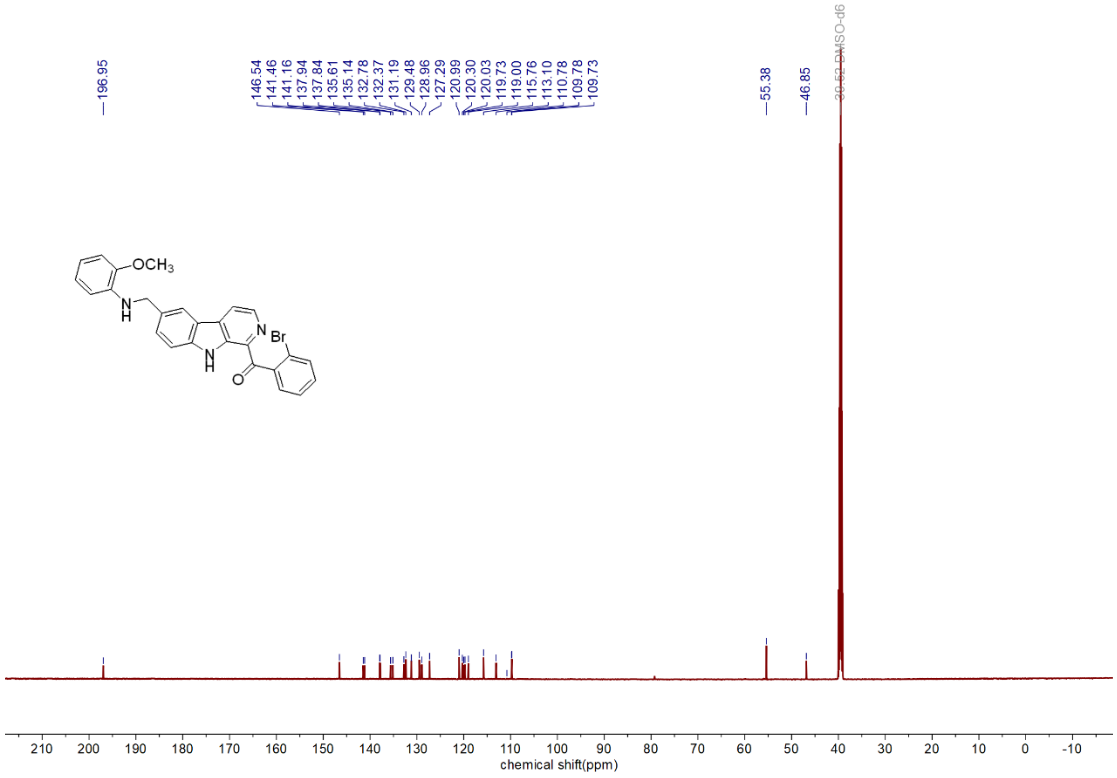
196.95
146.54
141.46
141.16
137.94
137.84
135.61
135.14
132.78
132.37
131.19
129.48
128.96
127.29
120.99
120.30
120.03
119.73
119.00
115.76
113.10
110.78
109.78
109.73
55.38
46.85
39.52 DMSO-d6
OCH3
N
H
N
Br
N
H
O
210
200
190
180
170
160
150
140
130
120
110
100
90
80
70
60
50
40
30
20
10
0
-10
chemical shift(ppm)

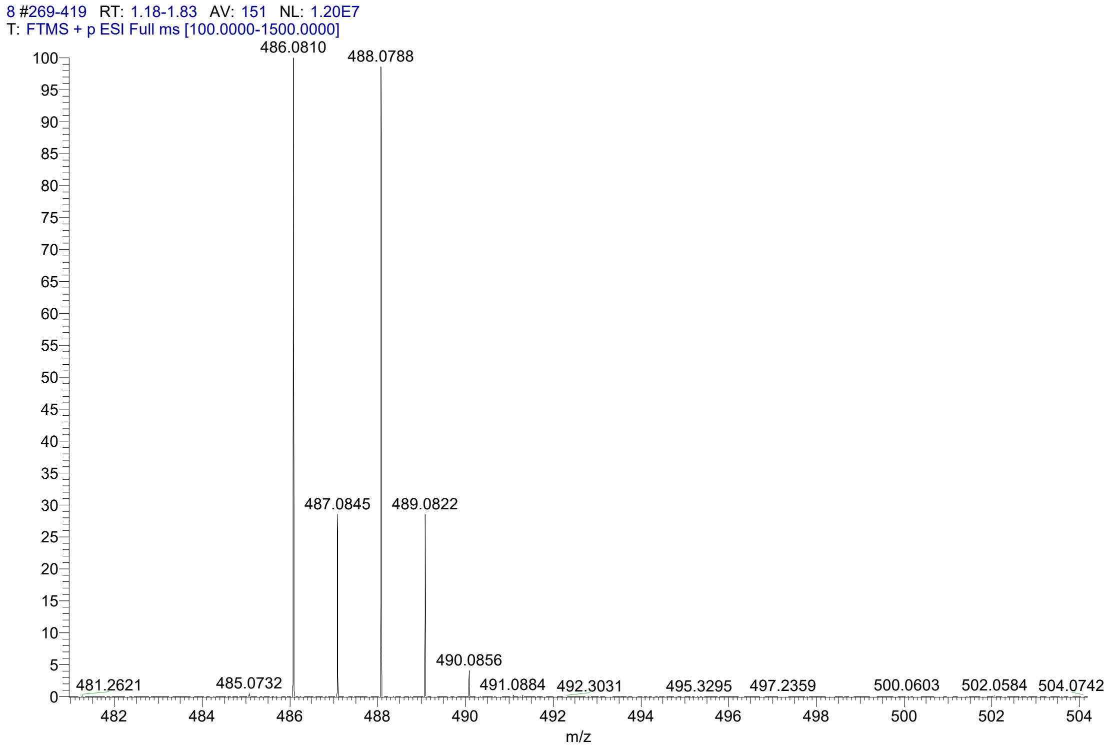

Figure S17. ^{1}H NMR, ^{13}C NMR and HRMS data of **6d**

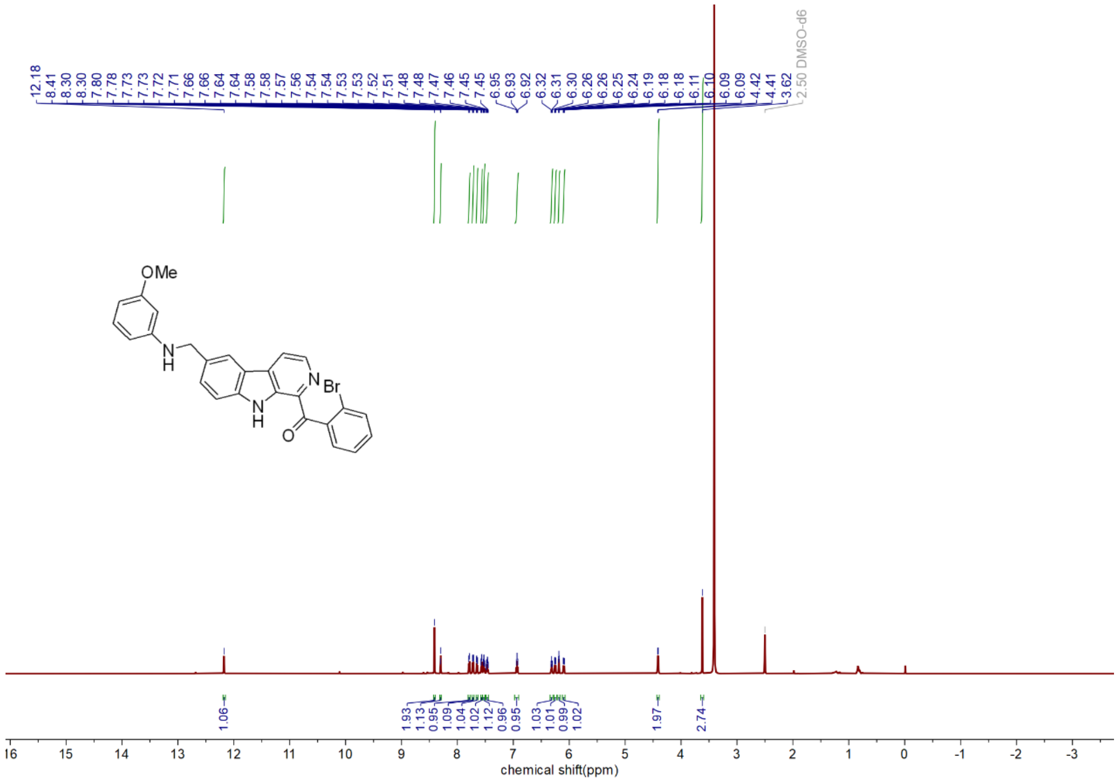

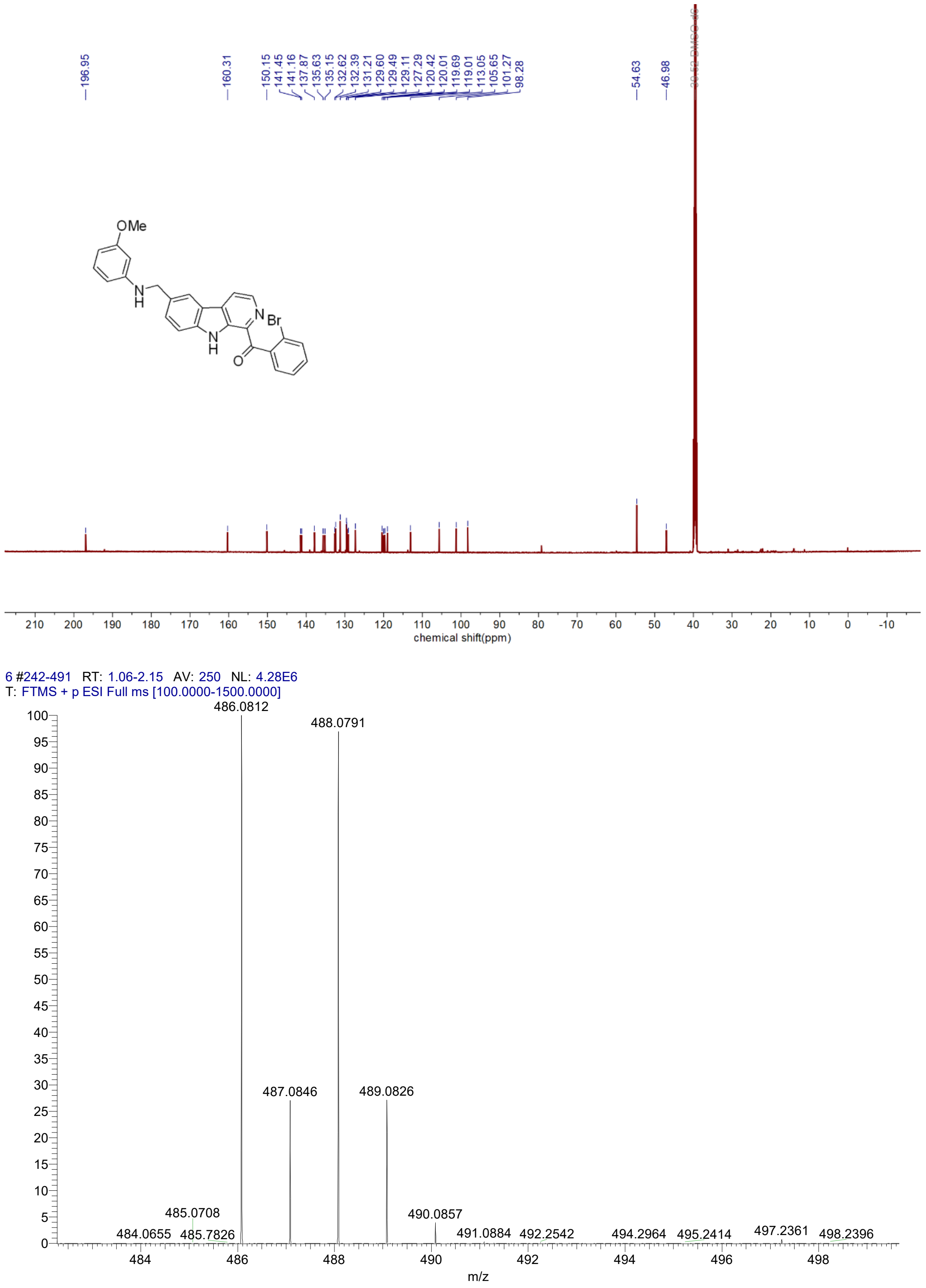

Figure S18. ^{1}H NMR, ^{13}C NMR and HRMS data of **6e**

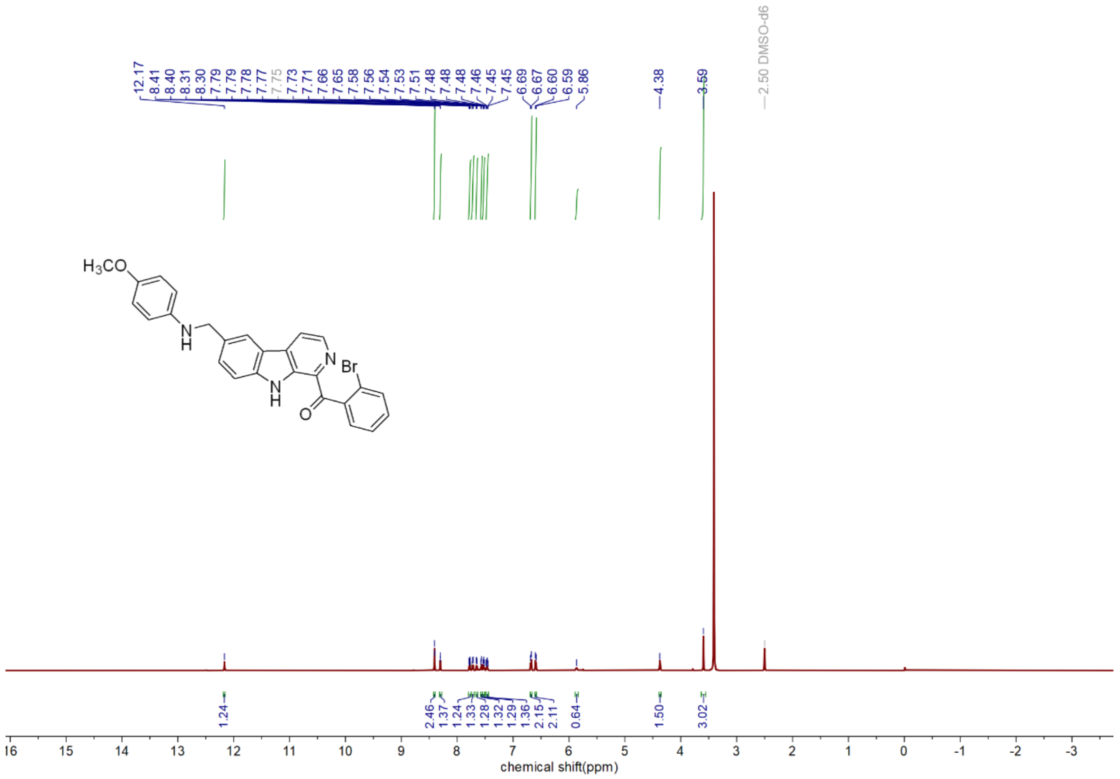

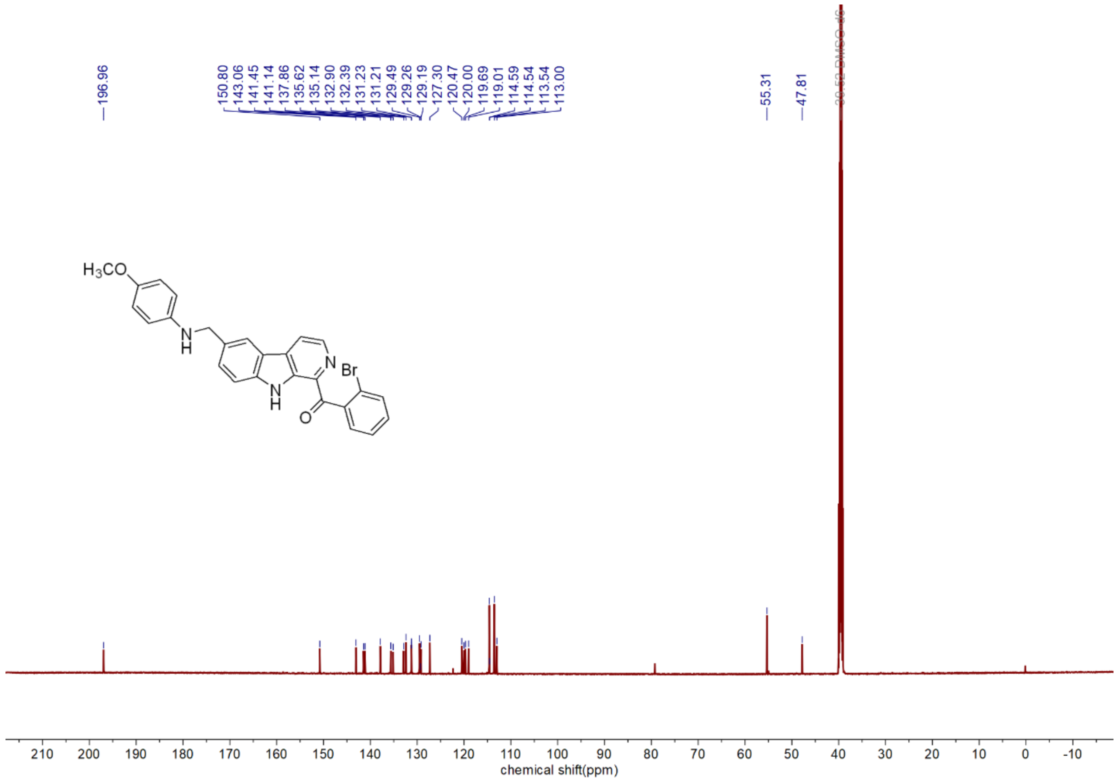

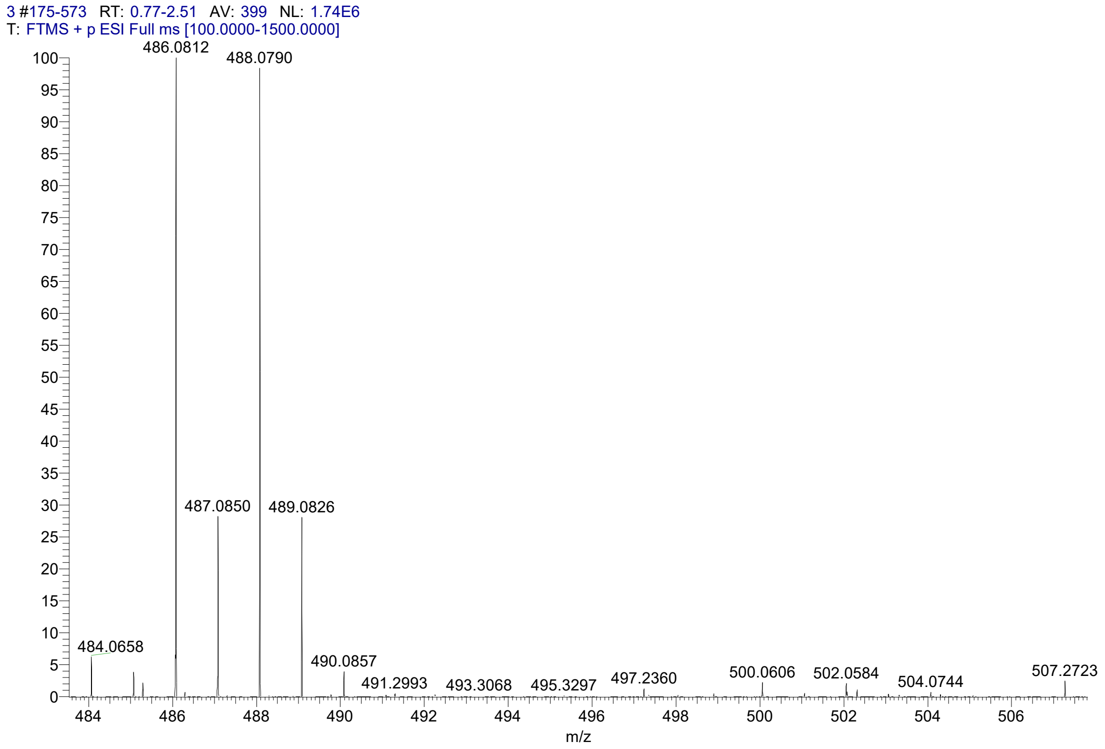

Figure S19. ^{1}H NMR, ^{13}C NMR and HRMS data of **6f**

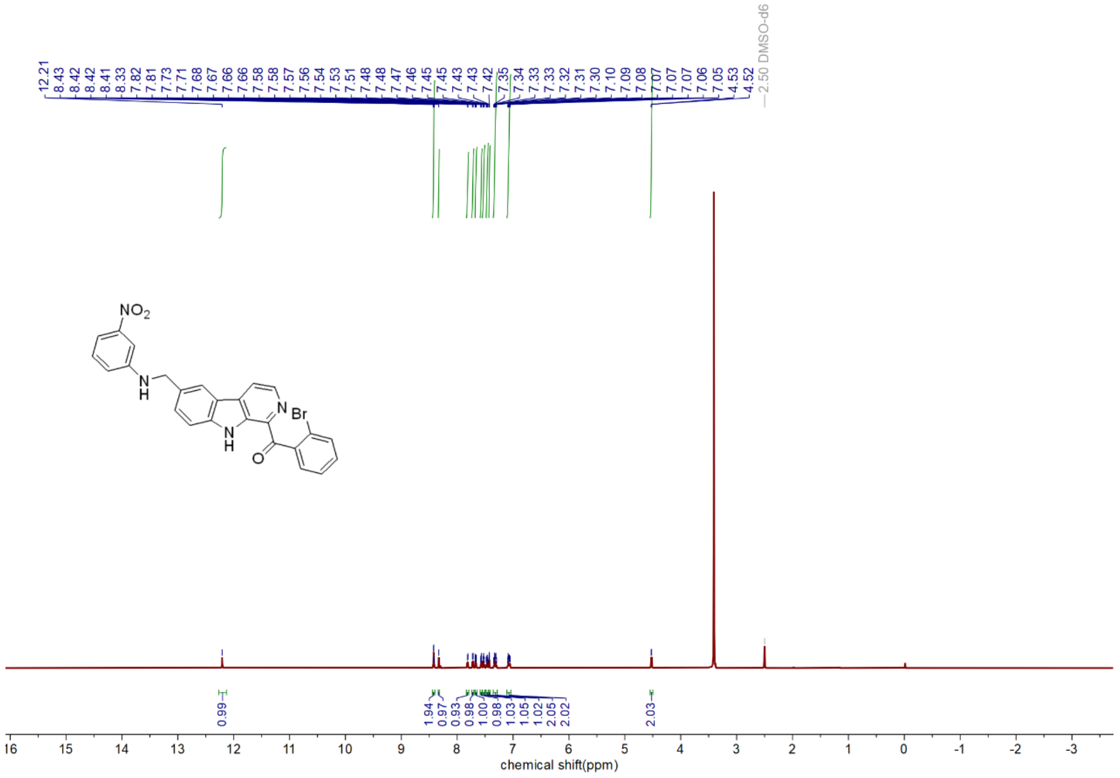

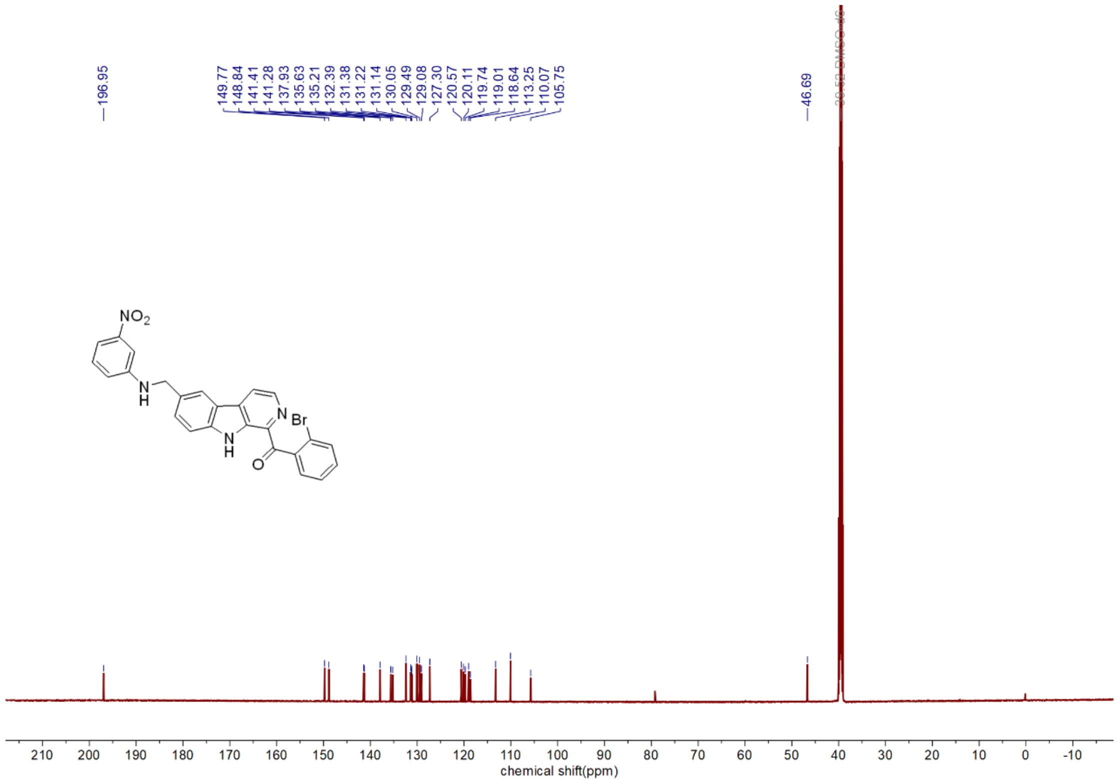
196.95
149.77
148.84
141.41
141.28
137.93
135.63
135.21
132.39
131.38
131.22
131.14
130.05
129.49
129.08
127.30
120.57
120.11
119.74
119.01
118.64
113.25
110.07
105.75
46.69
NO2
N H
N Br
N H
O
210 200 190 180 170 160 150 140 130 120 110 100 90 80 70 60 50 40 30 20 10 0 -10
chemical shift(ppm)

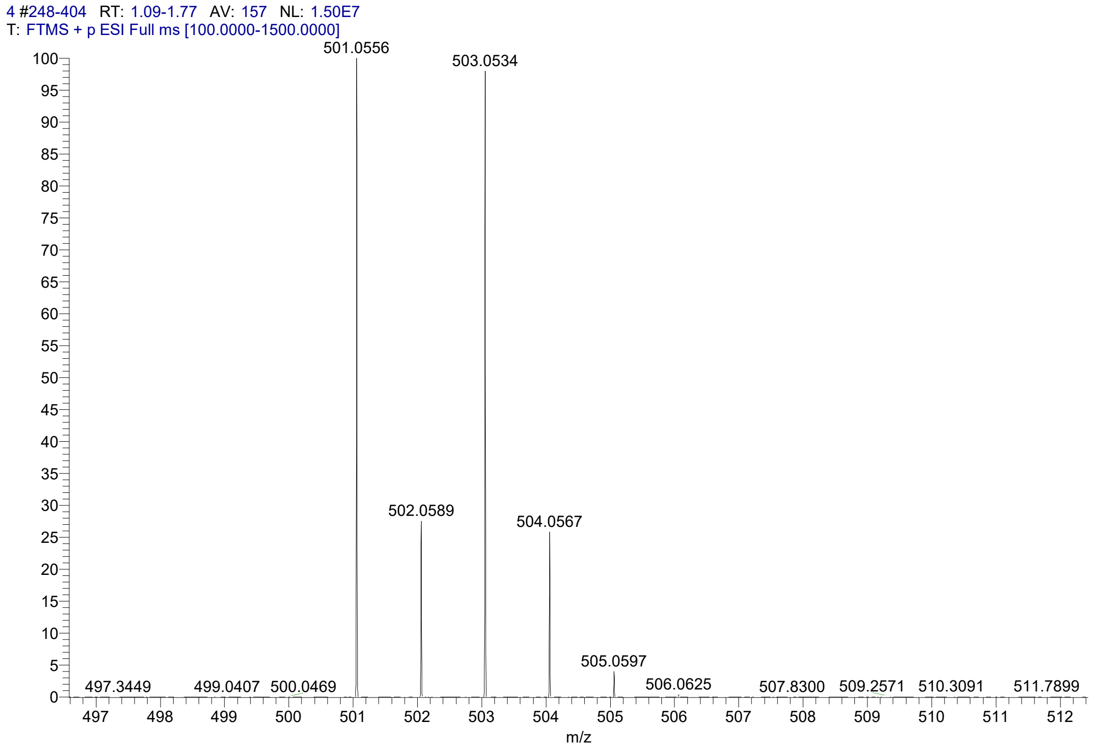
4 #248-404 RT: 1.09-1.77 AV: 157 NL: 1.50E7
T: FTMS + p ESI Full ms [100.0000-1500.0000]
501.0556
503.0534
502.0589
504.0567
505.0597
506.0625
497.3449
499.0407
500.0469
507.8300
509.2571
510.3091
511.7899
m/z

5. HPLC Analysis of Compounds **3a–c**, **4a–e**, **5a–b**, and **6a–f**

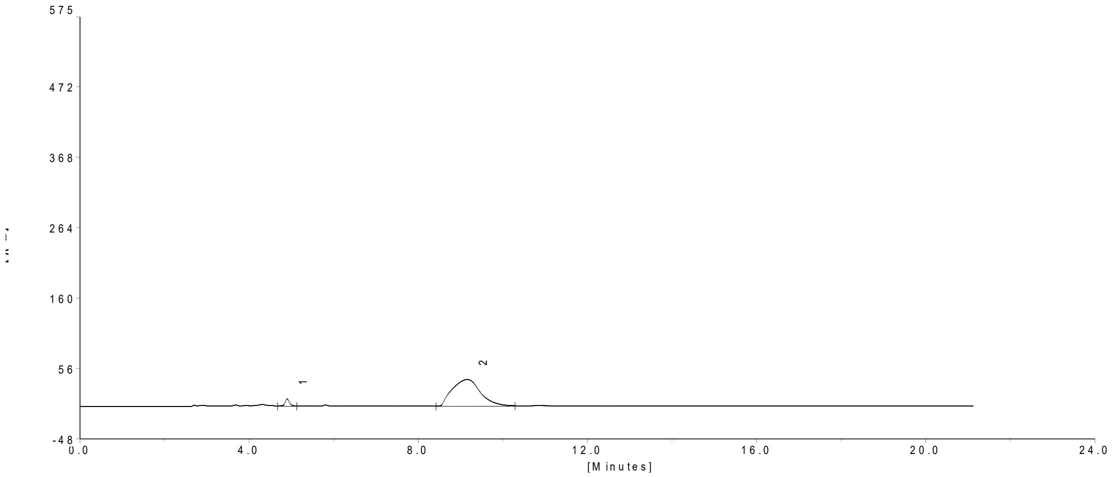

Peak	Ret. (min)	Height	Area	Area%
1	4.8992	10.43	71.63	3.8650
2	9.1558	38.96	1781.62	96.1350

HPLC of compound **3a**.

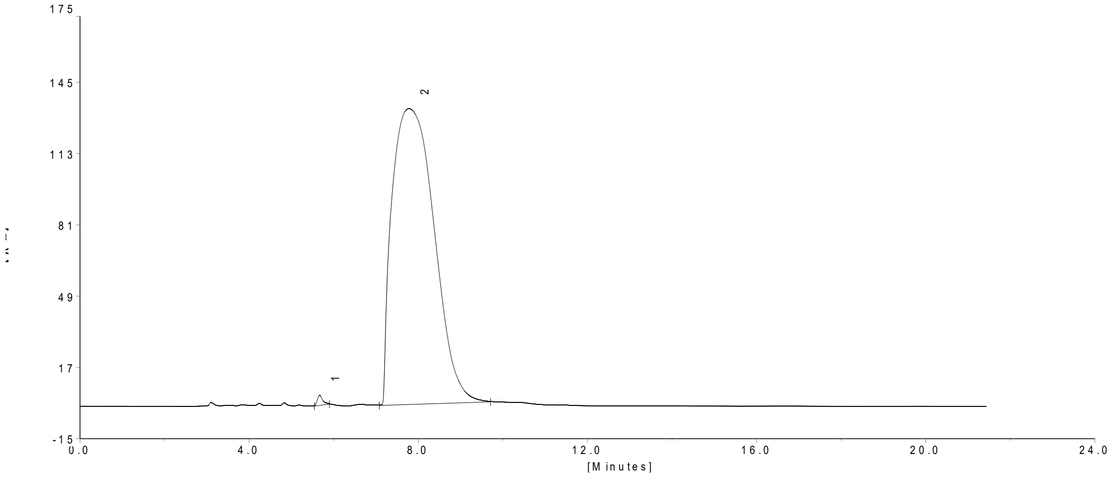

Peak	Ret. (min)	Height	Area	Area%
1	5.6650	4.38	36.68	0.3989
2	7.7775	132.66	9158.43	99.6011

HPLC of compound **3b**.

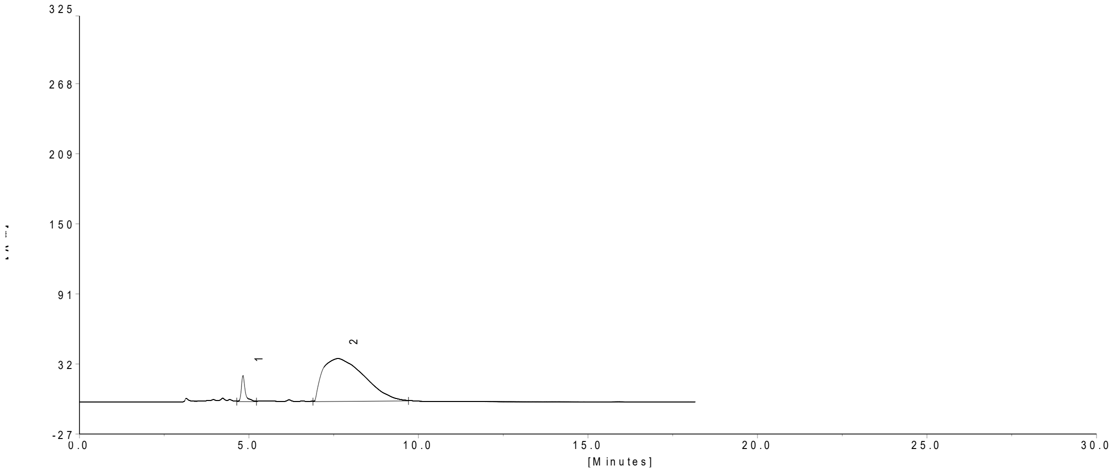

Peak	Ret. (min)	Height	Area	Area%
1	4.8225	21.47	165.36	5.1918
2	7.6208	35.82	3019.65	94.8082

HPLC of compound **3c**.

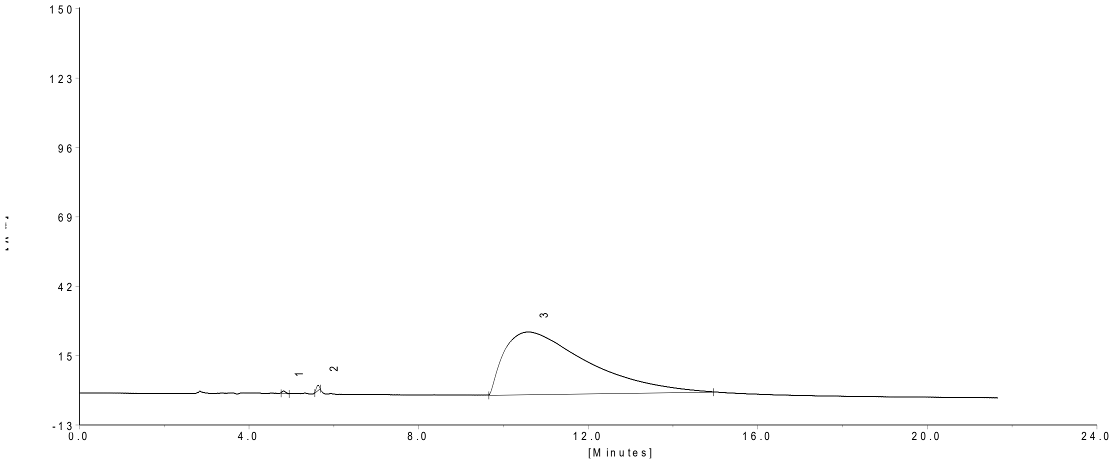

Peak	Ret. (min)	Height	Area	Area%
1	4.8083	0.82	4.46	0.1346
2	5.6308	1.75	8.25	0.2488
3	10.5808	24.30	3302.12	99.6166

HPLC of compound **4a**.

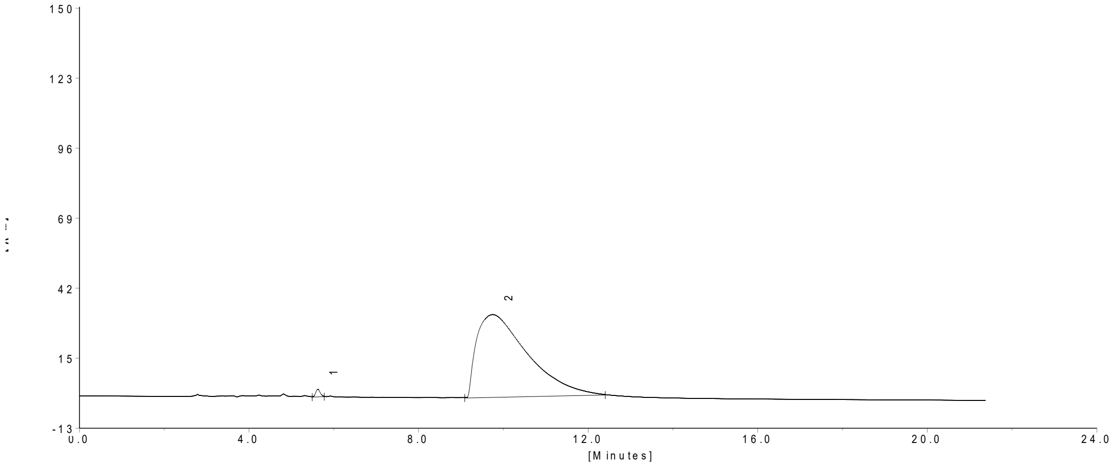

Peak	Ret. (min)	Height	Area	Area%
1	5.6217	2.76	19.43	0.7322
2	9.7492	31.81	2633.77	99.2678

HPLC of compound **4b**.

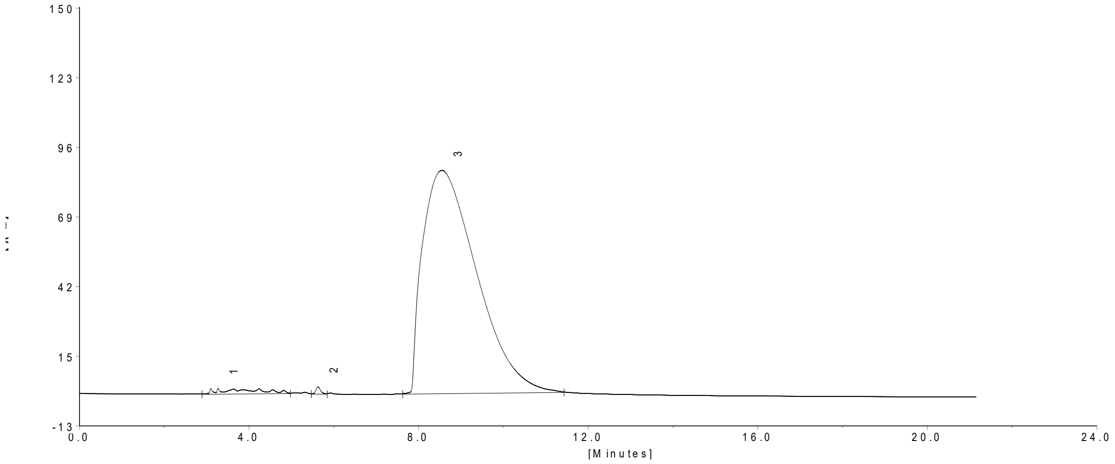

Peak	Ret. (min)	Height	Area	Area%
1	3.2675	2.10	114.51	1.4766
2	5.6250	2.64	18.91	0.2438
3	8.5525	86.47	7621.43	98.2796

HPLC of compound **4c**.

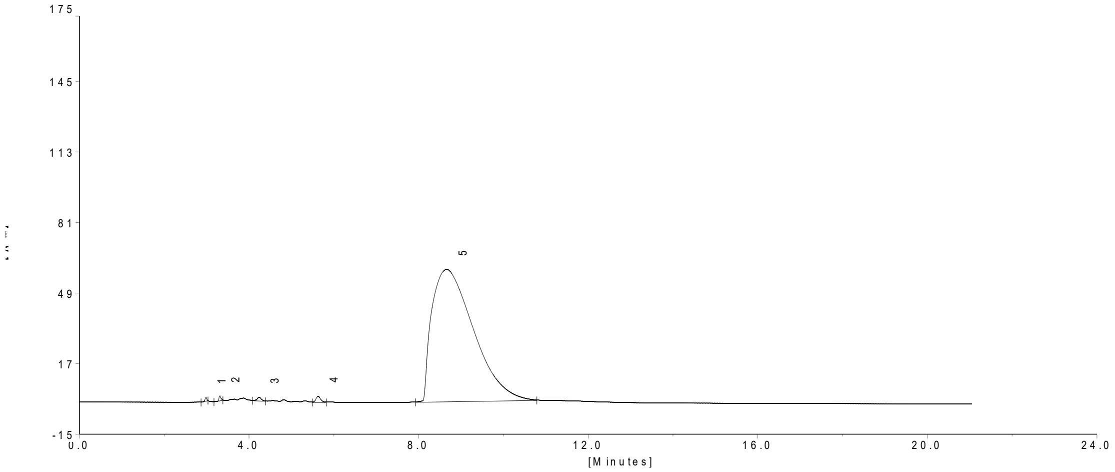

Peak	Ret. (min)	Height	Area	Area%
1	2.9825	1.39	2.97	0.0723
2	3.3092	1.97	4.97	0.1210
3	4.2333	1.50	9.37	0.2284
4	5.6283	2.60	18.75	0.4570
5	8.6675	59.86	4066.85	99.1213

HPLC of compound **4d**.

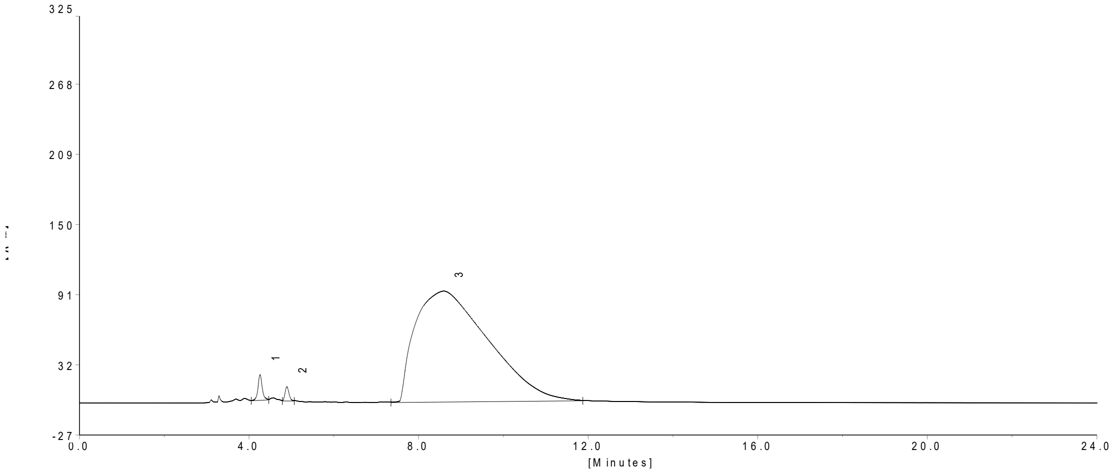

Peak	Ret. (min)	Height	Area	Area%
1	4.2592	21.20	143.72	1.2729
2	4.8917	11.63	72.06	0.6382
3	8.5792	92.95	11075.50	98.0890

HPLC of compound **4e**.

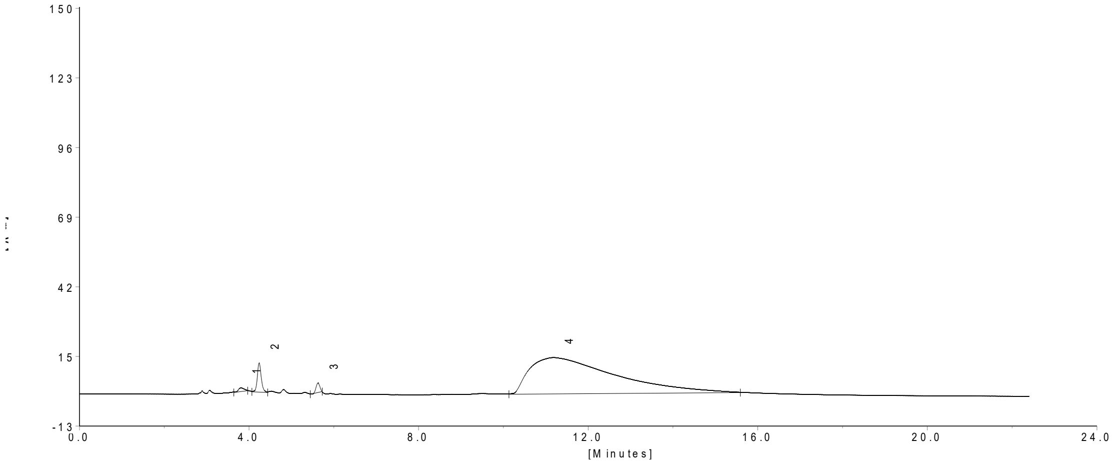

Peak	Ret. (min)	Height	Area	Area%
1	3.8050	1.30	10.19	0.5034
2	4.2383	11.14	70.32	3.4729
3	5.6275	3.49	19.31	0.9536
4	11.1758	13.97	1925.11	95.0701

HPLC of compound **5a**.

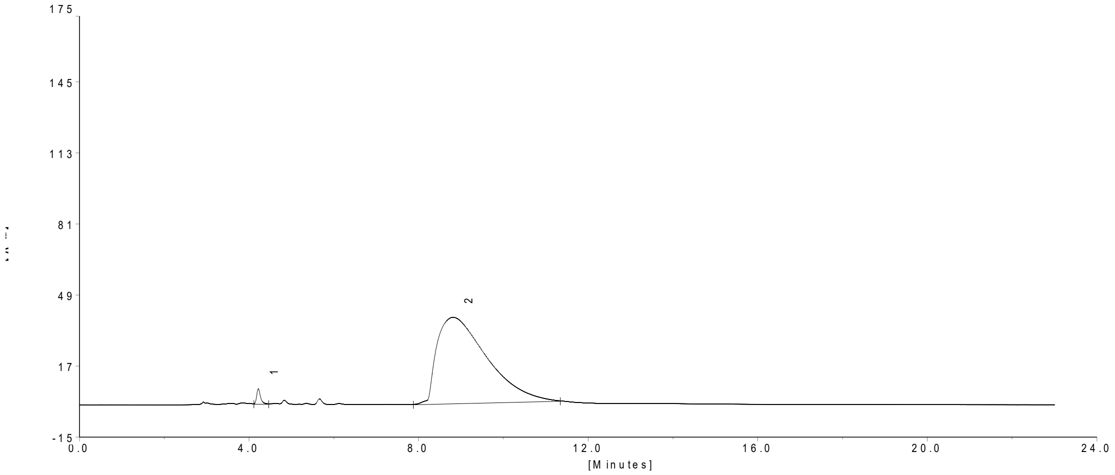

Peak	Ret. (min)	Height	Area	Area%
1	4.2183	6.73	41.25	1.2891
2	8.8066	38.83	3158.74	98.7109

HPLC of compound **5b**.

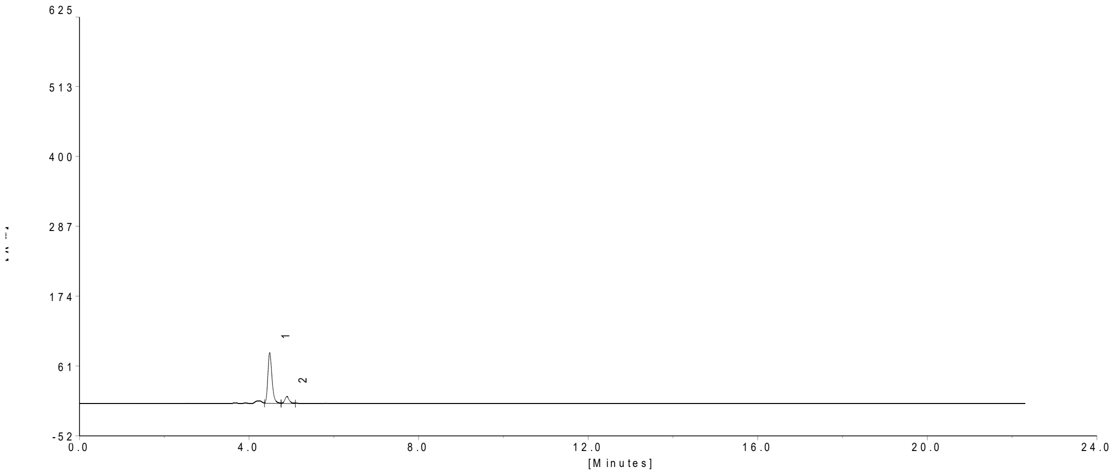

Peak	Ret. (min)	Height	Area	Area%
1	4.4875	81.36	526.08	93.4950
2	4.8925	10.63	36.60	6.5050

HPLC of compound **6a**.

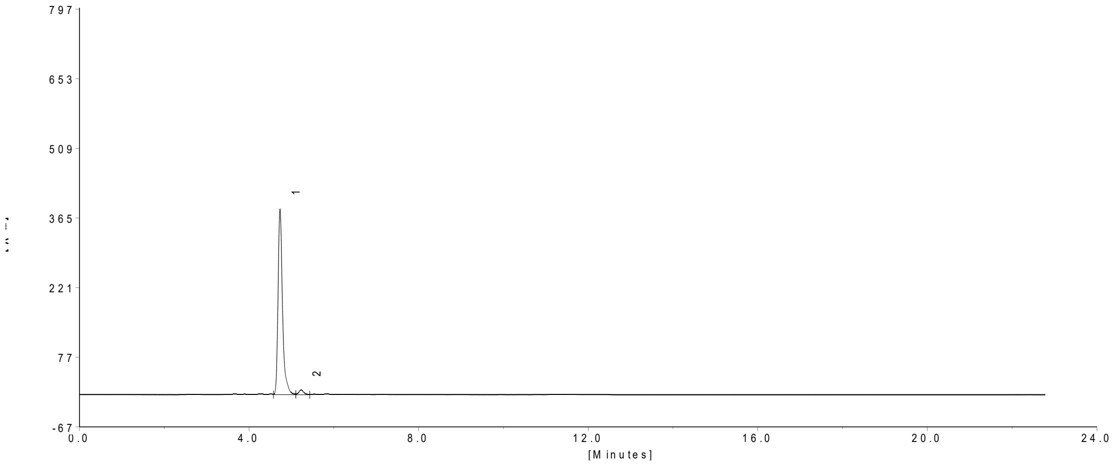

Peak	Ret. (min)	Height	Area	Area%
1	4.7308	383.13	2792.57	97.5911
2	5.2258	8.99	68.93	2.4089

HPLC of compound **6b**.

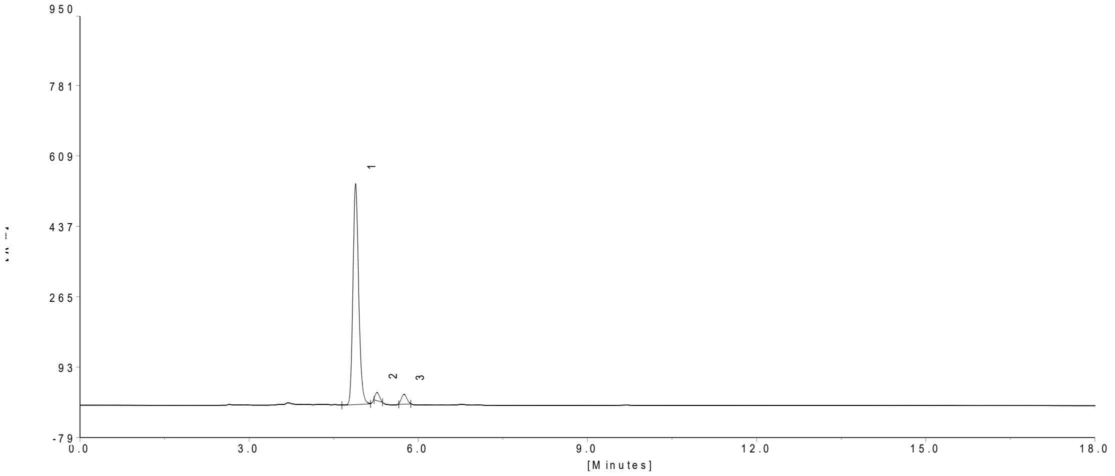

Peak	Ret. (min)	Height	Area	Area%
1	4.8883	539.15	3767.02	93.8079
2	5.2683	19.18	98.82	2.4610
3	5.7483	22.86	149.83	3.7311

HPLC of compound **6c**.

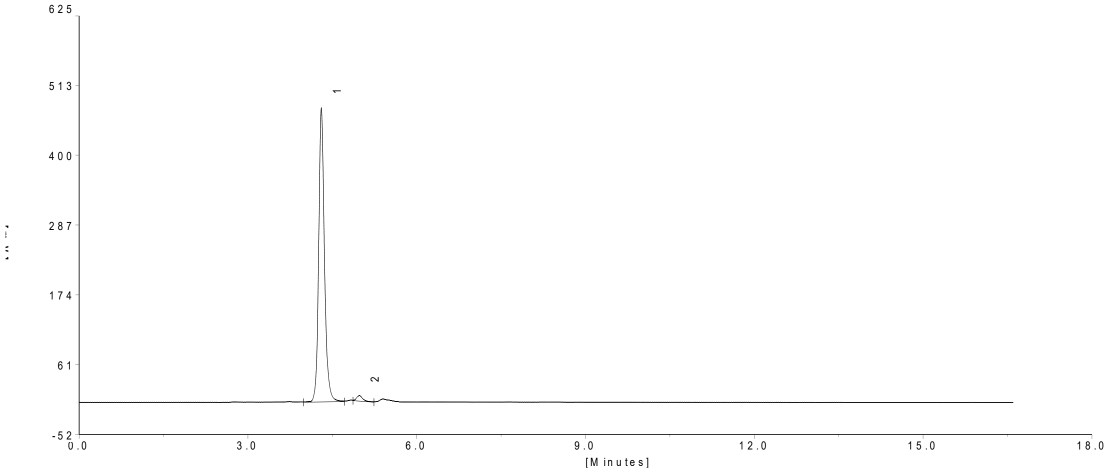

Peak	Ret. (min)	Height	Area	Area%
1	4.3050	475.61	3366.94	98.3700
2	4.9792	8.29	55.79	1.6300

HPLC of compound **6d**.

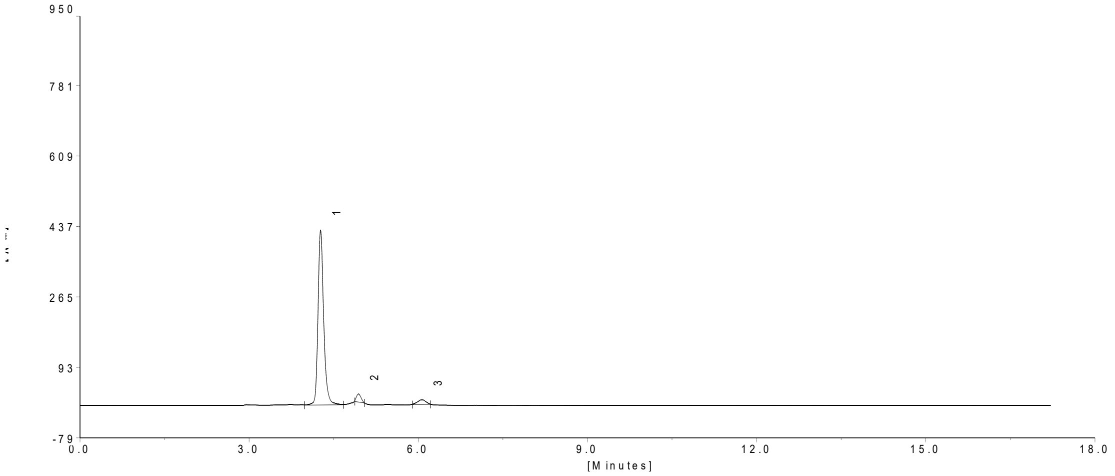

Peak	Ret. (min)	Height	Area	Area%
1	4.2667	426.74	2945.96	93.4501
2	4.9383	18.70	104.38	3.3111
3	6.0658	10.62	102.10	3.2388

HPLC of compound **6e**.

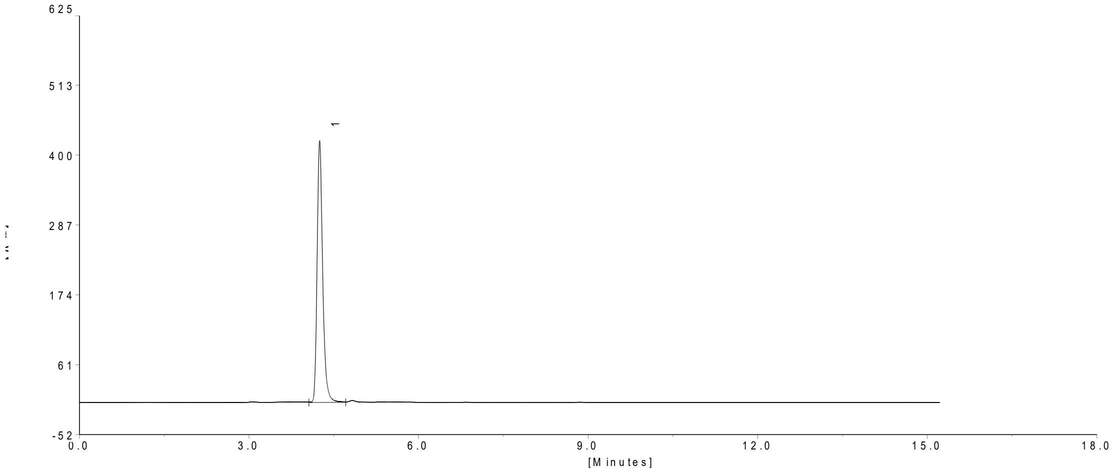

Peak	Ret. (min)	Height	Area	Area%
1	4.2492	422.84	2873.12	100.0000

HPLC of compound **6f**.
